# Prevalence and Risk Factors Associated With Antimicrobial Resistance in Bacteria Related to Bovine Respiratory Disease—A Broad Cross-Sectional Study of Beef Cattle at Entry Into Canadian Feedlots

**DOI:** 10.3389/fvets.2021.692646

**Published:** 2021-07-01

**Authors:** Sara Andrés-Lasheras, Reuben Ha, Rahat Zaheer, Catrione Lee, Calvin W. Booker, Craig Dorin, Joyce Van Donkersgoed, Rob Deardon, Sheryl Gow, Sherry J. Hannon, Steve Hendrick, Michele Anholt, Tim A. McAllister

**Affiliations:** ^1^Lethbridge Research and Development Centre, Agriculture and Agri-Food Canada, Lethbridge, AB, Canada; ^2^Feedlot Health Management Services, Okotoks, AB, Canada; ^3^Veterinary Agri-Health Systems, Airdrie, AB, Canada; ^4^Alberta Beef Health Solutions, Picture Butte, AB, Canada; ^5^Faculty of Veterinary Medicine, University of Calgary, Calgary, AB, Canada; ^6^Department of Mathematics and Statistics, University of Calgary, Calgary, AB, Canada; ^7^Western College of Veterinary Medicine, University of Saskatchewan, Saskatoon, SK, Canada; ^8^Public Health Agency of Canada, Saskatoon, SK, Canada; ^9^Coaldale Veterinary Clinic Ltd., Lethbridge, AB, Canada; ^10^POV Inc., Airdrie, AB, Canada

**Keywords:** antimicrobial resistance, bovine respiratory disease, cross-sectional study, epidemiology, *Mannheimia haemolytica*, *Mycoplasma bovis*, *Pasteurella multocida*, *Histophilus somni*

## Abstract

A broad, cross-sectional study of beef cattle at entry into Canadian feedlots investigated the prevalence and epidemiology of antimicrobial resistance (AMR) in *Mannheimia haemolytica, Pasteurella multocida, Histophilus somni*, and *Mycoplasma bovis*, bacterial members of the bovine respiratory disease (BRD) complex. Upon feedlot arrival and before antimicrobials were administered at the feedlot, deep nasopharyngeal swabs were collected from 2,824 feedlot cattle in southern and central Alberta, Canada. Data on the date of feedlot arrival, cattle type (beef, dairy), sex (heifer, bull, steer), weight (kg), age class (calf, yearling), source (ranch direct, auction barn, backgrounding operations), risk of developing BRD (high, low), and weather conditions at arrival (temperature, precipitation, and estimated wind speed) were obtained. *Mannheimia haemolytica, P. multocida*, and *H. somni* isolates with multidrug-resistant (MDR) profiles associated with the presence of integrative and conjugative elements were isolated more often from dairy-type than from beef-type cattle. Our results showed that beef-type cattle from backgrounding operations presented higher odds of AMR bacteria as compared to auction-derived calves. Oxytetracycline resistance was the most frequently observed resistance across all *Pasteurellaceae* species and cattle types. *Mycoplasma bovis* exhibited high macrolide minimum inhibitory concentrations in both cattle types. Whether these MDR isolates establish and persist within the feedlot environment, requires further evaluation.

## Introduction

Bovine respiratory disease (BRD) continues to be a challenging and costly disease in feedlot cattle in North America (1–3), accounting for 70–80% and 40–50% of the total herd-level morbidity and mortality, respectively (4). Moreover, it has been estimated that BRD costs the North American feedlot cattle industry over $3 billion annually (5). *Mannheimia haemolytica, Pasteurella multocida, Histophilus somni*, and *Mycoplasma bovis* are considered the main bacterial species associated with BRD (6). However, viruses, host, environment, and management practices also play important roles in this multi-factorial, complex disease (7). Considerable resources have been invested in the development of technologies and management strategies to mitigate and treat BRD, but the incidence of morbidity and mortality have remained relatively constant over the last 45 years (8). Practices such as preconditioning, improved diagnostics, and new vaccines continue to be developed, investigated, and validated as alternatives to antimicrobials. However, until these alternatives are shown to be cost-effective, practically implementable, and on-par or surpass currently available practices, it is likely that antimicrobial therapy will continue to be an important tool for preventing, treating, and controlling BRD in feedlot cattle.

Previous studies have suggested that antimicrobial resistance (AMR) amongst BRD-related bacteria has been increasing in North America (9–11). However, these studies mostly collected samples from clinical BRD cases. A previous Alberta study found that 30% of *M. haemolytica* and 12.5% of *P. multocida* isolated from BRD cattle mortalities were resistant to macrolides, tetracyclines (TETs), β-lactams, fluoroquinolones (FQs), lincosamides, aminoglycosides (AMGs), and pleuromutilins (12). Resistance to macrolides is of particular concern due to the importance of these drugs in controlling BRD in high risk incoming cattle (13). Unlike clinical BRD cases, studies that targeted beef cattle at feedlot arrival, prior to antimicrobial administration found that *M. haemolytica* and *P. multocida* were susceptible to fluoroquinolones, macrolides, β-lactams, and cephalosporins (14–16). This suggests that AMR increases after feedlot placement. However, previous published studies that assessed AMR prior to administration of antimicrobials at arrival focused on *M. haemolytica* and not all four members of the BRD bacterial complex (14, 15).

Development of AMR threatens access and efficacy of antimicrobials, has the potential to increase antimicrobial use (AMU) (5), threaten animal health and welfare (17), increase production costs (18), and promote dissemination of antimicrobial-resistance genes (ARG) in cattle and the environment (19). Passive AMR surveillance can help to identify emerging AMR trends (20). However, for a global and non-biased vision of current AMR patterns, epidemiologically robust studies are required. The Canadian beef industry in partnership with CIPARS (Canadian Integrated Program for Antimicrobial Resistance Surveillance) has recently developed a national feedlot AMU/AMR surveillance program targeting selected respiratory nasal bacterial pathogens and enteric bacteria (21). To complement the surveillance program, this study was designed to estimate the current prevalence and describe AMR in *M. haemolytica, P. multocida, H. somni*, and *M. bovis*, and to provide insight into the epidemiology and possible risk factors for AMR prior to cattle arriving at the feedlot.

## Materials and Methods

### Ethics Statement and General Cattle Management

The animal study was reviewed and approved by the Lethbridge Research Centre Animal Care and Use Committee (Protocol #1641, Jan 18th, 2017) and was conducted according to the Canadian Council of Animal Care Guidelines (22).

### Sampling

From August 2017 to May 2018 and from August 2018 to April 2019 (19 months), deep nasopharyngeal swabs (DNPS) were collected from feedlot cattle at processing at feedlot arrival, prior to the administration of antimicrobials. Cattle were housed in ten different commercial feedlots located across southern and central Alberta purposely selected based on their one-time holding capacity, the use of an electronic record keeping system, and willingness to collaborate in the study. Each feedlot (designated A-J) was managed by one of four veterinary practices providing services to ~80% of Alberta's 1.5 million feedlot cattle. Feedlots were selected based on their one-time holding capacity, the use of an electronic animal health record-keeping system, and their willingness to participate. Six feedlots had a one-time capacity >10,000 head; whereas four housed <10,000 head. For sampling, transport truck was considered the sampling unit. When beef-type cattle from different locations were transported in the same truck, location was also considered to define the sampling unit. For sample size calculation, the isolation percentage of BRD bacteria resistant to at least one antimicrobial was expected to by 15% based on previous pilot studies carried out in the same geographical area (JVD, personal communication). Sample size at animal level was calculated before the participating feedlots were assigned. Assuming *n* = ${\text{Z}}_{\text{α}}^{2}$ pq/L^2^; α = 0.05 (Z_α_ = 1.96); *p* = 0.15; and with a precision of L = 0.05, a total of 200 head were initially targeted per feedlot per year. A total of 10 cattle from each of the 10 participating feedlots were targeted for sampling every 2 weeks for 40 weeks (September to May) over two consecutive years. Animals were selected using cluster sampling, with cattle within a transport truck constituting a cluster. During off-loading, ten animals were randomly selected as they passed through the chute. If two or more transport trucks arrived on the same day, two trucks were randomly selected and five animals were sampled from each truck. A single DNPS was collected from each animal by a trained staff member using a guarded swab (BD CultureSwab™ Plus, Mississauga ON). Swabs were shipped on ice in Amies transport media without charcoal (BD CultureSwab™ Plus) to the Lethbridge Research and Development Centre Microbiology Laboratory. Samples were kept at 4°C for up to 7 d prior to isolation of bacteria.

Upon arrival, the electronic ear tag was scanned and the arrival date, cattle type (beef, dairy), sex (heifer, bull, steer), weight (kg), age class (calf, yearling), source (ranch direct, auction barn, backgrounding operation), weather (temperature, precipitation, estimated wind speed), and BRD risk (high, low) were recorded. Ranch-direct cattle were defined as calves that arrived directly from the birth farm immediately after weaning or following a variable feeding period. Backgrounding operation-sourced cattle arrived directly from the backgrounding feedlot where they had been since weaning and fed a forage-based diet until ready for a finishing diet. Auction-derived cattle may have arrived at auction directly from the birth farm or from a backgrounding operation; this earlier history was unknown. Risk of BRD was predicted by the feedlot as per usual practices based on algorithms that consider age class, body weight, source, comingling prior to arrival, weather, transport distance, and visual health assessment. The geographical location of each feedlot, country of origin, and the point of sale at auction were recorded. Unlike dairy-type calves, beef-type calves were not traced back to their farm of origin given that more than 2/3 of the cattle (67.7%) were auction market derived. Morbidity and mortality data were compiled for all enrolled cattle up to 120 d post-arrival. Morbid animals [e.g., displaying gauntness, inactivity, depression, elevated rectal temperature (typically over 40°C), ocular/nasal discharge, cough, and/or elevated respiration rate] were designated as having BRD by trained feedlot pen riders or animal health personnel. Information on AMU before feedlot placement was unavailable.

### Bacterial Isolation and Species Identification

Upon arrival at the laboratory, swabs were individually immersed in 1 mL of brain-heart infusion (BHI) containing 20% glycerol (Dalynn Biologicals, Calgary AB) and vortexed for 1 min (14). For the isolation of *M. haemolytica* and *P. multocida*, 100 μL suspension was plated onto blood agar (TSA) with 5% sheep blood supplemented with bacitracin at 15 μg/mL (Dalynn Biologicals). An additional 100 μL aliquot was plated onto TSA blood agar (Dalynn) for the isolation of *H. somni*. Additionally, a 1:10 dilution of the initial BHI-glycerol suspension was plated (100 μL) on TSA blood agar plates to enable isolation of *H. somni* without overgrowth. Bacitracin plates were incubated in an aerobic atmosphere at 37°C for 24 h, before selecting *P. multocida* and *M. haemolytica* colonies. *Histophilus somni* plates were incubated at 37°C for 2 d in a 5% CO_2_ atmosphere. Presumptive *M. haemolytica, P. multocida*, or *H. somni* colonies were identified, and three colonies of each bacterial species were sub-cultured onto TSA blood agar. Colonies were picked from TSA and stored at −80°C in BHI supplemented with 20% glycerol. For *M. bovis*, a high throughput PCR-based enrichment process was used for screening for the presence of *M. bovis* followed by bacterial isolation from positive suspensions (23).

All bacterial isolates were confirmed using PCR species-specific primers (Table 1) using a *HotStartTaq Plus Master Mix* kit (Qiagen, Toronto ON). For this, 3–5 colonies of pure cultures of *P. multocida, M. haemolytica*, or *H. somni* were suspended in 100 μL of TE buffer (10 mM Tris, 1 mM EDTA, pH = 8) and heat-lysed for 5 min at 95°C. The suspension was centrifuged for 10 min, at 18,400 × g at 4°C, and 2 μL of the supernatant was used as a DNA template. Presumptive *M. bovis* cultures were confirmed via direct-culture-PCR using 2 μL of liquid culture directly in the PCR master mix (23). The PCR amplicons were visualized either by agarose gel or capillary electrophoresis (QIAxcel, Qiagen). One randomly selected colony per bacterial species per DNPS was selected for further characterization and analysis. Additionally, *M. haemolytica* isolates were further characterized as serotype A1, A2 or A6 using a multiplex PCR (Table 1) (27).

**Table 1:** Oligonucleotide primers, PCR protocols, and amplicon sizes for each PCR assay used in this study.

**Bacterial species and targeted gene**	**Primer sequences (5'−3') and PCR cycling conditions**		**Amplicon size (bp)**	**References**
Mh, *lktC*- *artJ* intergenic region	F – GTCCCTGTGTTTTCATTATAAG R – ACTCGATAATTATTCTAAATTAG 95°C, 5 min; (94°C, 30 s; 58°C, 45 s; 72°C, 60 s) × 35 cycles; 72°C, 10 min		385	(24)
Pm, 23S rRNA	F – GGCTGGGAAGCCAAATCAAAG R – CGAGGGACTACAATTACTGTAA 95°C, 5 min; (94°C, 30 s; 58°C, 45 s; 72°C, 60 s) × 35 cycles; 72°C, 10 min		1,432	(25)
Hs, 16S rRNA	F – GAAGGCGATTAGTTTAAGAG R – TTCGGGCACCAAGTRTTCA 95°C, 5 min; (94°C, 30 s; 55°C, 45 s; 72°C, 60 s) × 35 cycles; 72°C, 10 min		400	(26)
Mb, *uvrC*	F – CCTGTCGGAGTTGCAATTGT R – GCACTGCGCTCATTTAAAGC 95°C, 5 min^a^; (94°C, 30 s; 61.5°C, 30 s; 72°C, 30 s) × 35 cycles, 72°C, 10 min		92	(23)
Mh serotype A1, hypothetical protein	F – CATTTCCTTAGGTTCAGC R – CAAGTCATCGTAATGCCT	95°C, 15 min; (94°C, 30 s; 55°C, 45 s; 72°C, 1 min) × 35 cycles; 72°C, 10 min	306	(27)
Mh serotype A2, core-2/I-branching enzyme	F – GGCATATCCTAAAGCCGT R – AGAATCCACTATTGGGCACC		160	
Mh serotype A6, *TupA*	F – TGAGAATTTCGACAGCACT R – ACCTTGGCATATCGTACC		78	

a *Denaturation time increased from 5 min to 10 min if cell lysis was required during the PCR cycle*.

*Mh, Mannheimia haemolytica; Pm, Pasteurella multocida; Hs, Histophilus somni; Mb, Mycoplasma bovis; F, forward primer; R, reverse primer*.

### Antimicrobial Susceptibility Testing

Due to the high recovery of *P. multocida* and the cost of *M. bovis* ASTs, antimicrobial susceptibilities were only performed on a subset of *P. multocida* (*n* = 273/703 beef and *n* = 242/463 dairy isolates) and *M. bovis* (*n* = 122/257 beef and *n* = 100/198 dairy) isolates, whereas all *M. haemolytica* (*n* = 281 beef and *n* = 209 dairy) and *H. somni* (*n* = 68 and *n* = 173 dairy) isolates were tested. *Pasteurella multocida* and *M. bovis* isolates were selected based on proportional stratified random sampling (28) with consideration of feedlot, sampling month, cattle type, and country of origin. The antimicrobial susceptibility testing of *M. haemolytica, P. multocida*, and *H. somni* was carried out by broth microdilution according to Clinical and Laboratory Standards Institute (CLSI) guidelines using a final bacterial inoculum of ~5 × 10^5^ CFU/mL (29). The Sensititre plate BOPO6F was used (Thermo Fisher Scientific, Mississauga ON), which represents a broad-spectrum of antimicrobials commonly used in beef cattle production. For *M. haemolytica* and *P. multocida*, the final per-well bacterial inoculum volume was 50 μL, whereas for *H. somni*, the volume was doubled to 100 μL as per manufacture's specifications. Therefore, the final antimicrobial concentrations tested for *H. somni* were half that of *M. haemolytica* and *P. multocida* (Supplementary Tables 1.1–1.3). Considering that the maximum spectinomycin (SPE) concentration against *H. somni* in BOPO6F plate was only 32 μg/mL, a custom plate with SPE ranging from 8 to 512 μg/mL was prepared for susceptibility testing. Bacterial isolates were designated as susceptible, intermediate, or resistant (SIR) according to CLSI (30). Additionally, CLSI MIC interpretive criteria for tilmicosin (TIL) in *M. haemolytica* was extrapolated to *P. multocida* and *H. somni* (31). Interpretive criteria for danofloxacin from *M. haemolytica* and *P. multocida* was also extrapolated to *H. somni* (32).

For *M. bovis*, recommendations for broth microdilution antimicrobial susceptibility were followed as official international guidelines are unavailable (11, 33, 34). A custom Sensititre plate (ref. no. YCML2FMBC, Thermo Fisher Scientific) was used to assess isolate sensitivities (Supplementary Table 1.4) (11). *Mycoplasma bovis* isolates were grown in PPLO broth and suspensions were adjusted to obtain a final concentration of 10^3^-10^5^ CFU/mL per well (100 μL final volume). Plates were incubated for 48 h, at 37°C, in a 5% CO_2_ atmosphere (35). AlamarBlue™ (Cell Viability Reagent. Thermo Fisher Scientific) at a final concentration of 10% was included in each well as indicator of cell viability and growth. *Mycoplasma bovis* ATCC 25523 was included as an internal standard in all susceptibility assays. Since CLSI breakpoints for *M. bovis* have not been developed, only MICs were reported.

Multidrug resistance (MDR) was defined as resistance to three or more antimicrobial drug classes (36). Isolates with intermediate susceptibility to an antimicrobial were not included in this definition. The 50th and 90th percentiles for MIC (MIC_50_ and MIC_90_, respectively) were calculated as the antimicrobial concentrations required to inhibit 50 and 90% of the isolates within each bacterial species. If growth was observed at the highest antimicrobial concentration tested, the MIC was assigned to the next dilution.

### Antimicrobial Resistance Genetic Determinants

A subset of *M. haemolytica* (*n* = 87), *P. multocida* (*n* = 64), and *H. somni* (*n* = 24) isolates were selected for whole-genome sequencing (WGS). Isolates were selected to represent the diversity of feedlot operations, geographical origin, cattle type, sex, time of the year, and AMR phenotypes using proportional stratified random sampling. To isolate genomic DNA, bacterial cultures grown on blood agar plates were suspended in TE buffer (10 mM Tris – 1 mM EDTA, pH 8.0) to an OD_600_ of ~2, representing ~2 × 10^9^ CFU/ mL. The cell suspension (1 mL) was transferred to a microcentrifuge tube and centrifuged for 2 min at 14,000 × g. Genomic DNA was extracted from the cell pellet using a DNeasy Blood and Tissue kit (Qiagen, Montreal QC). DNA quality and quantity were confirmed using a Nanodrop 2000 spectrophotometer and a fluorometer using PicoGreen (Thermo Fisher Scientific), respectively. Genomic library construction was performed using the NEB Ultra II library preparation kit (New England BioLabs, Ipswich MA). Library quality was assessed using a Bioanalyzer (Agilent, Santa Clara CA). Libraries were sequenced on an Illumina HiSeqX platform to generate 2 × 150 base paired-end reads. Library preparation and Illumina sequencing services were provided by Genome Quebec (McGill, QC). The quality of raw sequence reads was assessed using the quality control tool FastQC and reads were assembled into contigs using the Shovill pipeline (37). This pipeline included a quality trimming step with Trimmomatic (v.0.38) to remove common Illumina adapter sequences followed by *de novo* assembly with SPAdes (v.3.13.0) (38). The assembled contigs were annotated with Prokka (39) to identify coding genes. The assembled contigs were searched against the NCBI Bacterial Antimicrobial Resistance Reference Gene database (NCBI BioProject ID: PRJNA313047) to identify AMR genes. The genome sequencing data of isolates used in this study have been submitted to the NCBI under BioProject ID: PRJNA720670.

### Data Analysis

**Figure 1:**
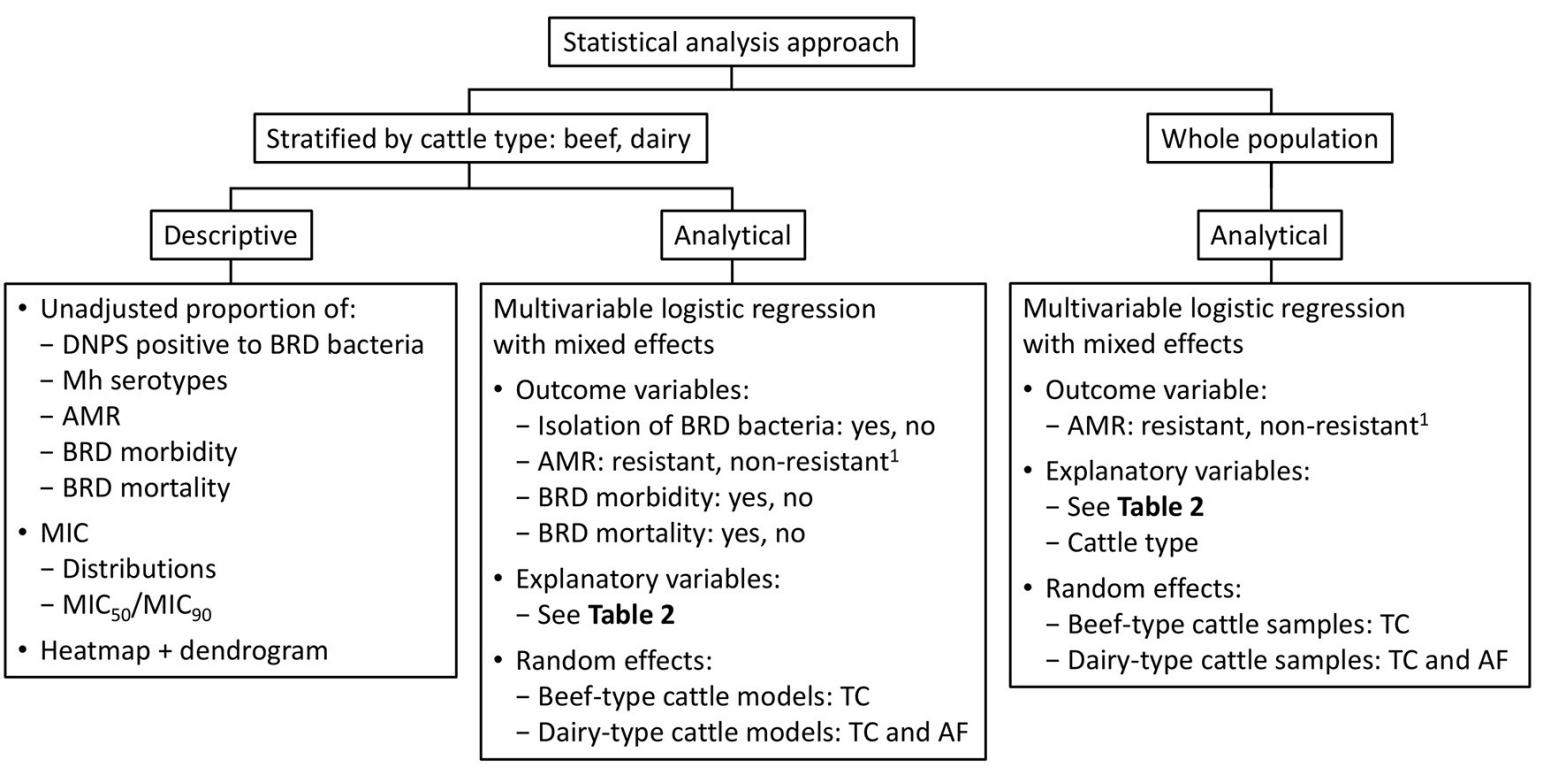
Statistical analyses used in the present study. ^1^Non-resistant category includes intermediate and susceptible categories. AF, arrived from; AMR, antimicrobial resistance; BRD, bovine respiratory disease; DNPS, deep nasopharyngeal swab; Mh, *Mannheimia haemolytica*; MIC, minimum inhibitory concentration; TC, truck cluster.

Analyses were stratified by cattle type (beef or dairy) and included a descriptive examination of the unadjusted proportion of the following outcomes: DNPS positive for BRD bacteria, *M. haemolytica* serotypes, CLSI-determined resistance percentages, and BRD morbidities and mortalities (Figure 1). The unadjusted proportions of these outcomes were compared by cattle type using a chi-square or, when there were <5 samples in a stratum, the Fisher's Exact test (STATCALC, EpiInfo v.7.2.3.1). Antimicrobial susceptibility data were also presented as MIC frequency distributions and as MIC_50_ and MIC_90_ values. For *M. bovis*, only MIC frequency distributions and MIC_50_/MIC_90_ values were reported.

**Figure 2:**
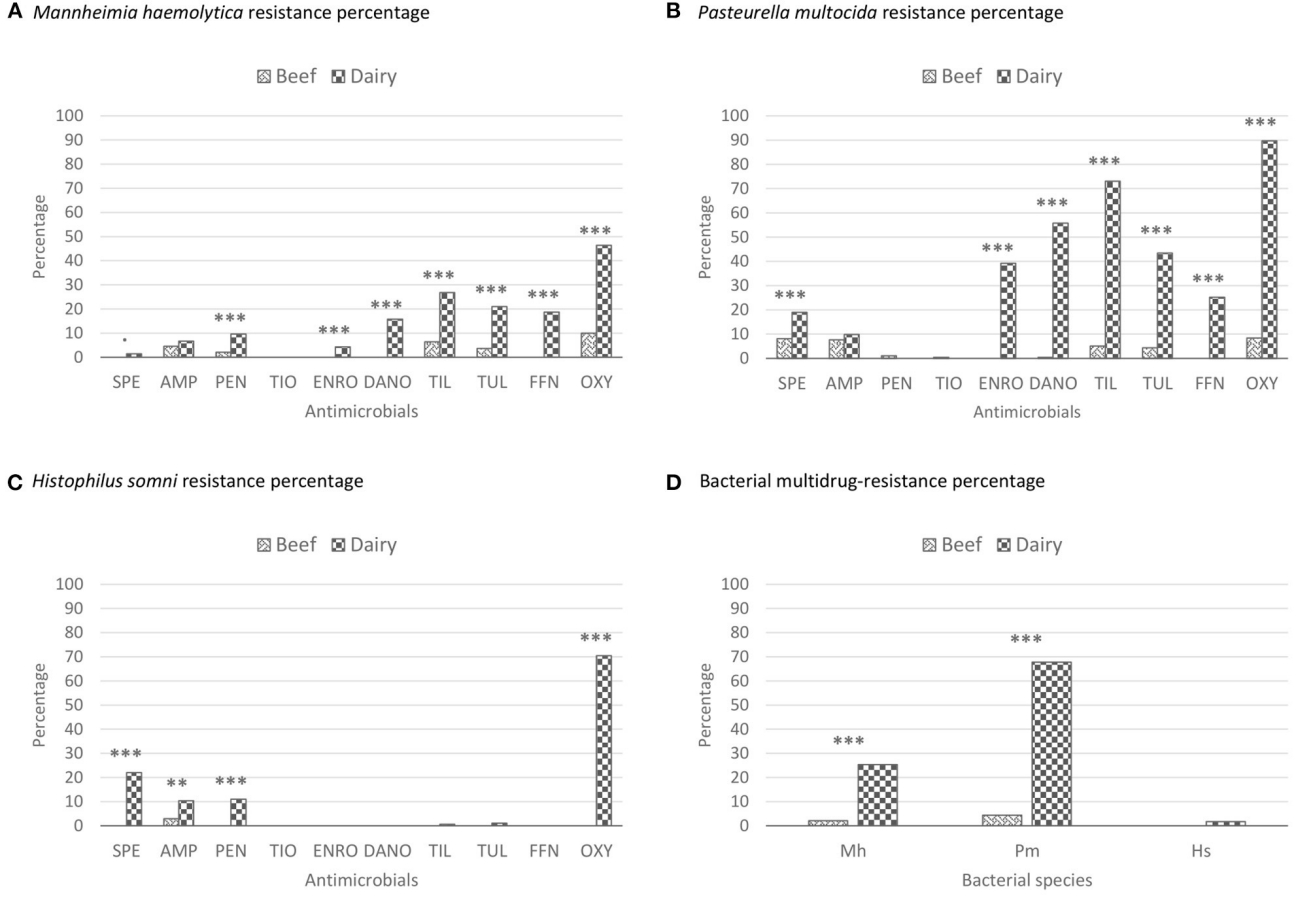
Antimicrobial resistance percentages of the BRD-bacterial isolates recovered from beef and dairy-type cattle upon feedlot arrival. These percentages represent unadjusted proportions. The asterisks represent the statistical test significance levels as follows: “.” 0.1, “**” 0.01, “***” 0.001. Multidrug resistance was defined as resistance to 3 or more different antimicrobial classes (36). AMP, ampicillin; BRD, bovine respiratory disease; DANO, danofloxacin; ENRO, enrofloxacin; FFN, florfenicol; Hs, *Histophilus somni*; Mb, *Mycoplasma bovis*; Mh, *Mannheimia haemolytica*; OXY, oxytetracycline; PEN, penicillin; Pm, *Pasteurella multocida*; SPE, spectinomycin; TIL, tilmicosin; TIO, ceftiofur; TUL, tulathromycin. **(A)** Beef SPE resistance (R): 0/281; beef AMP-R: 13/281; beef PEN-R: 6/281; beef TIO-R: 0/281; beef ENRO-R: 0/281; beef DANO-R: 0/281; beef TIL-R: 18/281; beef TUL-R: 10/281; beef FFN-R: 0/281; beef OXY-R: 28/281; dairy SPE-R: 3/209; dairy AMP-R: 14/209; dairy PEN-R: 20/209; dairy TIO-R: 0/209; dairy ENRO-R: 9/209; dairy DANO-R: 33/209; dairy TIL-R: 56/209; dairy TUL-R: 44/209; dairy FFN-R: 39/209; dairy OXY-R: 97/209. SPE *X*^2^ (1, *n* = 409) = 4.06, *p* = 0.0440; AMP *X*^2^ (1, *n* = 409) = 0.99, *p* = 0.3201; PEN *X*^2^ (1, *n* = 409) = 13.18, *p* < 0.001; ENRO *X*^2^ (1, *n* = 409) = 12.33, *p* < 0.001; DANO *X*^2^ (1, *n* = 409) = 47.57, *p* < 0.001; TIL *X*^2^ (1, *n* = 409) = 38.86, *p* < 0.001; TUL *X*^2^ (1, *n* = 409) = 37.41, *p* < 0.001; FFN *X*^2^ (1, *n* = 409) = 56.97, *p* < 0.001; OXY *X*^2^ (1, *n* = 409) = 83.79, *p* < 0.001. **(B)** Beef SPE-R: 22/273; beef AMP-R: 21/273; beef PEN-R: 3/273; beef TIO-R: 1/273; beef ENRO-R: 0/273; beef DANO-R: 1/273; beef TIL-R: 14/273; beef TUL-R: 12/273; beef FFN-R: 0/273; beef OXY-R: 23/273; dairy SPE-R: 46/242; dairy AMP-R: 24/242; dairy PEN-R: 0/242; dairy TIO-R: 0/242; dairy ENRO-R: 95/242; dairy DANO-R: 135/242; dairy TIL-R: 177/242; dairy TUL-R: 105/242; dairy FFN-R: 61/242; dairy OXY-R: 217/242. SPE *X*^2^ (1, *n* = 515) = 13.42, *p* < 0.001; AMP *X*^2^ (1, *n* = 515) = 0.80, *p* = 0.3722; PEN *X*^2^ (1, *n* = 515) = 2.67, *p* = 0.1482; TIO *X*^2^ (1, *n* = 515) = 0.89, *p* = 0.2650; ENRO *X*^2^ (1, *n* = 515) = 131.41, *p* < 0.001; DANO *X*^2^ (1, *n* = 515) = 202.73, *p* < 0.001; TIL *X*^2^ (1, *n* = 515) = 254.32, *p* < 0.001; TUL *X*^2^ (1, *n* = 515) = 111.09, *p* < 0.001; FFN *X*^2^ (1, *n* = 515) = 78.06, *p* < 0.001; OXY *X*^2^ (1, *n* = 515) = 340.27, *p* < 0.001. **(C)** Beef SPE-R: 0/68; beef AMP-R: 2/68; beef PEN-R: 0/68; beef TIO-R: 0/68; beef ENRO-R: 0/68; beef DANO-R: 0/68; beef TIL-R: 0/68; beef TUL-R: 0/68; beef FFN-R: 0/68; beef OXY-R: 0/68; dairy SPE-R: 38/173; dairy AMP-R: 23/173; dairy PEN-R: 19/173; dairy TIO-R: 0/173; dairy ENRO-R: 0/173; dairy DANO-R: 0/173; dairy TIL-R: 1/173; dairy TUL-R: 2173/; dairy FFN-R: 0/173; dairy OXY-R: 122/173. SPE *X*^2^ (1, *n* = 241) = 17.73, *p* < 0.001; AMP *X*^2^ (1, *n* = 241) = 5.63, *p* = 0.0063; PEN *X*^2^ (1, *n* = 241) = 8.11, *p* < 0.001; TIL *X*^2^ (1, *n* = 241) = 0.69, *p* = 0.3589; TUL *X*^2^ (1, *n* = 241) = 0.79, *p* = 0.2572; OXY *X*^2^ (1, *n* = 241) = 97.12, *p* < 0.001. **(D)** Beef Mh MDR: 6/281; beef Pm MDR: 12/274; beef Hs MDR: 0/68; dairy Mh MDR: 53/209; dairy Pm MDR: 164/242; dairy Hs MDR: 3/173. *X*^2^ (1, *n* = 409) = 61.04, *p* < 0.001; *X*^2^ (1, *n* = 515) = 137.84, *p* < 0.001; *X*^2^ (1, *n* = 241) = 1.19, *p* = 0.1810.

Multivariable logistic regression with mixed effects was used to describe the relationship between epidemiological risk factors and outcomes (Figure 1, Table 2), with control for confounding and clustered data. Outcomes included the isolation of BRD-related bacteria (yes, no), antimicrobial resistance [resistant bacteria vs. non-resistant (susceptible and intermediate) bacteria], BRD morbidity (yes, no) and BRD mortality (yes, no). Transport trailer was considered the sampling unit with animals from the same truck-load considered a cluster (TC). The majority of beef-type cattle were sourced through auction marts whereas most dairy-type cattle were farm-direct. When auction mart beef cattle were from different locations, but transported in the same truck, the random effect was nested (*arrived from* within TC). For dairy-type cattle models, 2 random effects were included (TC and *arrived from*) since the location of their origin was known. Random effects that did not account for variance within the model were removed. The variable feedlot was collinear with one-time feedlot capacity in morbidity and mortality models and as a result, it was excluded from these analyses. Based on descriptive analyses, bacterial AMR levels were consistently higher in dairy-type cattle as compared to beef-type cattle (Figure 2). Therefore, cattle type was used as an explanatory variable in logistic regression analyses in which the whole sampled population was considered to investigate associations between cattle type and AMR. The R package *lme4* (v.1.1-23) was used to fit mixed effects models (RStudio v.4.0.2). Continuous variables (temperature and body weight) were scaled as it was found to improve model convergence. When models were fitted with no random effects because the variance of the RE was equal to zero, the *glm* function (package *stats* v.4.0.2) was used with logit link. A backward elimination (40) approach was used and if the Akaike information criterion (AIC) value increased substantially and/ or the β-coefficient of a variable varied more than 30% upon removal, then it was retained in the model (28). Collinearity was evaluated through the fitted model through the variance inflation factor (VIF) (*car* package v.3.0-7; R Studio v.3.6.3). The possibility of a non-linear relationship between the continuous explanatory variables (weight and temperature) and the probability of the outcome on the log odds (logit) scale was tested by the addition of a quadratic term that was included in the model if statistically significant.

**Table 2:** Risk factors and outcomes investigated by multivariable logistic regression for beef and dairy-type cattle upon feedlot arrival.

	**Outcome**			
**Risk factor**	**Isolation of BRD-related bacteria^**a**^**	**Isolation of AMR BRD bacteria^**b**^**	**BRD-related morbidity**	**BRD-related mortality**
Country (Canada, US)	•	•	•	•
Sampling year (1st, 2nd)	•	•	•	•
Monthly interval^c^	•	•	•	•
Source (A, B, RD)^d^	•	•	•	•
BRD risk category^e^ (high, low)	•	•	•	•
Weight (kg)	•	•	•	•
Sex (female, male)^f^	•	•	•	•
Age class (calf, yearling)^g^	•	•	•	•
Weather^h^	•	•	NA^j^	NA^j^
Co-isolation of other BRD-related bacteria	•	NA	NA	NA
Isolation of BRD-related bacteria	NA	NA	•	•
*Mannheimia haemolytica* serotype (A1+A6 vs. A2)^i^	NA	•	NA	NA
At once feedlot capacity (>10 K, <10 K)	NA	NA	•	•
Suffered a previous BRD episode	NA	NA	NA	•

a *One model per bacterial species i.e., Mannheimia haemolytica, Pasteurella multocida, Histophilus somni, and Mycoplasma bovis*.

b *One model per bacterial species (i.e., Mannheimia haemolytica, Pasteurella multocida, and Histophilus somni)/antimicrobial combination*.

c *Sampling months were grouped from Aug-Nov, Dec-Feb, and Mar-May as an approximation of the seasons of the year*.

d *Auction (A) calves were predominant among beef-type cattle when compared to backgrounding operations (B) and ranch direct (RD) calves. In those AMR models in which source was a significant explanatory variable and its SE and β-coefficients were substantially high because of a low number of samples within 1 or more stratus, backgrounding operations and ranch direct samples were grouped and modeled against auction cattle. Likewise, auction samples were eliminated from those dairy AMR models in which <5 auction cattle samples were observed*.

e *Risk of suffering a BRD episode during the feeding period*.

f *Bulls accounted for a very small proportion of male population i.e., 2.2%*.

g *Calf, <1-year-old; yearling, over 1-year old*.

h *Weather conditions at feedlot entry included ambient temperature (°C), precipitation - yes (light and heavy rain/snow) or no (none, foggy), and wind speed - high (>20 km/h) or low (<20 km/h)*.

i *Due to the uneven distribution of the samples across serotype levels, samples with unknown M. haemolytica serotype isolates were removed from the analysis (n = 3 among beef-type cattle and n = 19 among dairy-type cattle) and serotype A1+A6 isolates were grouped separately from commensal A2 isolates due to their well-documented role in BRD*.

j *Weather-related risk factors were not included in morbidity and mortality analysis since the published literature does not provide evidence of their relevance on this matter (41). Additionally, weather measures were only related to the day that cattle arrived at the feedlots whereas the reported morbidity and mortality took place during the first 120 days on feed*.

*BRD, bovine respiratory disease; NA, not applicable*.

Clinical and Laboratory Standards Institute breakpoints for BRD bacteria have not been developed for all antimicrobials. Consequently, heatmaps with dendrograms were generated to visually explore the associations of MIC across all antimicrobials, except for trimethroprim/ sulfamethoxazole (SXT) and sulfadimethoxine which were tested at a single concentration. For these, antimicrobial MIC frequency distributions were plotted using the *heatmap.2* function from the *gplots* package v.3.0.4 in RStudio. Dendrograms were generated to explore associations across antimicrobials and across the strata of cattle type, monthly intervals, country of origin, source, feedlot, weight range (100 kg intervals: 100–199, 200–299, 300–399, 400–499, and 500–599 kg), age class, risk category, atmospheric temperature range (14°C intervals: −27 to −14, −13 to 0, 1 to 13, and 13 to 27°C), and BRD treatment (yes/no) and BRD mortality (yes/no). Feedlot was used as a clustering factor to explore whether any of the feedlots included in this study purchased cattle from locations containing higher levels of AMR. When non-clonal bacterial populations presenting high MICs were associated with a specific feedlot, individual dendrograms were generated for bacterial species/ feedlot combination using source, arrived from within a truck load, and truck load as clustering factors. Since not all the antimicrobials were tested in the same concentration range and most MIC frequency distributions were not normally distributed, MIC observed values were normalized (the minimum value of the dataset was subtracted to each value and divided by the maximum value in the dataset) bringing the data to the 0–1 scale (42).

## Results

### Sampling

A total of 2,824 DNPS were collected with 1,391 (49.3%) in year 1 and 1,433 (50.7%) in year 2 (Supplementary Table 2). Six feedlots provided DNPS only from beef-type cattle (B, D, E, F, H, I), two only from dairy-type cattle (C, J), with a mixture of both cattle types from the remainder (A, G). Four feedlots provided over 300 DNPS, five provided over 200 DNPS, and one feedlot provided <200 DNPS during the study (Supplementary Table 2). To optimize sampling numbers related to seasonal inconsistency in cattle availability, more than 10 animals may have been sampled from each truck load. Additionally, a small proportion of the collected DNPS (*n* = 241) failed to meet the quality standards for processing and were eliminated from the study. For beef-type cattle, the range of animals sampled per truck load was 3–57 (median = 10) whereas for dairy-type cattle, the number of animals sampled per load ranged from 9 to 20 (median = 10).

**Figure 3:**
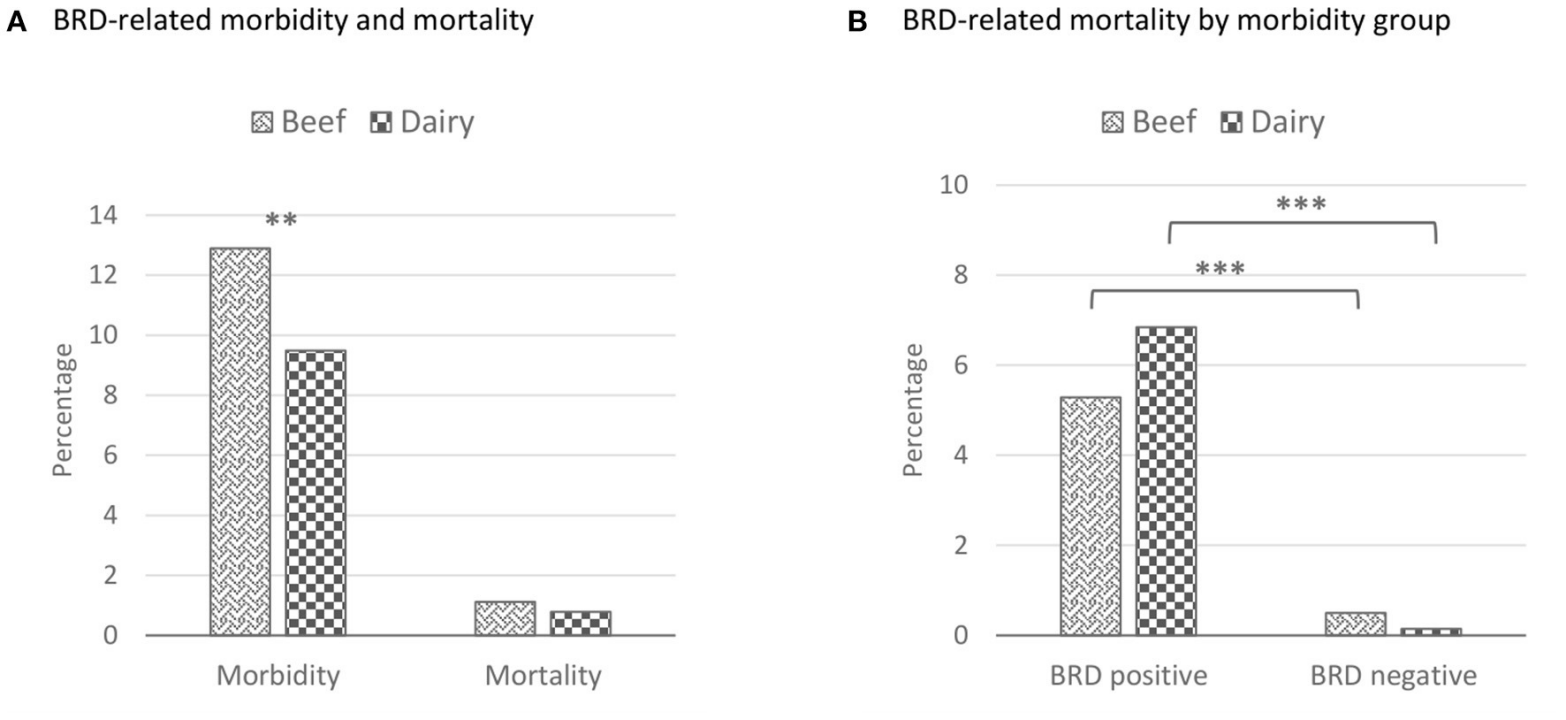
Proportion of cattle treated at least once for BRD and/ or succumbed to BRD during the first 120 d of the feeding period for beef and dairy-type cattle. These percentages represent unadjusted proportions. The asterisks represent the statistical test significance level as follows: “**” 0.01, “***” 0.001. BRD, bovine respiratory disease; Tx, treatment. **(A)** Beef morbidity proportion: 265/2,055 DNPS; beef mortality proportion: 23/2,055 DNPS; dairy morbidity proportion: 73/769 DNPS; dairy mortality proportion: 6/769 DNPS. Morbidity *X*^2^ (1, *n* = 2,428) = 6.15, *p* = 0.0132; mortality *X*^2^ (1, *n* = 2,428) = 0.63, *p* = 0.4264. **(B)** BRD-treated beef mortality proportion: 14/265; non-BRD treated beef mortality proportion: 9/1,790; BRD-treated dairy mortality proportion: 5/73; non-BRD treated dairy mortality proportion: 1/696. Beef *X*^2^ (1, *n* = 2,055) = 48.04, *p* < 0.001; dairy *Fisher Exact* (1, *n* = 769) = 35.27, *p* < 0.001.

Two thousand and fifty-five (72.8%) DNPS were collected from beef-type cattle and 769 (27.2%) DNPS were collected from dairy-type cattle. Compared to dairy-type cattle, beef cattle-type were heavier in incoming body weight, older upon arrival, and originated more frequently from within Canada (96.1%) (Table 3). Beef cattle-type were also more often classified as low BRD risk (60.5%) than dairy-type cattle (24.5%). Beef-type cattle were primarily sourced from auction barns (67.7%), whereas, a greater proportion of dairy-type cattle originated directly from dairy farms (88.4%). Of 2,824 cattle, 338 (12.0%) were clinically diagnosed with BRD over the 120 d feeding period (Figure 3) and 29 animals (5.6%) succumbed to BRD. Among the 29 mortalities, 19 (65.5%) were previously treated for BRD at least once.

**Table 3:** Feedlot cattle demographics from beef and dairy-type cattle upon feedlot arrival.

**Variable**		**Beef, *n* = 2,055**	**Dairy, *n* = 769**
Weight (kg)	Median	333	159
	Range	115–683	97–542
Age category	Calf	57%	92.3%
	Yearling	43%	7.7%
Country	Canada	96.1%	27.4%
	US	3.9%	72.6%
BRD risk	Low	60.5%	24.5%
Category	High	39.5%	75.5%
Source	Auction market	67.7%	1.3%
	Backgrounding operation	13.7%	10.3%
	Ranch direct	18.5%	88.4%

*These percentages represent unadjusted proportions*.

### Bacterial Isolation and Species Identification

**Figure 4:**
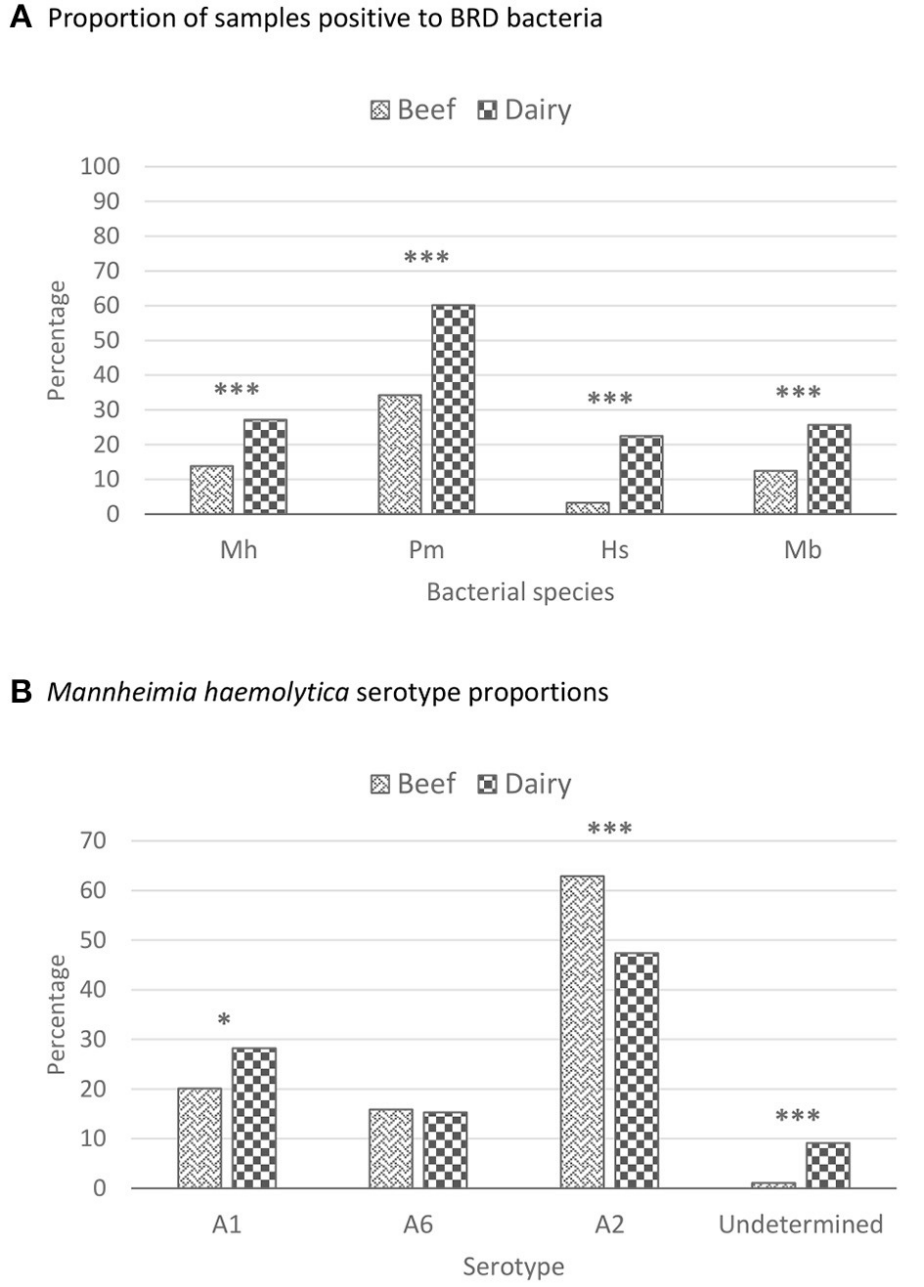
Proportion of deep nasopharyngeal swabs that were positive for BRD-related bacteria and *Mannheimia haemolytica* serotype proportions recovered from beef and dairy-type cattle upon feedlot arrival. These percentages represent unadjusted proportions. The asterisks represent the statistical test significance levels as follows: “*” 0.05, “***” 0.001. BRD, bovine respiratory disease; Hs, *Histophilus somni*; Mb, *Mycoplasma bovis*; Mh, *Mannheimia haemolytica*; Pm, *Pasteurella multocida*. **(A)** Beef Mh positive proportion: 283/2,055; beef Pm positive proportion: 703/2,055; beef Hs positive proportion: 68/2,055; beef Mb positive proportion: 257/2,055; dairy Mh positive proportion: 209/769; dairy Pm positive proportion: 463/769; dairy Hs positive proportion: 173/769; dairy Mb positive proportion: 198/769. Mh *X*^2^ (1, *n* = 2,824) = 69.91, *p* < 0.001; Pm *X*^2^ (1, *n* = 2,824) = 156.04, *p* < 0.001; Hs *X*^2^ (1, *n* = 2,824) = 263.94, *p* < 0.001; Mb *X*^2^ (1, *n* = 2,824) = 72.60, *p* < 0.001. **(B)** Beef Mh A1 positive proportion: 57/283; beef Mh A6 positive proportion: 45/283; beef Mh A2 positive proportion: 178/283; beef Mh unknown positive proportion: 3/283; dairy Mh A1 positive proportion: 59/209; dairy Mh A6 positive proportion: 32/209; dairy Mh A2 positive proportion: 99/209; dairy Mh unknown positive proportion: 19/209. Mh A1 *X*^2^ (1, *n* = 492) = 4.36, *p* = 0.0367; Mh A6 *X*^2^ (1, *n* = 492) = 0.03, *p* = 0.8587; Mh A2 *X*^2^ (1, *n* = 492) = 11.78, *p* < 0.001; Mh unknown *Fisher Exact* (1, *n* = 492) = 18.15, *p* < 0.001.

A total of 1,646 out of 2,824 (58.3%) DNPS were positive for at least one of the bacterial species. Overall, *P. multocida* (41.3%) was most commonly isolated, followed by *M. haemolytica* (17.4%), *M. bovis* (16.1%), and *H. somni* (8.5%) (Figure 4A). A total of 492 *M. haemolytica* isolates were obtained from beef and dairy-type cattle (Figure 4B). For beef-type cattle, 57 isolates (20.1%) were serotype A1, 178 (62.9%) were A2, 45 (15.9%) were A6, and 3 (1.1%) were undetermined. In dairy-type cattle, 59 (28.2%), 99 (47.4%), 32 (15.3%), and 19 (9.1%) of the isolates were serotype A1, A2, A6, and undetermined, respectively.

### Antimicrobial Susceptibility Testing

Overall, the proportions of AMR-resistant *M. haemolytica, P. multocida*, and *H. somni* isolates recovered from dairy-type cattle were higher than those from beef-type cattle (Figures 2A–D). *Mannheimia haemolytica* isolates from dairy-type cattle had higher MIC_90_ for CTET and neomycin (NEO) (8 and >32 μg/mL, respectively) than those from beef-type cattle (2 and 8 μg/mL, respectively) (Supplementary Table 1.5). Likewise, *P. multocida* isolates from dairy-type cattle had higher MIC_50_/MIC_90_ values for CTET (4/8 μg/mL), NEO (>32/>32 μg/mL), and TYLT (>32/>32 μg/mL) than isolates from beef-type cattle (0.5/1, 16/16, and 16/32 μg/mL, respectively). Among *H. somni* isolates from dairy-type cattle, MIC_50_/MIC_90_ values were also higher for CTET (2/4 μg/mL) and TYLT (8/16 μg/mL) than those from beef-type cattle (0.25/0.25 and 4/4 μg/mL, respectively).

Antimicrobial susceptibility testing was completed for 490 out of 492 (99.6%) of the *M. haemolytica* isolates (281 from beef and 209 from dairy) (Supplementary Table 1.1). The most frequently observed resistances were OXY (10%), TIL (6.4%), and ampicillin (AMP; 4.6%) among isolates from beef-type cattle and OXY (46.4%), TIL (26.8%), and TUL (21.0%) among dairy-type cattle isolates (Figure 2A, Supplementary Figure 1.1). Interestingly, all *M. haemolytica* isolates exhibited a high MIC for TYLT at 32 μg/mL (1.8% of the isolates) and >32 μg/mL (98.2% of the isolates). A higher proportion of *M. haemolytica* serotype A1 isolates were resistant to ENRO (7.8%), DANO (24.1%), and OXY (61.2%) than serotype A2 (0, 1.5, and 18.5%) and A6 (0, 1.3, and 1.3%) isolates. In contrast, serotype A2 isolates exhibited higher proportion of resistance to penicillin (PEN; 7.6%), TUL (17.1%), and FFN (13.5%) than did A1 and A6 (Supplementary Figure 1.2). Resistance to SPE and TIL was similar between A1 and A2 isolates (1.7 and 0.4%, respectively; 19.8 and 18.5%, respectively). Of the three serotypes, A6 showed the overall lowest AMR. Among *P. multocida* isolates, resistance to OXY (8.4%), SPE (8.1%), and AMP (7.7%) was most frequent from beef compared to OXY (89.7%), TIL (73.1%), and DANO (55.8%) from dairy-type cattle (Figure 2B). *Pasteurella multocida* also presented high MICs (MIC_50_ and MIC_90_ > 32 μg/mL) for TYLT, especially in isolates from dairy-type cattle, with only one (0.2%) *P. multocida* isolate exhibiting resistance to ceftiofur (TIO; cephalosporin). *Histophilus somni* isolates from dairy-type cattle were more frequently resistant to OXY (70.5%), SPE (22%), and PEN (11.0%) compared to isolates from beef-type cattle, which only exhibited resistance to AMP (2.9%) (Figure 2C). The total percentage of MDR in all *M. haemolytica, P. multocida*, and *Histophilus somni* isolates were 12, 34.2, and 1.25%, respectively (Figure 2D).

*Mycoplasma bovis* MIC_50_/MIC_90_ values were consistently higher for macrolides than for other antimicrobials, regardless of cattle type, especially for TIL (>256 μg/mL both) and TIP (>128 μg/mL both) (Supplementary Table 1.5). Likewise, the MIC_50_/MIC_90_ values of GAM (128/>256 μg/mL), TUL (16/>256 μg/mL), and TYLT (32/>128 μg/mL) were also high. Enrofloxacin (dairy-type cattle isolates), GAM (beef and dairy isolates), TUL (beef and dairy isolates), and TYLT (beef and dairy isolates) MIC distributions exhibited a bimodal pattern. As previously reported, AlamarBlue™ prevented the estimation of the OXY MIC of 2 *M. bovis* isolates from dairy-type cattle when MIC ≥ 32 μg/mL (11).

### Antimicrobial Resistance Genetic Determinants

Overall, the AMR phenotypes for *M. haemolytica, P. multocida*, and *H. somni* were corroborated with the presence of related antimicrobial resistance genes (ARG), as inferred from the WGS data (Supplementary Tables 3.1–3.3). The *tet*(H) gene was the most abundant determinant identified and corresponded with OXY resistance in *M. haemolytica* and *P. multocida* (Supplementary Tables 3.1, 3.2). In *H. somni, tet(H)* was detected in isolates with CTET MICs ranging between 1 and >4 μg/mL (Supplementary Table 3.3). Genes related to NEO and kanamycin resistance were the second most abundant determinants identified [*aph(3')-Ia*], coinciding with NEO MIC values > 32 μg/mL for *M. haemolytica* and *P. multocida*. Considering that the NEO concentrations were not tested beyond 16 μL/mL for *H. somni*, it was not possible to determine if the presence of *aph(3')-Ia* conferred NEO MIC > 32 μg/mL. Likewise, the ARG for GEN resistance [*ant(2”)-Ia*] was present in *M. haemolytica* when MIC values for this drug were >16 μg/mL, whereas in *H. somni, aac(3)-Iva*, a gentamycin (GEN) resistance gene, was associated with the GEN phenotype in only 5 of the 8 GEN resistant (MIC > 8 μg/mL) isolates. The presence of *flo*R corresponded with FFN MIC ≥ 8 μg/mL for *M. haemolytica*, MIC ≥ 4 μg/mL for *P. multocida*, and MIC ≥ 0.5 μg/mL for *H. somni*. The presence of *bla*_ROB−1_ was associated with AMP resistance and the detection of *aadA*25 (*M. haemolytica*) or *aadA*31 (*P. multocida and H. somni*) was associated with SPE resistance. In *M. haemolytica* and *P. multocida*, the presence of mutations in the quinolone resistance-determining region (QRDR) was related to resistance to FQs. In many cases, resistance to macrolides (TIL, TUL) was related to the presence of the *erm*(42) and/or the *mph*(E)-*msr*(E) operon in *M. haemolytica*, and *erm*(42) and/or the *mph*(E)-*msr*(E) operon or A2058G mutation in the 23S rRNA gene of *P. multocida*. No corroboration could be established between macrolide phenotype and genotype for *H. somni* isolates as 5 of the 24 sequenced isolates carried *erm*(42) and/or *erm*(F) genes, yet they were not resistant to macrolides (TIL and TUL MIC ranging from 2 to 4 μg/mL). Streptomycin resistance ARGs *aph(3”)-Ib* and *aph(6)-Id* frequently co-existed with NEO determinant *aph(3')-Ia* in the three *Pasteurellaceae* species. However, phenotypic streptomycin resistance was not assessed in any of the isolates.

### Multivariable Logistic Regression

#### Recovery of BRD-Related Bacteria

Multivariable logistic regression determined that the odds of isolating a BRD pathogens was variably associated with the isolation of other BRD pathogens and the monthly arrival interval (Table 4). In the second year, the odds ratio of recovering *M. bovis* from both cattle types was higher than in the first year i.e., 2.3 (95% CI = 1.3–4.0, *p* = 0.006) and 2.5 (95% CI = 1.3–4.74, *p* = 0.004) in both beef and dairy-type cattle, respectively (Supplementary Tables 4.1, 4.2).

**Table 4:** Significant results obtained from logistic regression of recovery of bovine respiratory disease complex bacteria from beef and dairy-type cattle upon feedlot arrival.

**Risk factor**	***M. haemolytica***			***P. multocida***			***H. somni***			***M. bovis***		
	**OR**	**95% CI**	***p*-value**	**OR**	**95% CI**	***p*-value**	**OR**	**95% CI**	***p*-value**	**OR**	**95% CI**	***p*-value**
**Monthly intervals – BEEF**												
Aug–Nov	1	1	–	1	1	–	1	1	–	1	1	–
Dec–Feb	2.4	1.7–3.6	**<0.001**	1.45	1.0–2.1	**0.048**	1.7	0.7–4.1	0.248	12.7	5.4–30.1	**<0.001**
Mar–May	1.2	0.8–1.8	0.279	0.93	0.6–1.4	0.700	3.5	1.4–8.5	**0.005**	14.5	6.1–34.9	**<0.001**
**Monthly intervals - DAIRY**												
Aug–Nov	1	1	–	1	1	–	1	1	–	1	1	–
Dec–Feb	1.3	0.8–2.2	0.266	1.1	0.6–2.0	0.716	na	na	na	na	na	na
Mar–May	2.4	1.5–3.9	**<0.001**	1.8	1.1–3.2	**0.034**	na	na	na	na	na	na
**Isolation of other BRD-related bacteria - BEEF**												
Mh	ni	ni	ni	na	na	na	2.2	1.2–4.0	**0.011**	1.5	0.9–2.3	**0.060**
Pm	na	na	na	ni	ni	ni	na	na	na	1.6	1.2–2.2	**0.006**
Hs	1.9	1.1–3.6	**0.025**	na	na	na	ni	ni	ni	na	na	na
Mb	1.6	1.1–2.4	**0.014**	1.6	1.1–2.2	**0.005**	na	na	na	ni	ni	ni
**Isolation of other BRD-related bacteria - DAIRY**												
Mh	ni	ni	ni	na	na	na	0.6	0.4–1.0	**0.048**	na	na	na
Pm	na	na	na	ni	ni	ni	na	na	na	1.5	1.0–2.2	**0.050**
Hs	0.7	0.4–1.0	**0.063**	na	na	na	ni	ni	ni	na	na	na
Mb	na	na	na	1.5	1.0–2.3	**0.038**	na	na	na	ni	ni	ni

*CI, confident interval; Hs, Histophilus somni; Mb, Mycoplasma bovis; Mh, Mannheimia haemolytica; na, not applicable; ni, not included; OR, odds ratio; Pm, Pasteurella multocida*.

*na, that variable was not significant at the p-value ≤ 0.1 level and was eliminated from the regression*.

*ni, not included as an explanatory variable in the model*.

*Bold p-values indicate statistically significant results at p ≤ 0.1*.

#### Recovery of AMR Bacteria

Depending on the BRD bacterial species isolated from beef-type cattle, the odds of these bacteria being AMR were between 8.5 and 17.5 higher between Mar–May as compared to Aug–Nov (Tables 5, 6). In dairy-type cattle, BRD-related bacteria had lower odds of being AMR during Mar–May than Aug–Nov, and Aug–Nov as compared to Dec–Feb (Tables 7, 8). The country of origin did not affect the odds of isolating AMR BRD bacteria in beef-type cattle (Supplementary Table 4.1), whereas in dairy-type cattle it varied depending on the antimicrobial and the bacterial species (Tables 7–9). The odds of recovering AMR bacteria from backgrounded beef-type feedlot cattle were higher than for those purchased at auction (Tables 5, 6). The BRD risk category showed no effect on AMR among beef-type cattle (Supplementary Table 4.3), whereas for dairy-type cattle, it varied depending on the antimicrobial and the bacterial species (Supplementary Table 4.4). Overall, a higher ambient temperature was associated with greater odds of recovering resistant bacteria in both cattle types (Supplementary Tables 4.3, 4.4). *Mannheimia haemolytica* A1 and A6 serotypes were associated with higher odds of OXY resistance in beef (OR = 38.3, 95% CI = 4.8–304.1, *p* < 0.001) and dairy-type cattle (OR = 4.8, 95% CI = 1.9–11.9, *p* < 0.001) as compared to A2. The A1 and A6 serotypes, presented lower odds of being resistant to almost all antimicrobials tested among dairy-type cattle when compared to A2 (Tables 5, 6). Dairy-type cattle were associated with higher levels of AMR as compared to beef-type cattle (Supplementary Tables 4.3, 4.4).

**Table 5:** Significant results obtained from logistic regression of antimicrobial resistant *Mannheimia haemolytica* from beef-type cattle upon feedlot arrival.

**Risk factor**	**Ampicillin**			**Oxytetracycline**			**Tilmicosin**			**Tulathromycin**		
	**OR**	**95% CI**	***p*-value**	**OR**	**95% CI**	***p*-value**	**OR**	**95% CI**	***p*-value**	**OR**	**95% CI**	***p*-value**
**Monthly intervals**															
Aug–Nov	1	1	–	1	1	–	1	1	–	1	1	–
Dec–Feb	0.9	0.2–5.0	0.879	0.6	0.04–7.7	0.689	1.0	0.1–11.0	0.973	na	na	na
Mar–May	10.0	1.3–76.8	**0.027**	17.5	1.1–275.9	**0.041**	11.6	1.1–121.2	**0.040**	na	na	na
**Source**															
A	1	1	–	1	1	–	1	1	–	1	1	–
B	na	na	na	34.0	1.5–776.4	**0.027**	11.7	1.4–100.9	**0.024**	85.8	4.3–1,715.4	**0.003**
RD	na	na	na	2.6	0.2–34.6	0.433	4.0	0.7–23.6	0.121			
***Mannheimia haemolytica*** **serotype**															
A2	1	1	–	1	1	–	1	1	–	1	1	–
A1 + A6	na	na	na	38.3	4.8–304.1	**<0.001**	6.6	1.5–29.9	**0.014**	na	na	na

*A, auction; B, backgrounding operations; CA, Canada; CI, confident interval; OR, odds ratio; RD, ranch direct; RF, risk factor*.

*na, that variable was not significant at the p-value ≤ 0.1 level and was eliminated from the regression*.

*Bold p-values indicate statistically significant results at p ≤ 0.1*.

**Table 6:** Significant results obtained from logistic regression of antimicrobial resistant *Pasteurella multocida* from beef-type cattle upon feedlot arrival.

**Risk factor**	**Ampicillin**			**Oxytetracycline**			**Spectinomycin**			**Tilmicosin**			**Tulathromycin**		
	**OR**	**95% CI**	***p*-value**	**OR**	**95% CI**	***p*-value**	**OR**	**95% CI**	***p*-value**	**OR**	**95% CI**	***p*-value**	**OR**	**95% CI**	***p*-value**
**Monthly interval**																					
Aug–Nov	1	1	–	1	1	–	1	1	–	1	1	–	1	1	–
Dec–Feb	na	na	na	na	na	na	1.3	0.1–12.5	0.834	na	na	na	na	na	na
Mar–May	na	na	na	na	na	na	8.5	1.1–65.4	**0.040**	na	na	na	na	na	na
**Source**																					
A	1	1	–	1	1	–	1	1	–	1	1	–	1	1	–
B	na	na	na	13.2	0.002–0.1	**0.018**	13.4	1.6–113.8	**0.017**	21.9	0.7–698.5	**0.080**	35.9	1.0–1249.4	**0.048**
RD	na	na	na	1.3	0.2–10.4	0.807	1.8	0.3–11.4	0.545						

*A, auction; B, backgrounding operations; CI, confident interval; OR, odds ratio; RD, ranch direct*.

*na, that variable was not significant at the p-value ≤ 0.1 level and was eliminated from the regression*.

*Bold p-values indicate statistically significant results at p ≤ 0.1*.

**Table 7:** Significant results obtained from logistic regression of antimicrobial resistant *Mannheimia haemolytica* from dairy-type cattle upon feedlot arrival.

**Risk factor**	**Ampicillin**			**Danofloxacin**			**Florfenicol**			**Oxytetracycline**			**Penicillin**			**Tilmicosin**			**Tulathromycin**		
	**OR**	**95% CI**	***p*-value**	**OR**	**95% CI**	***p*-value**	**OR**	**95% CI**	***p*-value**	**OR**	**95% CI**	***p*-value**	**OR**	**95% CI**	***p*-value**	**OR**	**95% CI**	***p*-value**	**OR**	**95% CI**	***p*-value**
**Country**																					
CA	1	1	–	1	1	–	1	1	–	1	1	–	1	1	–	1	1	–	1	1	–
US	na	na	na	0.02	0.002–0.2	**0.001**	18.7	3.3–251.8	**0.004**	na	na	na	na	na	na	na	na	na	na	na	na
**Monthly intervals**																					
Aug–Nov	1	1	–	1	1	–	1	1	–	1	1	–	1	1	–	1	1	–	1	1	–
Dec–Feb	1.0	0.2–6.0	0.964	8.1	1.3–50.7	**0.025**	9.5	1.6–68.3	**0.017**	3.33	0.9–13.0	**0.082**	0.9	0.2–3.3	0.831	3.3	0.9–12.4	**0.077**	0.3	0.02–4.7	0.426
Mar–May	0.2	0.03–1.1	**0.057**	1.1	0.3–3.5	0.900	1.9	0.4–10.8	0.465	0.95	0.3–2.8	0.930	0.2	0.03–0.9	**0.031**	0.2	0.05–0.9	**0.041**	0.1	0.01–0.9	**0.049**
***Mannheimia haemolytica*** **serotype**																					
A2	1	1	–	1	1	–	1	1	–	1	1	–	1	1	–	1	1	–	1	1	–
A1 + A6	0.5	0.1–1.8	0.259	12.1	3.4–43.3	**<0.001**	0.02	0.002–0.1	**<0.001**	4.8	1.9–11.9	**<0.001**	0.1	0.01–0.4	**0.004**	0.1	0.05–0.4	**<0.001**	0.03	0.01–0.2	**<0.001**

*CA, Canada; CI, confident interval; OR, odds ratio; RF, risk factor*.

*na, that variable was not significant at the p-value ≤ 0.1 level and was eliminated from the regression*.

*Bold p-values indicate statistically significant results at p ≤ 0.1*.

**Table 8:** Significant results obtained from logistic regression of antimicrobial resistant *Pasteurella multocida* from dairy-type cattle upon feedlot arrival.

**Risk factor**	**Ampicillin**			**Danofloxacin**			**Enrofloxacin**			**Florfenicol**			**Oxytetracycline**			**Tilmicosin**			**Tulathromycin**		
	**OR**	**95% CI**	***p*-value**	**OR**	**95% CI**	***p*-value**	**OR**	**95% CI**	***p*-value**	**OR**	**95% CI**	***p*-value**	**OR**	**95% CI**	***p*-value**	**OR**	**95% CI**	***p*-value**	**OR**	**95% CI**	***p*-value**
**Country**																					
CA	1	1	–	1	1	–	1	1	–	1	1	–	1	1	–	1	1	–	1	1	–
US	na	na	na	0.003	0.0003–0.04	**<0.001**	0.01	0.001–0.1	**<0.001**	0.09	0.02–0.4	**0.002**	na	na	na	na	na	na	0.01	0.001–0.1	**<0.001**
**Monthly intervals**																					
Aug–Nov	1	1	–	1	1	–	1	1	–	1	1	–	1	1	–	1	1	–	1	1	–
Dec–Feb	1.53	0.3–6.8	0.568	na	na	na	0.7	0.2–2.3	0.588	2.5	0.9–6.1	**0.053**	na	na	na	na	na	na	0.4	0.1–1.1	**0.063**
Mar–May	0.13	0.02–0.9	**0.037**	na	na	na	0.1	0.04–0.4	**<0.001**	0.3	0.1–0.9	**0.026**	na	na	na	na	na	na	1.3	0.5–3.2	0.647

*CA, Canada; CI, confident interval; OR, odds ratio*.

*na, that variable was not significant at the p-value ≤ 0.1 level and was eliminated from the regression*.

*Bold p-values indicate statistically significant results at p ≤ 0.1*.

**Table 9:** Significant results obtained from logistic regression of antimicrobial resistant *Histophilus somni* from dairy-type cattle upon feedlot arrival.

**Risk factor**	**Ampicillin**			**Oxytetracycline**			**Penicillin**			**Spectinomycin**		
	**OR**	**95% CI**	***p*-value**	**OR**	**95% CI**	***p*-value**	**OR**	**95% CI**	***p*-value**	**OR**	**95% CI**	***p*-value**
**Country**												
CA	1	1	–	1	1	–	1	1	–	1	1	–
US	18.4	3.7–92.1	**<0.001**	na	na	na	23.22	3.6–148.9	**<0.001**	na	na	na

*CA, Canada; CI, confident interval; OR, odds ratio*.

*na, that variable was not significant at the p-value ≤ 0.1 level and was eliminated from the regression*.

*Bold p-values indicate statistically significant results at p ≤ 0.1*.

#### BRD-Related Morbidity and Mortality

There were no differences in the morbidity or mortality between cattle types sourced in either Canada or the US (Supplementary Table 4). The isolation of any BRD-related nasal bacteria upon feedlot arrival was not related to BRD mortalities during the feeding period. Cattle that were treated for BRD at least once were positively associated with dying from BRD. Additionally, different risk factors were associated with morbidity and/or mortality depending on cattle type i.e., BRD risk, monthly interval, weight, and age (Supplementary Tables 4.6–4.9).

### Heatmaps

Antimicrobial resistant profiles of isolates from dairy-type cattle presented higher MIC values (values closer to 1), including for antimicrobials with no SIR categories such as CTET or TYLT, in *M. haemolytica, P. multocida*, and *H. somni* (Supplementary Figures 1.3.1–1.3.3). *Mycoplasma bovis* isolates with high macrolide MIC values were obtained from both cattle types. However, higher ENRO MICs were mainly associated with isolates from dairy-type calves as indicated by MIC_50_/MIC_90_ values (Supplementary Table 1.5, Supplementary Figure 1.3.4). Additionally, *M. bovis* isolates obtained from backgrounding beef-type cattle predominated among AMR profiles showing higher MICs as compared to auction and ranch direct beef-type cattle (Supplementary Figure 1.3.41). Interestingly, *M. haemolytica* serotypes A1 and A6 isolated from beef-type cattle were mainly distributed among isolates showing higher MICs, whereas A2 typically exhibited lower MICs (Supplementary Figure 1.3.13). *Pasteurella multocida* from US dairy-type cattle exhibited higher MIC values as compared to those isolated from Canadian dairy-type cattle (Supplementary Figure 1.3.17). Among dairy-type cattle, *M. haemolytica* and *M. bovis* isolates collected at feedlot C also had higher MICs than those from feedlots A, G, and J (Supplementary Figures 1.3.16, 1.3.49). Notably, feedlot C purchased dairy-type cattle mainly from different farms located in Alberta, Canada, whereas feedlots A, G, and J purchased dairy-type cattle mainly from the US (Supplementary Figures 1.3.50, 1.3.51).

## Discussion

In this study, we described the prevalence of AMR in *M. haemolytica, P. multocida*, and *H. somni* in cattle at feedlot arrival, and documented that it was higher in dairy than beef-type cattle. Multidrug-resistant *Pasteurellaceae* bacteria have been isolated from BRD clinical cases and mortalities before (43–45), and AMR has been investigated in cattle at feedlot arrival in previous studies (14–16, 46). However, to our knowledge this study is the first in North America to collect all four species of the BRD bacterial complex from a broad cross-sectional feedlot cattle population, and investigate their antimicrobial susceptibilities at feedlot entry. One limitation of this study is the large ORs and wide CIs generated by logistic regression in some instances (Supplementary Tables 4.1–4.9) limited the precision of the conclusions obtained in this study.

The prevalence of *P. multocida* and *H. somni* in beef-type cattle was higher compared to previous studies that collected respiratory samples from feedlot cattle at arrival, but comparable for *M. haemolytica* (14–16). The higher prevalence of *M. haemolytica* serotype A2 was expected as compared to A1 and A6, as it is more frequently isolated from healthy cattle (47). Dairy-type cattle tended to harbor more members of the BRD bacterial complex than beef-type cattle. This association may be a consequence of early weaning practices in the dairy industry (17). Most dairy calves are weaned within a day of birth (17), which may limit their ingestion of colostrum. The acquisition of passive immunity at this age is limited during a critical time when the calf's immune system is still immature (48). Most beef calves are raised under extensive conditions, whereas dairy calves are often housed in confinement from birth, a practice that could promote the transmission of BRD bacteria among individuals. As reported by Griffin (49), the odds of isolating *Mycoplasma bovis* from both beef and dairy cattle classified as high-BRD risk were higher than those classified as low risk. However, in the current study, *M. haemolytica, P. multocida*, and *H. somni* were not associated with cattle classified as high-BRD risk.

The proportion of cattle that had BRD at least once (12%) and succumbed to it (5.6%) was comparable to those previously reported, 16.2 and 4%, respectively (50). It has been suggested that sourcing cattle directly from ranches reduces exposure to BRD-related bacteria (49), but we did not find greater odds of recovering BRD bacteria from beef-type cattle purchased at auction as compared to those from backgrounding operations or directly from ranches. Nevertheless, auction-sourced cattle did have greater odds of being treated for BRD. Auction market-derived beef-type cattle (*n* = 1,392) originated from 69 different locations. Frequent comingling of cattle from different locations can increase the incidence of BRD morbidity during the feeding period (51, 52). Most weaned beef calves enter the feedlot in the fall and at this time a large number of calves are comingled during sale and shipped to feedlots. During this time they are subject to handling and transport stress, resulting in an increase in BRD-morbidities and mortalities (41). Although the fall interval (Aug-Nov) was associated with more BRD morbidity in beef-type cattle, it was not associated with higher isolation of BRD bacteria at arrival. Heavier and older (yearlings) cattle were less likely to develop BRD, an observation that agrees with that of others (41). High-BRD risk beef-type cattle were not associated with greater BRD during the feeding period. It has been reported that high-BRD risk cattle are over 100 times more likely to be administrated metaphylactic macrolides than low-BRD risk cattle (53). It is well-established that the use of metaphylactic antimicrobials in moderate to high risk BRD cattle reduces morbidity and mortality (53) which could account for the lower BRD morbidity in high-BRD risk cattle. Only the isolation of *P. multocida* in dairy-type cattle at feedlot entry was associated with higher BRD morbidity during the feeding period (Supplementary Table 4.8). The bovine respiratory tract microbiome undergoes substantial changes in bacterial composition after feedlot placement (54). Bovine respiratory disease is also a complex disease process involving viral co-infections, and host immunity and environmental factors. Therefore, there may not be a causal link between the presence/absence of BRD bacteria in the respiratory tract of cattle upon arrival and morbidity and mortality during the feeding period. This study was limited to considering only the implications of the bacteriological component on arrival for BRD morbidities and mortalities that occurred later in the feeding period. Other factors known to contribute to this disease complex, such as immune and nutritional status, vaccination, housing, management practices, and the respiratory virome (8, 55) were not evaluated.

A higher risk of BRD morbidity has been associated with the purchase of calves in the fall (41). Aug–Nov (for dairy) and Dec–Feb (for beef and dairy) as compared to Mar–May were associated with higher AMR possibly as a result of increased AMU during this time of the year. Higher odds of AMR nasal BRD bacteria recovery were observed in beef-type cattle backgrounded in feedlots as compared to auction-derived calves. This observation most likely reflects the administration of some antimicrobials to cattle at backgrounding feedlots and the confined conditions that may promote transmission of AMR bacteria amongst cattle. However, some auction-derived cattle may also have been in a backgrounding operation prior to transfer to an auction market but were classified as auction-derived due to the impossibility of gathering such information. The odds of isolating AMR-BRD bacteria were consistently higher in dairy-type cattle than beef-type cattle (Supplementary Table 4.5). In North America, dairy calves are typically housed indoors in confinement whereas most beef calves are on pasture until weaning. Dairy calves that are fed in feedlots for meat production are moved to calf grower operations as they come out of the hutch at 1 day of age (FHMS personal communication). In calf grower operations, dairy-type calves are housed in pens which may promote the transmission of AMR bacteria as per in beef-type cattle backgrounded in feedlots. It is also possible that these dairy calves were administrated more antimicrobials than beef-type calves of a similar age to assist their immature immune system to fight bacterial infections that may take place in the growing operation.

Low levels of AMR in *M. haemolytica* isolated from newly arrived feedlot cattle has been previously reported, with few studies investigating AMR in *P. multocida* and *H. somni. Mannheimia haemolytica* and *P. multocida* resistance to TIL and TUL were very low (up to 6.4%) in beef cattle compared to dairy cattle, most likely because macrolides are less frequently administrated to ranch beef calves than dairy calves (56–58). Even though the use of TYLT is less prevalent than TIL and TUL in ranch beef calves (58), *M. haemolytica* and *P. multocida* had high TYLT MICs, a phenomenon also observed in dairy calves. Previous surveillance studies have also reported high TYLT MICs in these two bacterial species in beef cattle at arrival and during the feeding period (6, 46). Further studies are required to determine if *M. haemolytica* and *P. multocida* exhibit some degree of intrinsic resistance to TYLT as a result of up-regulation of antimicrobial efflux systems (59) or impairment of cellular uptake as per AMGs (60). *Mannheimia haemolytica* obtained from cattle at feedlot entry exhibited no resistance to TUL a year after it was approved for the control of BRD in Canada (46). It is possible that the slightly higher resistance (i.e., from 0 to 3.6%) observed in the present study reflects the increased use of TUL, but more isolates should be collected over a longer period of time to confirm this trend.

Macrolide resistance phenotype (TIL and TUL) appeared to be due to the presence of *erm*(42) and/or the macrolide efflux protein and phosphotransferase gene pair *msr*(E)-*mph*(E) in *M. haemolytica* and *P. multocida* isolates. Three macrolide resistant *P. multocida* isolates possessed the A2058G mutation in 23S rRNA. Macrolide resistance due to rRNA mutations is well-documented in bacteria with a homogeneous or heterogeneous presence in *rrn* operons (61). In *H. somni, erm*(42) and/or *erm*(F) genes were present, but this did not appear to be linked to macrolide MICs. Furthermore, TYLT did not seem to be a reliable phenotypic indicator of macrolide resistance for *Pasteurellaceae* species isolated in this study due to high MIC values observed (Supplementary Tables 3.1, 3.2).

As previously reported, the resistance to TIO was extremely low for *P. multocida* (1/515, 0.2%) and absent in *M. haemolytica* (16, 47) and *H. somni*. These results probably reflect the limited use of TIO in beef cattle (58). Third generation cephalosporins are more frequently administrated to dairy cattle than to beef cattle (56, 57), yet resistance to this drug was not observed in BRD bacteria isolated from dairy calves. The overall resistance to β-lactam antimicrobials was relatively low in beef and dairy cattle, even though this drug class is commonly used in both (56–58). In our study, all *M. haemolytica* isolates that had AMP MICs > 16 μg/mL and all *H. somni* with MICs of 1–2 μg/mL harbored the *bla*_ROB−1_ gene. Furthermore, one *M. haemolytica* with MIC values of 0.25 μg/mL had *bla*_ROB−1_, and another isolate with similar MIC value possessed *bla*_OXA−2_. In *Pasteurellaceae* species, *bla*_ROB−1_ is typically plasmid-associated (62, 63).

Resistance to OXY was the highest resistance observed across *Pasteurellaceae* isolated from both cattle types (10 and 46.4% in beef and dairy cattle types, respectively), which likely reflects the frequent use of TETs (57, 58). Levels of TET-resistance in *Pasteurellaceae* are even high in feedlot cattle raised without the use of antimicrobials (45). Considering the importance of TETs for the treatment and prevention of histophilosis in western Canada (53), the high level of OXY resistance (70.5%) in *H. somni* isolates from dairy cattle is worrisome. Our results suggest that antimicrobials other than TETs would be more effective for the treatment of *H. somni* infections in feedlot dairy calves. The ARG *tet*(H) was always present in OXY-resistant isolates, a finding consistent with that of others (14, 45). Previous studies have identified *tet*(H) in AMR *M. haemolytica* from clinical BRD cases (64), and it is also frequently found in *P. multocida* (59) and *H. somni* (65).

High FQs resistance was observed in *M. haemolytica* and *P. multocida* isolates from dairy calves. Danofloxacin is not approved for use in lactating dairy cows (56, 57) but it is approved for dairy calves (66). Resistance to FQ was associated with mutations in the QRDR including DNA-gyrase encoded by the *gyrA* and *gyrB* genes and topoisomerase IV encoded by *parC* and *parE* (67–69).

Frequent administration of FFN has been reported in dairy but not in beef cattle (57, 58). Such antimicrobial usage may explain the higher FFN resistance observed in dairy *M. haemolytica* and *P. multocida* isolates, and higher MICs in *H. somni* isolates from dairy compared to beef cattle (Supplementary Figure 1.3.3). This resistance and/or high MIC corresponded with the presence of *floR* as inferred from sequenced isolates from these three species.

Aminocyclitols are infrequently used in beef or dairy cattle (56–58) yet, *H. somni* (dairy isolates) and *P. multocida* (beef and dairy isolates) exhibited 22, 8.1, and 19% resistance to SPE, respectively. Co-resistance with other antimicrobials that travel on the same MGE as SPE likely accounts for this resistant phenotype (45). Resistance to SPE was conferred by the *aadA31* gene as previously described (70). In accordance with previous studies (45, 59), linkage of AMG resistance genes *aph(3*″*)-Ib, aph(6)-Id, aph(3*′*)-Ia*, and sulfonamide resistance gene *sul2* was found in *M. haemolytica, P. multocida*, and *H. somni*. The GEN-resistance determinant *aac(3)-IVa* also co-occurred with aminoglycoside and sulfonamide resistance genes in *H. somni* depicting GEN MIC > 8 μg/mL. In the isolates harboring these groups of AMR determinants, *floR* and *erm*(42) were also frequently clustered with the AMG-associated ARGs and *sul2* genes. Co-existence of these ARGs along with *tet*(H) is a hallmark of ICE (45, 59).

*Mannheimia haemolytica* serotype A2 isolates from dairy-type cattle were more frequently associated with AMR than serotype A1 and A6 isolate from beef-type cattle. The so-called “virulent *M. haemolytica* serotypes” A1 and A6 have been linked to higher AMR in beef cattle (14, 71). However, it is not known if there is a true association between *M. haemolytica* serotype and AMR or simply virulent serotypes are more frequently exposed to antimicrobials due to treatment for BRD. The results observed in this study may be the consequence of a higher prevalence of serotype A2 among healthy animals under the high selective pressure of AMU in dairy prior to entry into the feedlot environment.

Jelinski et al. (11) proposed an SIR scheme for *M. bovis* isolated from bovine respiratory samples. Application of these criteria to *M. bovis* isolates in our study indicated high levels of resistance to TIP (100% both), TIL (100% beef and 99% dairy), and GAM (97.5% beef and 97% dairy). High MIC values for macrolides in *M. bovis* isolates from dairy and beef have been reported in Europe, Asia, and North America (11, 72). Macrolides are not frequently administrated to ranch calves in beef cow operations (58), which contrasts to the observed high macrolide MICs for *M. bovis* regardless of cattle type in our study. Antimicrobial resistance in *M. bovis* is mediated by mutations and not genes, possibly imposing a lower fitness burden, allowing the persistence of AMR traits regardless of AMU. Based on our results, TUL, ENRO, FFN, and TETs may be more effective against *M. bovis* than TIP, TIL, or GAM. However, the use of TET to treat clinical disease caused by *M. bovis* should be undertaken with caution since other authors have proposed a possible linkage between TETs and chronic pneumonia and polyarthritis syndrome in feedlots (73, 74). Nevertheless, it is not practically known which bacteria are specifically causing BRD in any one individual animal and frequently multiple bacteria can be isolated from fatal BRD cases (75). Therefore, it is difficult for veterinary practitioners to make any specific recommendations as it is not known which pathogen(s) is causing clinical disease. The odds of isolating *M. bovis* from beef and dairy-type cattle were 2.52 and 2.26 times higher, respectively, in the second year as compared to the first year of our study. During the first sampling year, a commercial media for the isolation of *M. bovis* was used (broth and agar; cat. no. TP90 and PM80, Dalynn Biologicals). Since *M. bovis* colonies growing on this commercial media often did not display the typical fried-egg morphology (76), a different media was used during the second sampling year (23). As a consequence, it is not possible to know if the difference in the *M. bovis* recovery was a consequence of inter-year variability.

Twenty-five and 68% of *M. haemolytica* and *P. multocida* isolates from dairy-type calves were MDR. As aforementioned, AMU is greater in dairy-type cattle as compared to beef-type cattle (77). Studies have suggested that feedlots are an environment that promote/amplify AMR via various means including through metaphylaxis (78, 79), serving as a reservoir of AMR bacteria and genes, selection through antimicrobial metabolites in manure (80, 81), and manure enriching the soil microbiome in AMR bacteria (82). It is known that metaphylaxis upon feedlot arrival decreases BRD morbidity and mortality (53) but it may select for AMR bacteria (78). Bovine respiratory disease *Pasteurellaceae* can persist in the farm and feedlot environment (83, 84) and be transmitted amongst different herds (85). In North America, large commercial feedlots receive a constant flow of animals and pens are usually cleaned (not disinfected) of manure/bedding once or twice per year. This constant occupancy of cattle in the feedlot may promote the persistence of AMR bacteria in the environment. Some feedlots feed both beef and dairy-type cattle, resulting in cattle with significantly different AMU backgrounds being housed in close proximity. Many of the MDR *Pasteurellaceae* harbor ARGs on ICE and it is unknown if bacteria carrying these elements increase and spread among cattle within the feedlot, a possibility that deserves further evaluation.

## Conclusion

This is the first published study in Alberta documenting AMR in four major bacterial species involved in the BRD complex isolated from beef and dairy-type cattle on feedlot arrival. Our findings show marked differences in bacterial isolation and AMR levels in bacterial members of the BRD complex between dairy and beef cattle types. Moreover, MDR *M. haemolytica, P. multocida*, and *H. somni* isolates presenting AMR phenotypes indicative of the presence of ICE were isolated more often from dairy-type than beef-type cattle. These results raise the question of whether feedlot AMU and AMR should be reported by cattle type which could help to evaluate if the higher prevalence of ICE-related AMR is linked to higher BRD treatment failure and mortality. Additionally, an association between higher AMR and feedlot-backgrounded beef-type cattle was reported. Macrolides may not be an efficacious treatment choice for *M. bovis* (in beef and dairy-type cattle) or *H. somni* (dairy) infections in feedlot cattle in Alberta. Considering that antimicrobial therapy is essential for the prevention, control, and treatment of BRD in feedlot cattle, our study highlights the continued need for AMU and AMR surveillance of BRD-related bacteria in feedlot cattle to help inform veterinarians on treatment protocol decisions that promote prudent drug use.

## Data Availability Statement

The data presented in the study are deposited in the NCBI repository, accession number PRJNA720670.

## Ethics Statement

The animal study was reviewed and approved by the Lethbridge Research Centre Animal Care and Use Committee (Protocol #1641, Jan 18th, 2017) and was conducted according to the Canadian Council of Animal Care Guidelines. Written informed consent for participation was not obtained from the owners because Consent was provided verbally as a result of communication between veterinary practitioners and their clients.

## Author Contributions

JV, CB, SJH, SH, CD, SG, and TM: concept and design of the study. CB, CD, JV, SH, and SJH: sample collection. CL, SA-L, RH, and RZ: data acquisition. RZ, SA-L, and RD: analysis of data. RZ, SA-L, and MA: interpretation of data. SA-L: drafting the manuscript. CB, CD, CL, JV, MA, RD, RH, RZ, SG, SH, SJH, and TM: critical review of the manuscript including the final version. All authors contributed to the article and approved the submitted version.

## Conflict of Interest

CB is part owner and managing partner of Feedlot Health Management Services Ltd. and Southern Alberta Veterinary Services. SJH is an employee at Feedlot Health Management Services Ltd., Okotoks, Alberta, Canada. Feedlot Health is a private company that provides expert consultation regarding production and management of feedlot cattle and calf grower calves, including developing veterinary protocols to support animal health. They also conduct in-house and contract research related to dairy calf grower and feedlot production. The remaining authors declare that the research was conducted in the absence of any commercial or financial relationships that could be construed as a potential conflict of interest.

## Abbreviations

A: auction
AF: arrived from
AMG: aminoglycoside
AMP: ampicillin
AMR: antimicrobial resistance
AMU: antimicrobial use
ARG: antimicrobial resistance gene
B: backgrounding
BHI: brain-heart infusion
BRD: bovine respiratory disease
CI: Confidence Interval
CLSI: Clinical an Laboratory Standards Institute
cPCR: conventional PCR
CTET: chlortetracycline
DANO: danofloxacin
DNPS: deep nasopharyngeal swab
ENRO: enrofloxacin
FFN: florfenicol
FQ: fluoroquinolone
GLMM: generalized linear mixed model
GAM: gamithromycin
GEN: gentamycin
MDR: multidrug resistance
ICE: integrative and conjugative element
MGE: mobile genetic element
MIC: minimum inhibitory concentration
NEO: neomycin
OR: odds ratio
OXY: oxytetracycline
PEN: penicillin
qPCR: quantitative PCR
QRDR: quinolone resistant determining region
RD: ranch direct
RE: random effect
RFID: radio frequency identification
SIR: susceptible, intermediate, resistant
SPE: spectinomycin
TC: truck cluster
TET: tetracycline
TIL: tilmicosin
TIO: ceftiofur
TIP: tildipirosin
TMR: total mixed ration
TUL: tulathromycin
TYLT: tylosin tartrate
WGS: whole genome sequencing.
