# Supplementary material for: Prevalence and Risk Factors Associated With Antimicrobial Resistance in Bacteria Related to Bovine Respiratory Disease—A Broad Cross-Sectional Study of Beef Cattle at Entry Into Canadian Feedlots
Source: Front Vet Sci. 2021 Jul 1;8:692646. doi: 10.3389/fvets.2021.692646 (PMC8280473; doi:10.3389/fvets.2021.692646)
Supplement: Supplementary file 5 [file Data_Sheet_1.PDF]

Supplementary Figure 1.1 Proportion of antimicrobial resistance unique profiles isolated from deep pharyngeal nasal swabs collected from beef and dairy cattle upon feedlot arrival

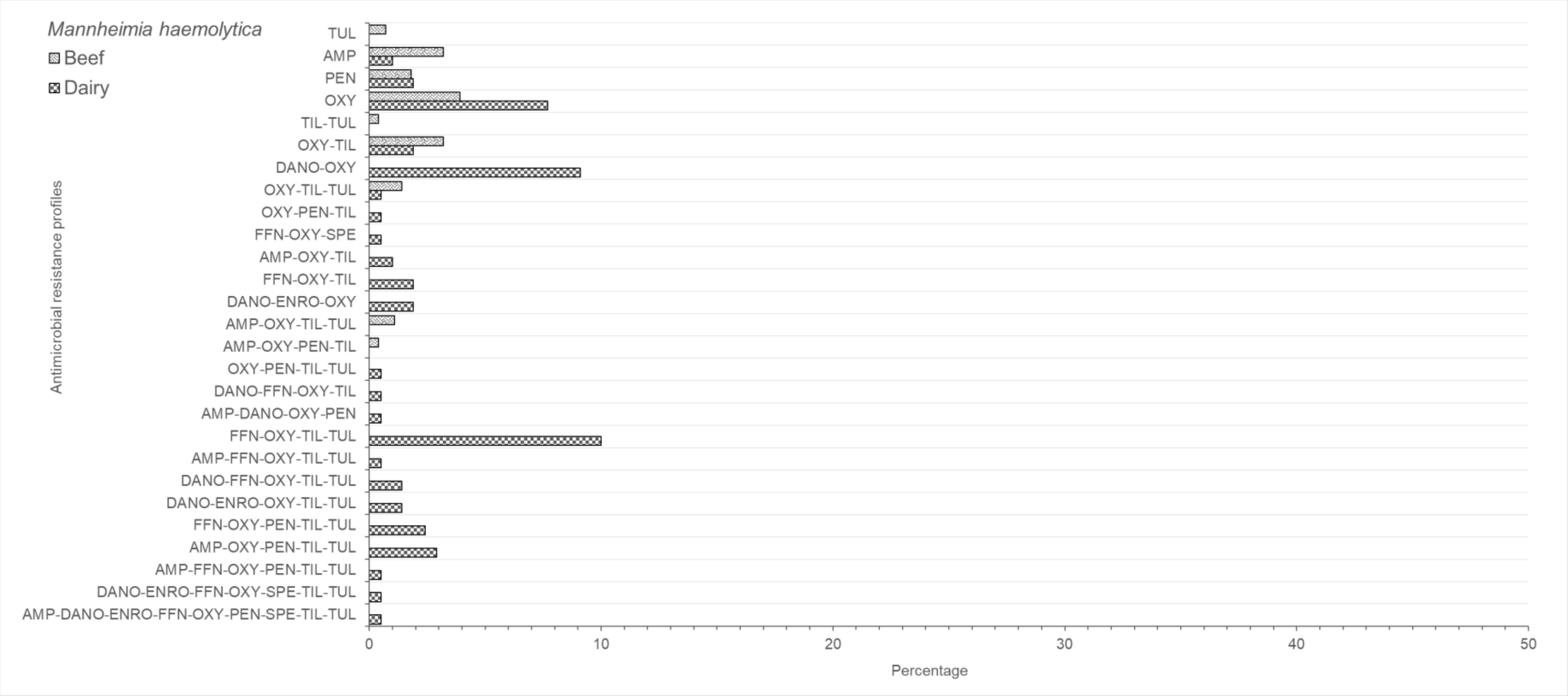

Supplementary Figure 1.1 Proportion of antimicrobial resistance unique profiles isolated from deep pharyngeal nasal swabs collected from beef and dairy cattle upon feedlot arrival (*continued*)

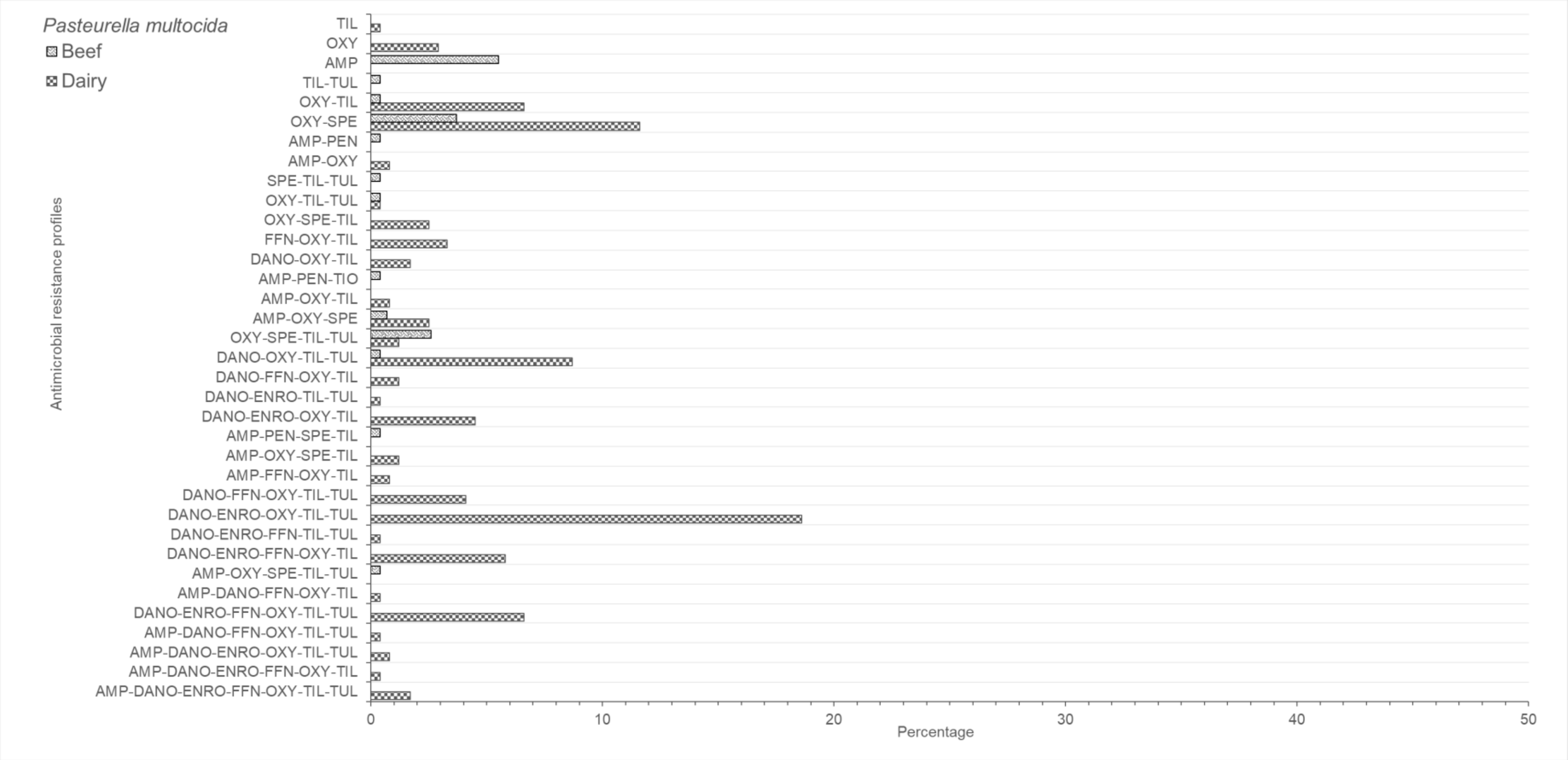

Supplementary Figure 1.1 Proportion of antimicrobial resistance unique profiles isolated from deep pharyngeal nasal swabs collected from beef and dairy cattle upon feedlot arrival (*continued*)

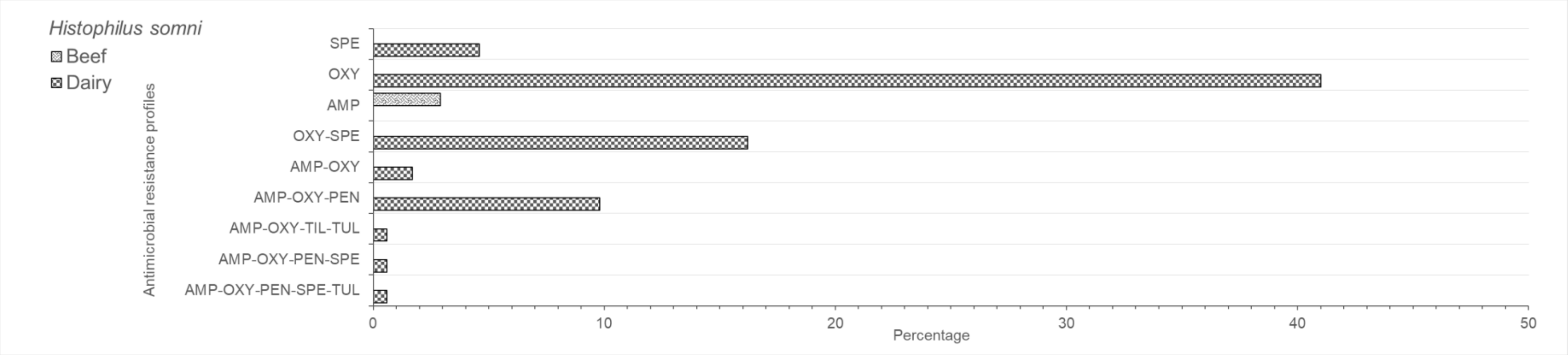

AMP, ampicillin; DANO, danofloxacin; ENRO, enrofloxacin; FFN, florfenicol; OXY, oxytetracycline; PEN, penicillin; SPE, spectinomycin; TIL, tilmicosin; TIO, ceftiofur; TUL, tulathromycin.

Supplementary Figure 1.2 Susceptible, intermediate, and resistant relative proportions of *Mannheimia haemolytica* serotypes (A1, A6, A2) isolated from deep pharyngeal nasal swabs collected from beef and dairy cattle upon feedlot arrival

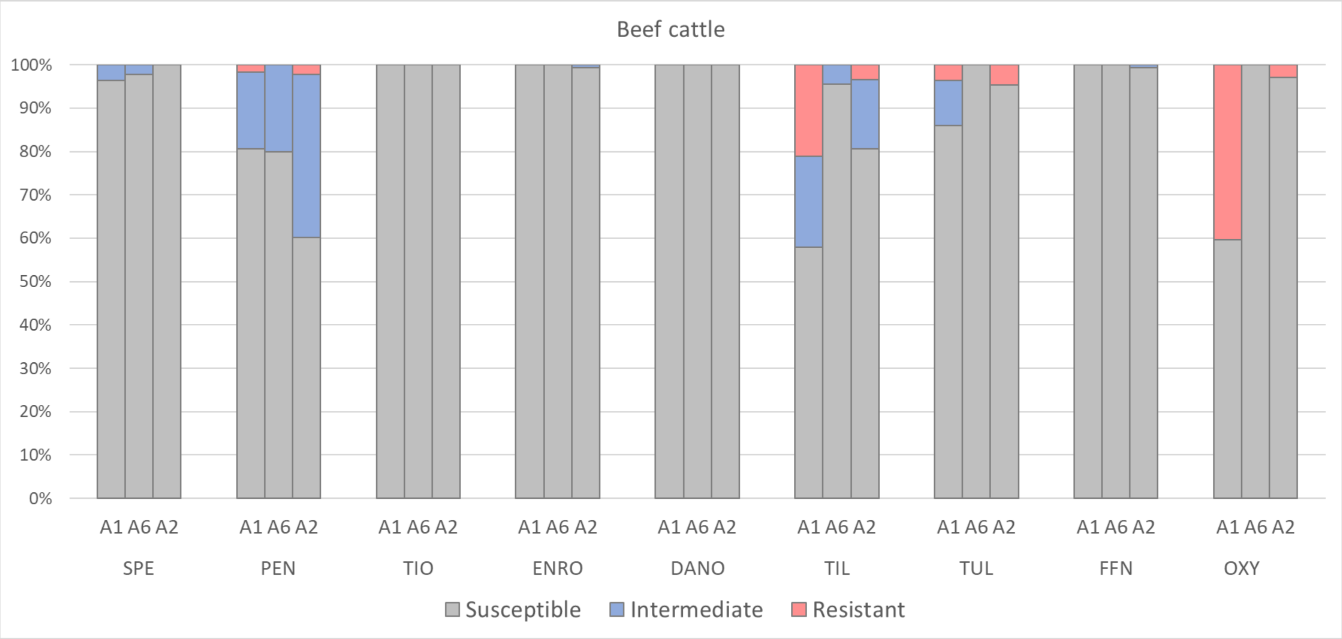

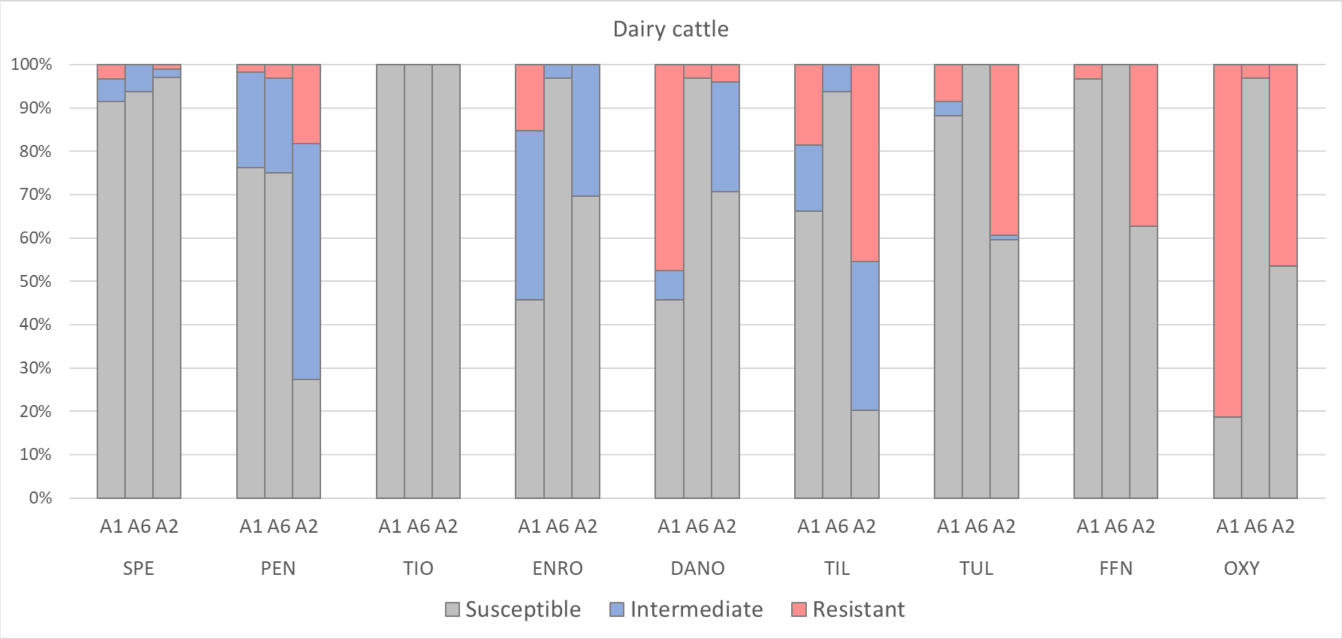

DANO, danofloxacin; ENRO, enrofloxacin; FFN, florfenicol; OXY, oxytetracycline; PEN, penicillin; SPE, spectinomycin; TIL, tilmicosin; TUL, tulathromycin.

Supplementary Figure 1.3 Heatmap with dendrogram of the MIC values from bacterial species isolated from deep pharyngeal nasal swabs collected from beef and dairy cattle upon feedlot arrival

Supplementary Figure 1.3.1 Unadjusted *Mannheimia haemolytica* normalized MIC distributions clustered by cattle type

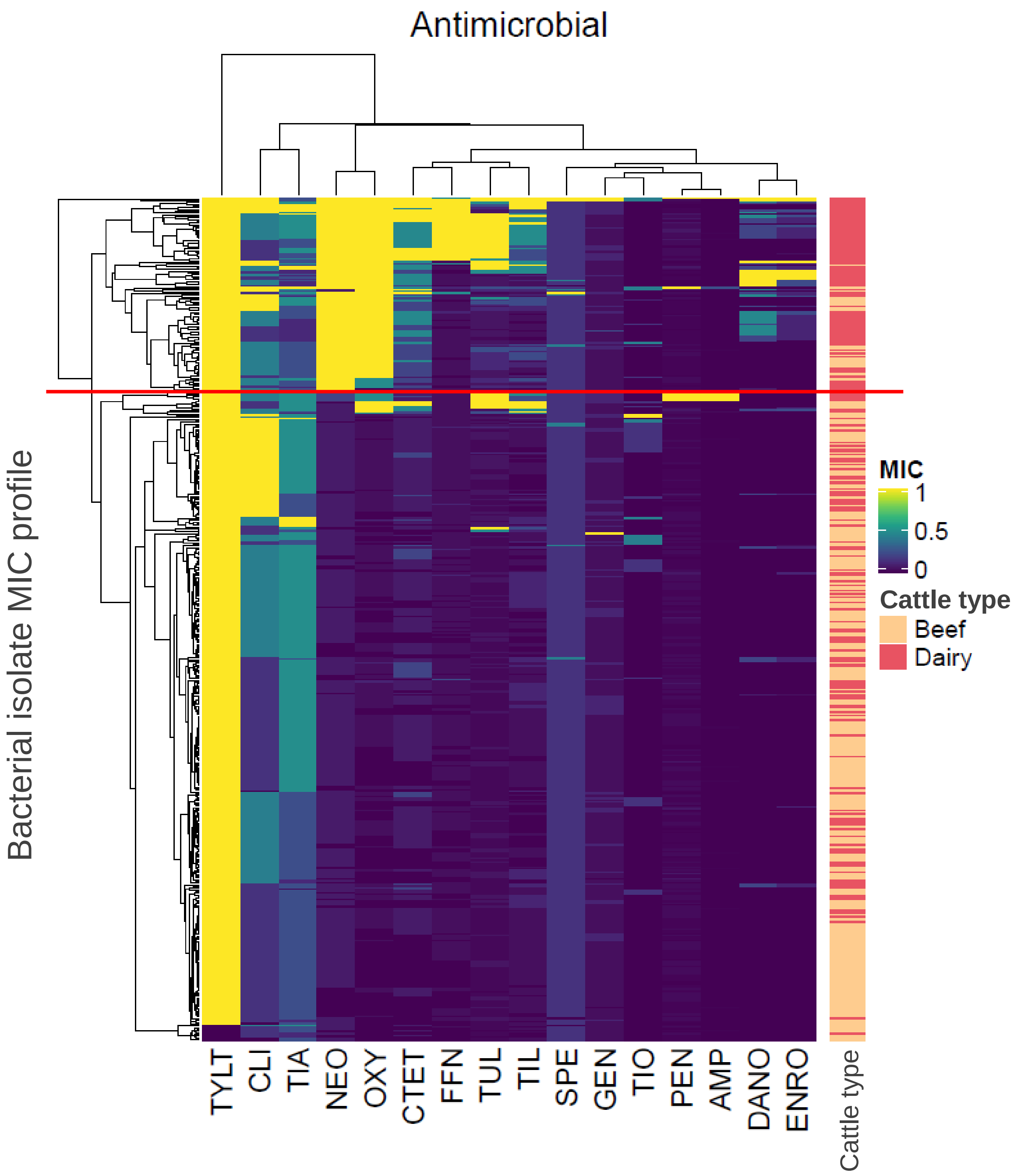

AMP, ampicillin; CLI, clindamycin; CTET, chlortetracycline; DANO, danofloxacin; ENRO, enrofloxacin; FFN, florfenicol; GEN, gentamycin; MIC, minimum inhibitory concentration; NEO, neomycin; OXY, oxytetracycline; PEN, penicillin; SPE, spectinomycin; TIA, tiamulin; TIL, tilmicosin; TIO, ceftiofur; TUL, tulathromycin; TYLT, tylosin tartrate. The red line defines two different bacterial populations grouped by antimicrobial susceptibility profiles.

Supplementary Figure 1.3.2 Unadjusted *Pasteurella multocida* normalized MIC distributions clustered by cattle type

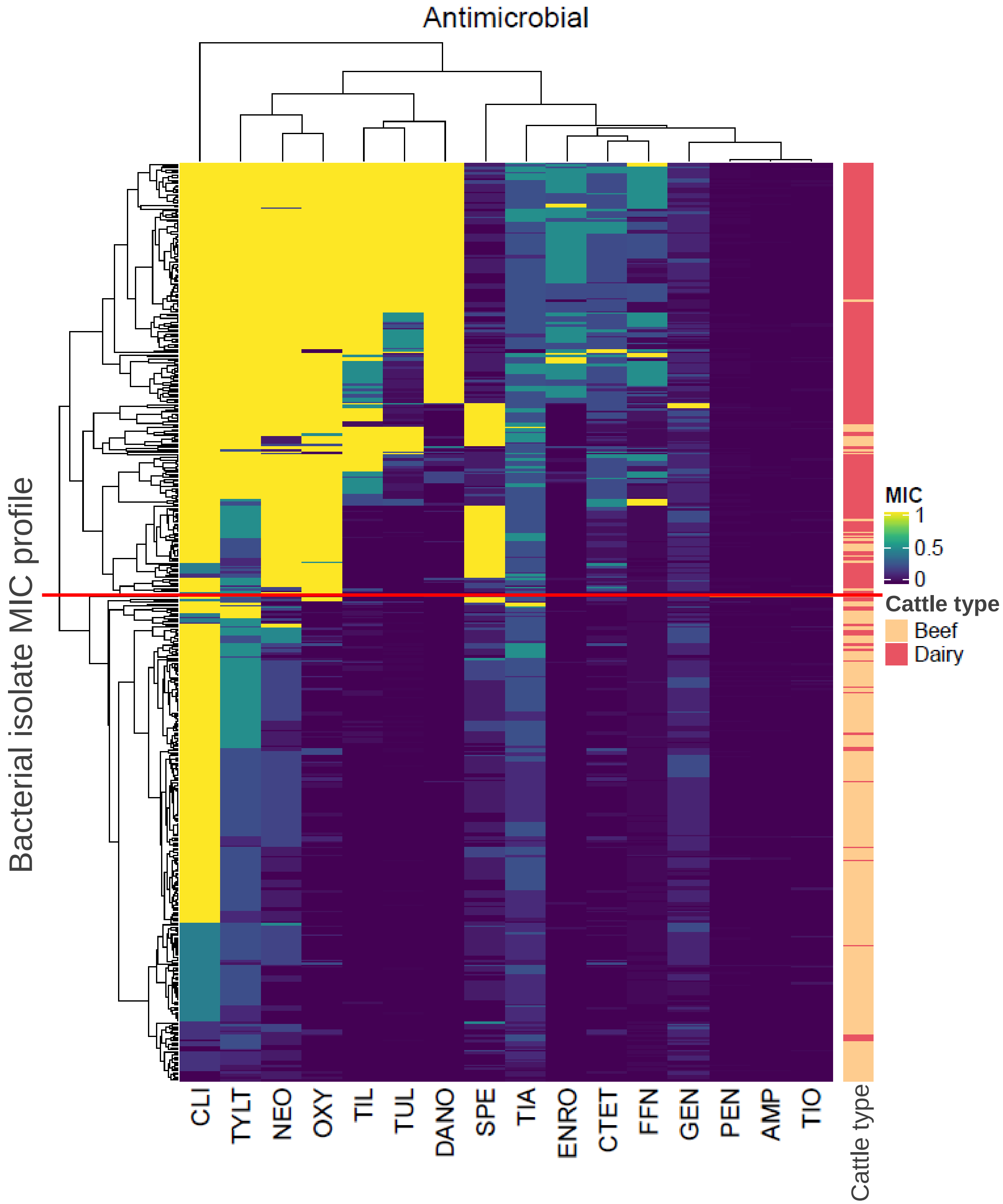

AMP, ampicillin; CLI, clindamycin; CTET, chlortetracycline; DANO, danofloxacin; ENRO, enrofloxacin; FFN, florfenicol; GEN, gentamycin; MIC, minimum inhibitory concentration; NEO, neomycin; OXY, oxytetracycline; PEN, penicillin; SPE, spectinomycin; TIA, tiamulin; TIL, tilmicosin; TIO, ceftiofur; TUL, tulathromycin; TYLT, tylosin tartrate. The red line defines two different bacterial populations grouped by antimicrobial susceptibility profiles.

Supplementary Figure 1.3.3 Unadjusted *Histophilus somni* normalized MIC distributions clustered by cattle type

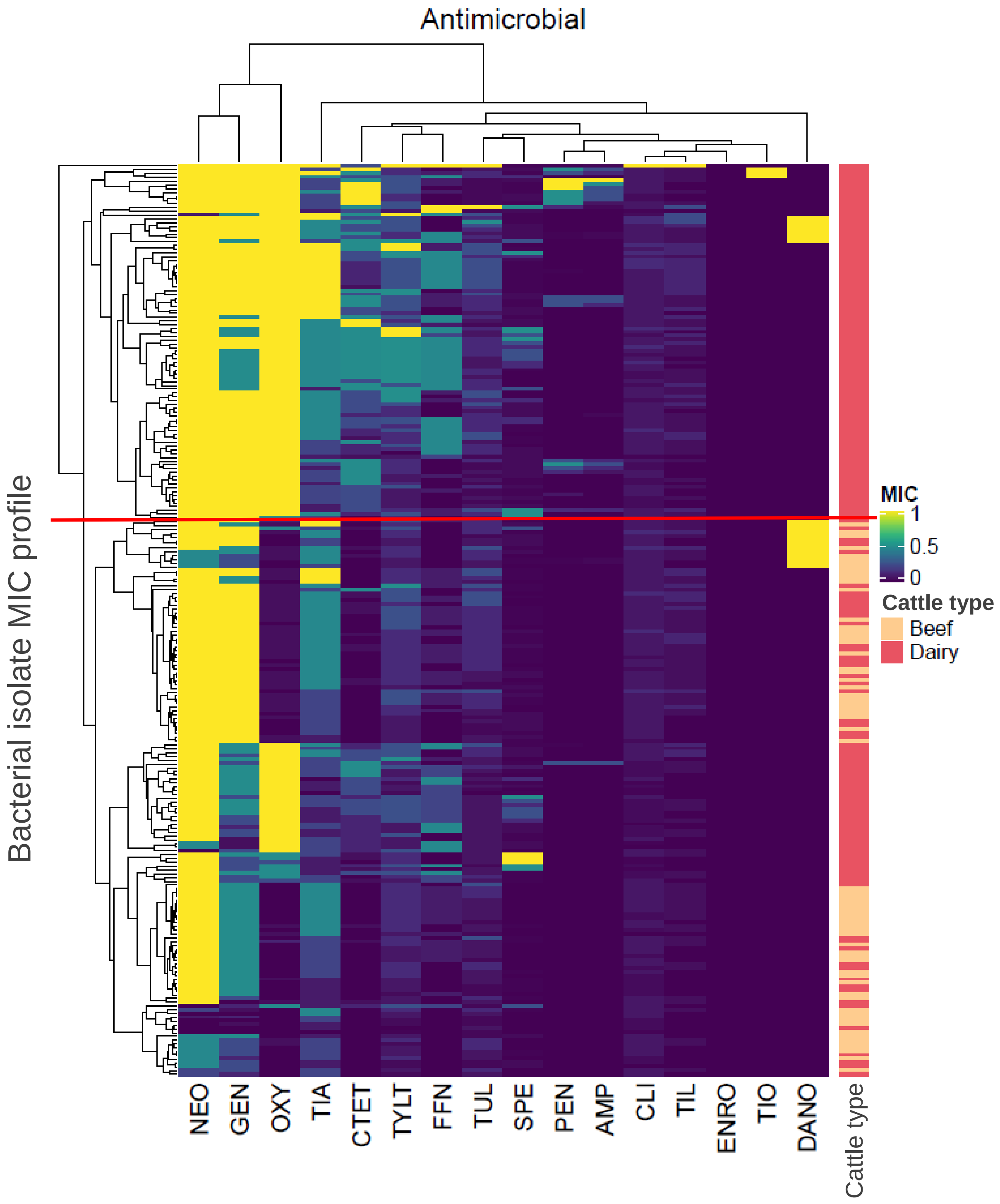

AMP, ampicillin; CLI, clindamycin; CTET, chlortetracycline; DANO, danofloxacin; ENRO, enrofloxacin; FFN, florfenicol; GEN, gentamycin; MIC, minimum inhibitory concentration; NEO, neomycin; OXY, oxytetracycline; PEN, penicillin; SPE, spectinomycin; TIA, tiamulin; TIL, tilmicosin; TIO, ceftiofur; TUL, tulathromycin; TYLT, tylosin tartrate. The red line defines two different bacterial populations grouped by antimicrobial susceptibility profiles.

Supplementary Figure 1.3.4 Unadjusted *Mycoplasma bovis* normalized MIC distributions clustered by cattle type

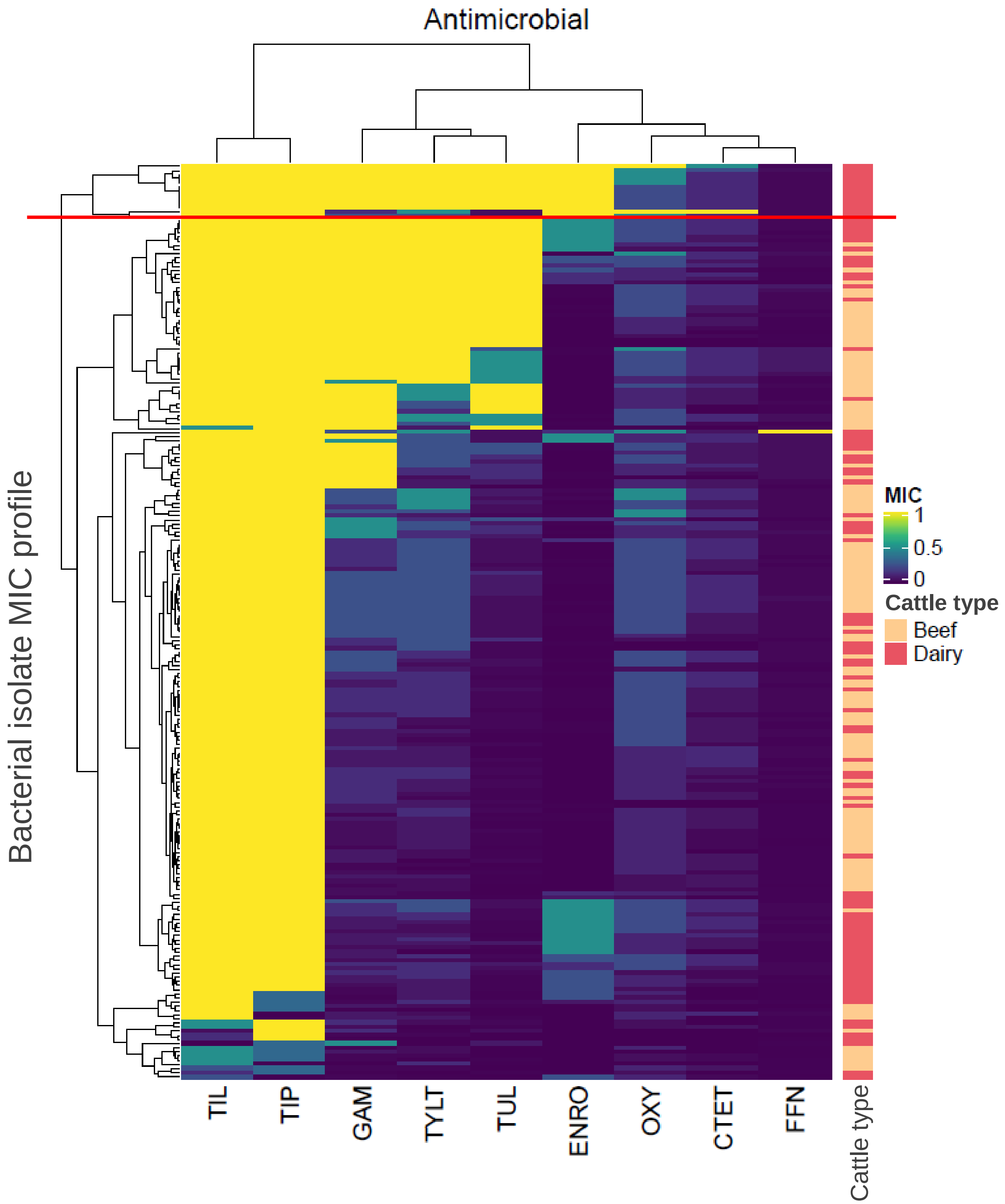

AMP, ampicillin; CLI, clindamycin; CTET, chlortetracycline; DANO, danofloxacin; ENRO, enrofloxacin; FFN, florfenicol; GEN, gentamycin; MIC, minimum inhibitory concentration; NEO, neomycin; OXY, oxytetracycline; PEN, penicillin; SPE, spectinomycin; TIA, tiamulin; TIL, tilmicosin; TIO, ceftiofur; TUL, tulathromycin; TYLT, tylosin tartrate. The red line defines two different bacterial populations grouped by antimicrobial susceptibility profiles.

Supplementary Figure 1.3.5 Unadjusted *Mannheimia haemolytica* normalized MIC distributions stratified by cattle type and clustered by country of origin

A) Beef cattle B) Dairy cattle

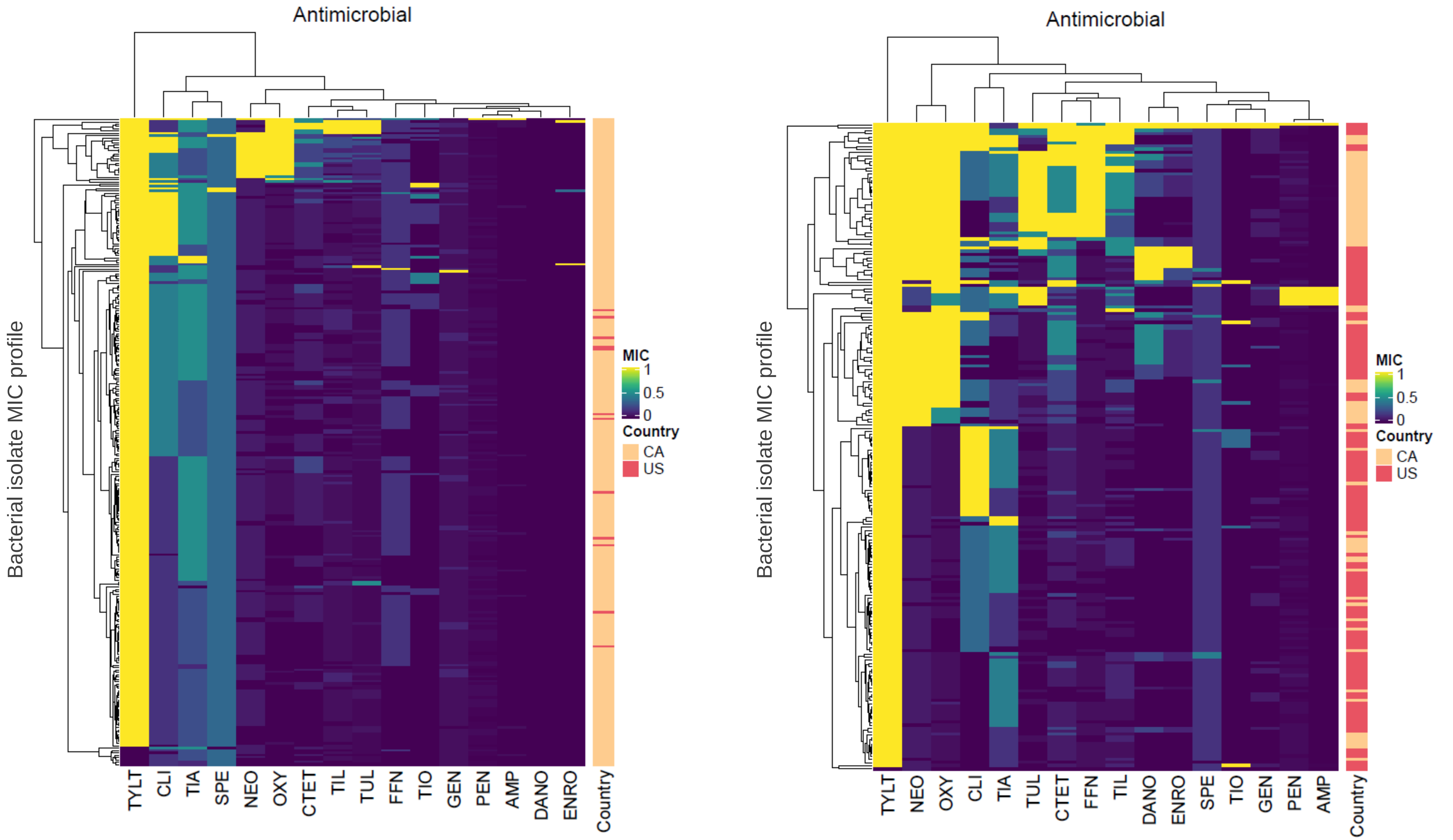

AMP, ampicillin; CA, Canada; CLI, clindamycin; CTET, chlortetracycline; DANO, danofloxacin; ENRO, enrofloxacin; FFN, florfenicol; GEN, gentamycin; MIC, minimum inhibitory concentration; NEO, neomycin; OXY, oxytetracycline; PEN, penicillin; SPE, spectinomycin; TIA, tiamulin; TIL, tilmicosin; TIO, ceftiofur; TUL, tulathromycin; TYLT, tylosin tartrate.

Supplementary Figure 1.3.6 Unadjusted *Mannheimia haemolytica* normalized MIC distributions stratified by cattle type and clustered by monthly interval

A) Beef cattle   B) Dairy cattle

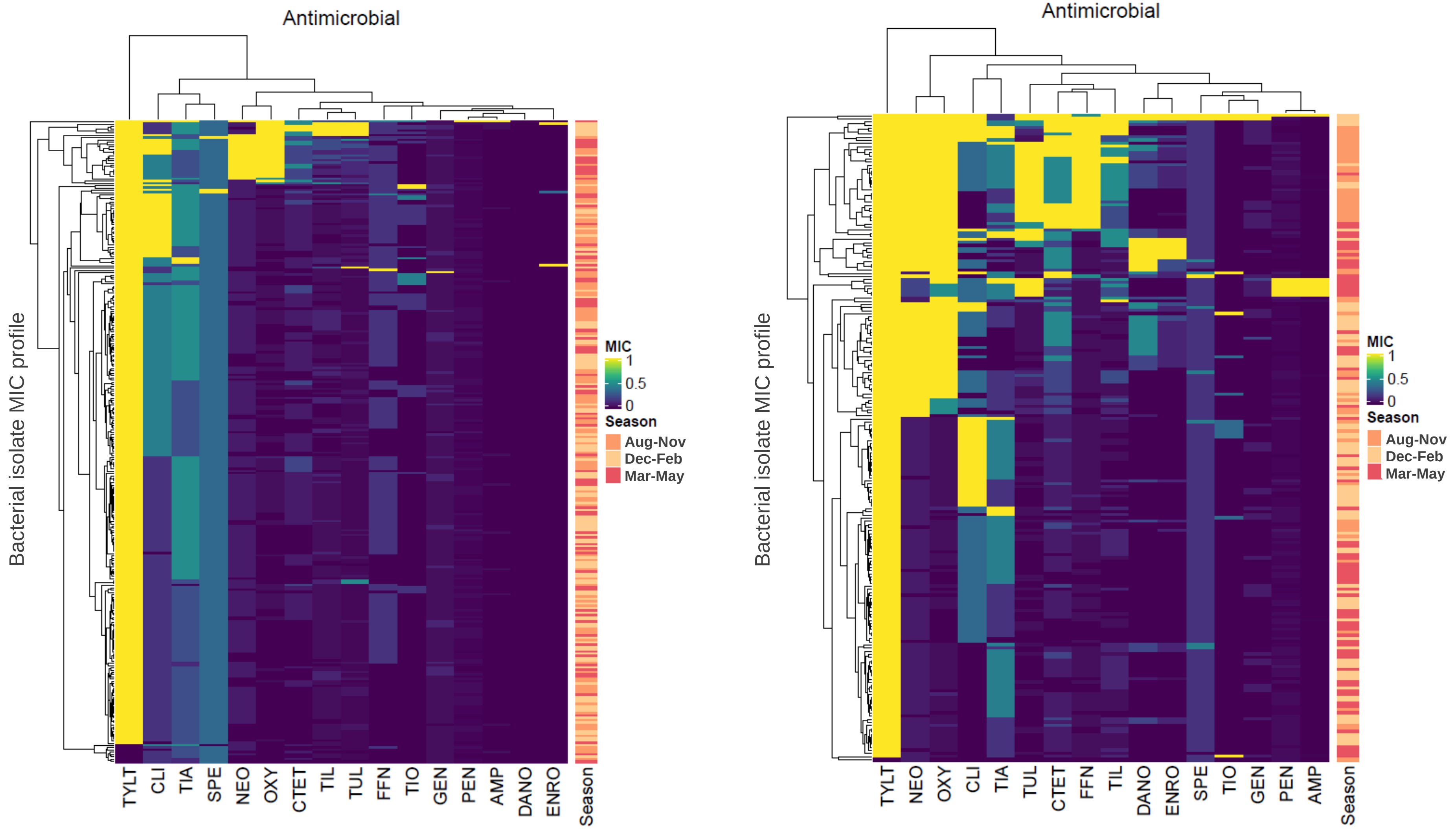

AMP, ampicillin; CLI, clindamycin; CTET, chlortetracycline; DANO, danofloxacin; ENRO, enrofloxacin; FFN, florfenicol; GEN, gentamycin; MIC, minimum inhibitory concentration; NEO, neomycin; OXY, oxytetracycline; PEN, penicillin; SPE, spectinomycin; TIA, tiamulin; TIL, tilmicosin; TIO, ceftiofur; TUL, tulathromycin; TYLT, tylosin tartrate.

Supplementary Figure 1.3.7 Unadjusted *Mannheimia haemolytica* normalized MIC distributions stratified by cattle type and clustered by source

A) Beef cattle B) Dairy cattle

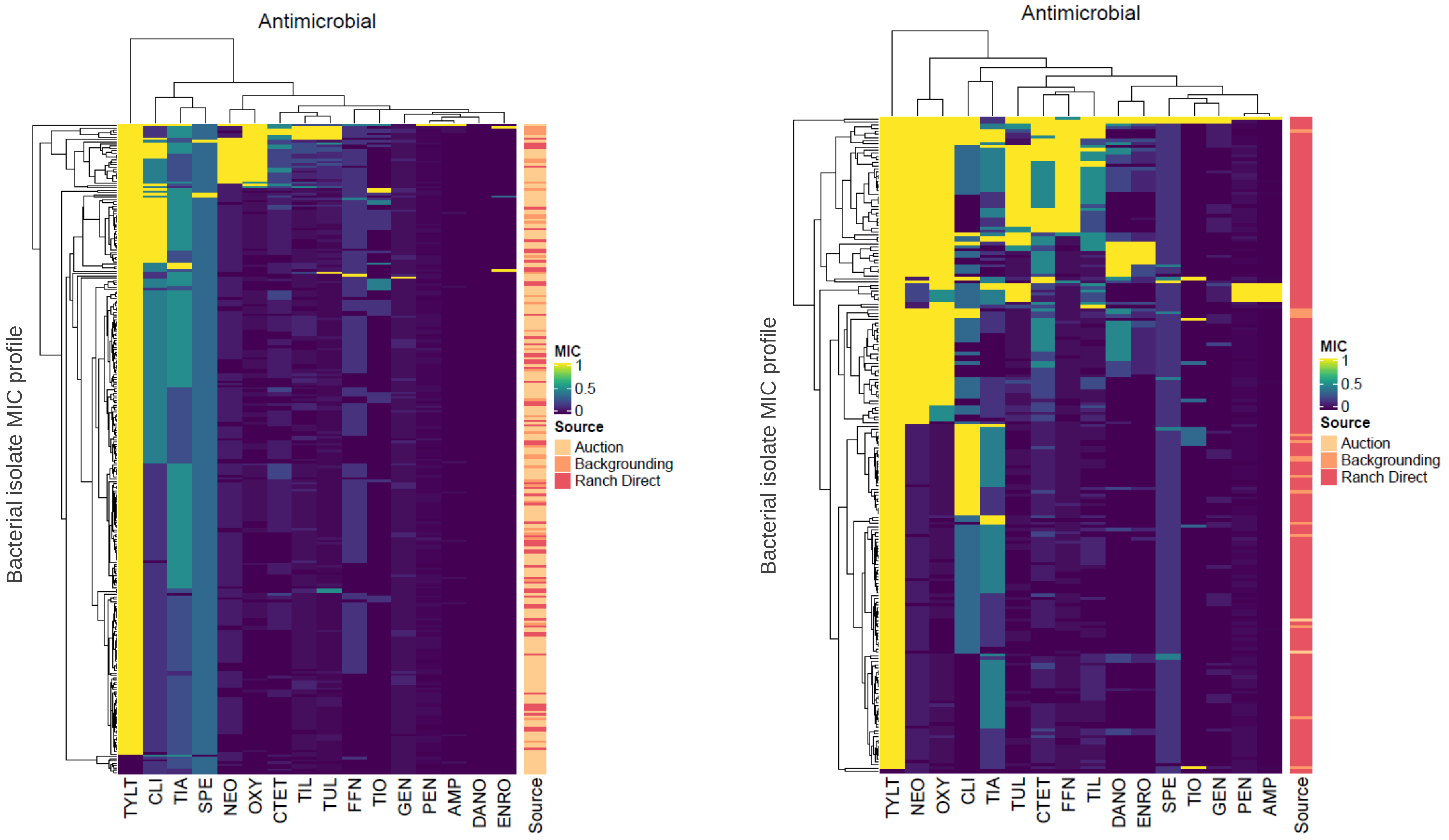

AMP, ampicillin; Backgrounding operations; CLI, clindamycin; CTET, chlortetracycline; DANO, danofloxacin; ENRO, enrofloxacin; FFN, florfenicol; GEN, gentamycin; MIC, minimum inhibitory concentration; NEO, neomycin; OXY, oxytetracycline; PEN, penicillin; SPE, spectinomycin; TIA, tiamulin; TIL, tilmicosin; TIO, ceftiofur; TUL, tulathromycin; TYLT, tylosin tartrate.

Supplementary Figure 1.3.8 Unadjusted *Mannheimia haemolytica* normalized MIC distributions stratified by cattle type and clustered by risk of suffering BRD

A) Beef cattle B) Dairy cattle

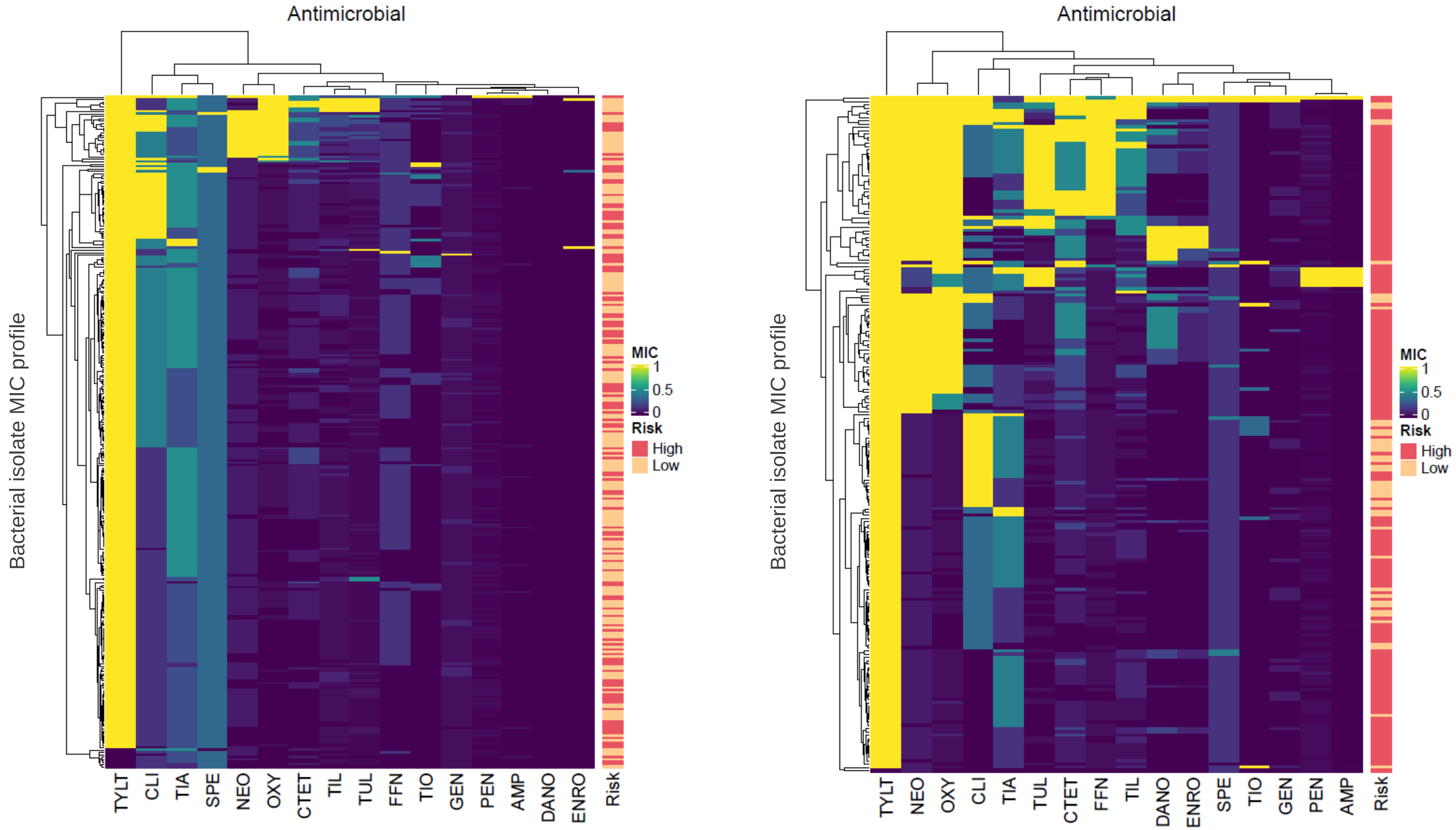

AMP, ampicillin; CLI, clindamycin; CTET, chlortetracycline; DANO, danofloxacin; ENRO, enrofloxacin; FFN, florfenicol; GEN, gentamycin; MIC, minimum inhibitory concentration; NEO, neomycin; OXY, oxytetracycline; PEN, penicillin; SPE, spectinomycin; TIA, tiamulin; TIL, tilmicosin; TIO, ceftiofur; TUL, tulathromycin; TYLT, tylosin tartrate.

Supplementary Figure 1.3.9 Unadjusted *Mannheimia haemolytica* normalized MIC distributions stratified by cattle type clustered by weight range

A) Beef cattle

B) Dairy cattle

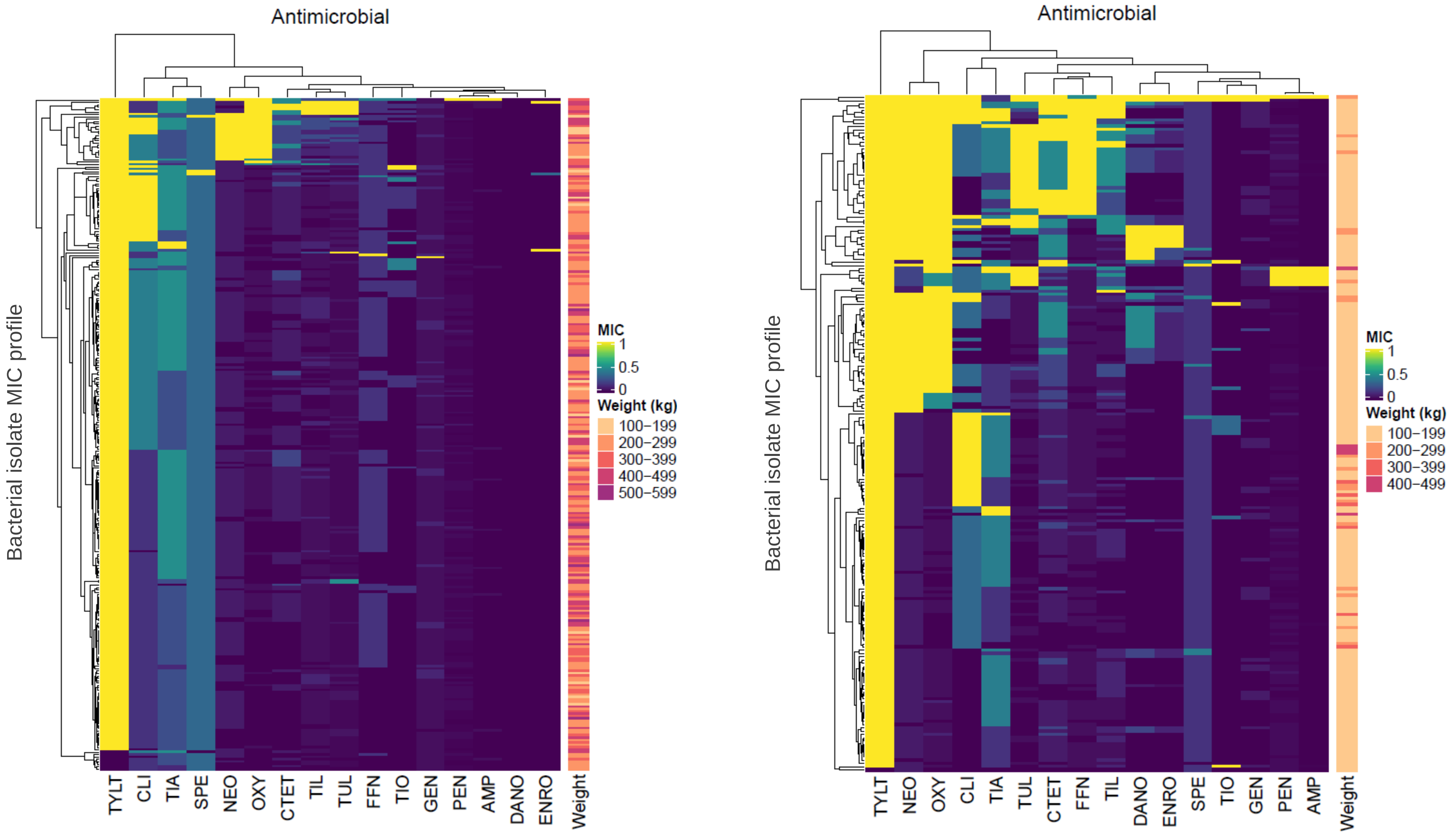

AMP, ampicillin; CLI, clindamycin; CTET, chlortetracycline; DANO, danofloxacin; ENRO, enrofloxacin; FFN, florfenicol; GEN, gentamycin; MIC, minimum inhibitory concentration; NEO, neomycin; OXY, oxytetracycline; PEN, penicillin; SPE, spectinomycin; TIA, tiamulin; TIL, tilmicosin; TIO, ceftiofur; TUL, tulathromycin; TYLT, tylosin tartrate.

Supplementary Figure 1.3.10 Unadjusted *Mannheimia haemolytica* normalized MIC distributions stratified by cattle type clustered by sex

A) Beef cattle

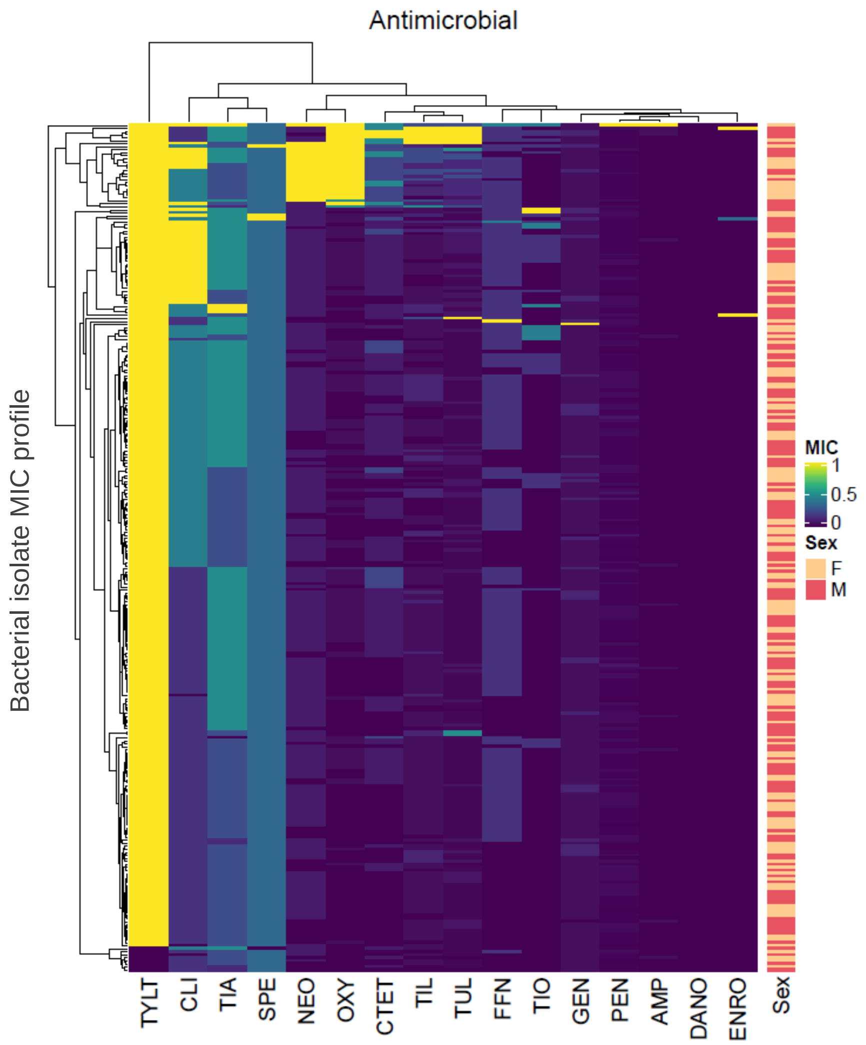

B) Dairy cattle

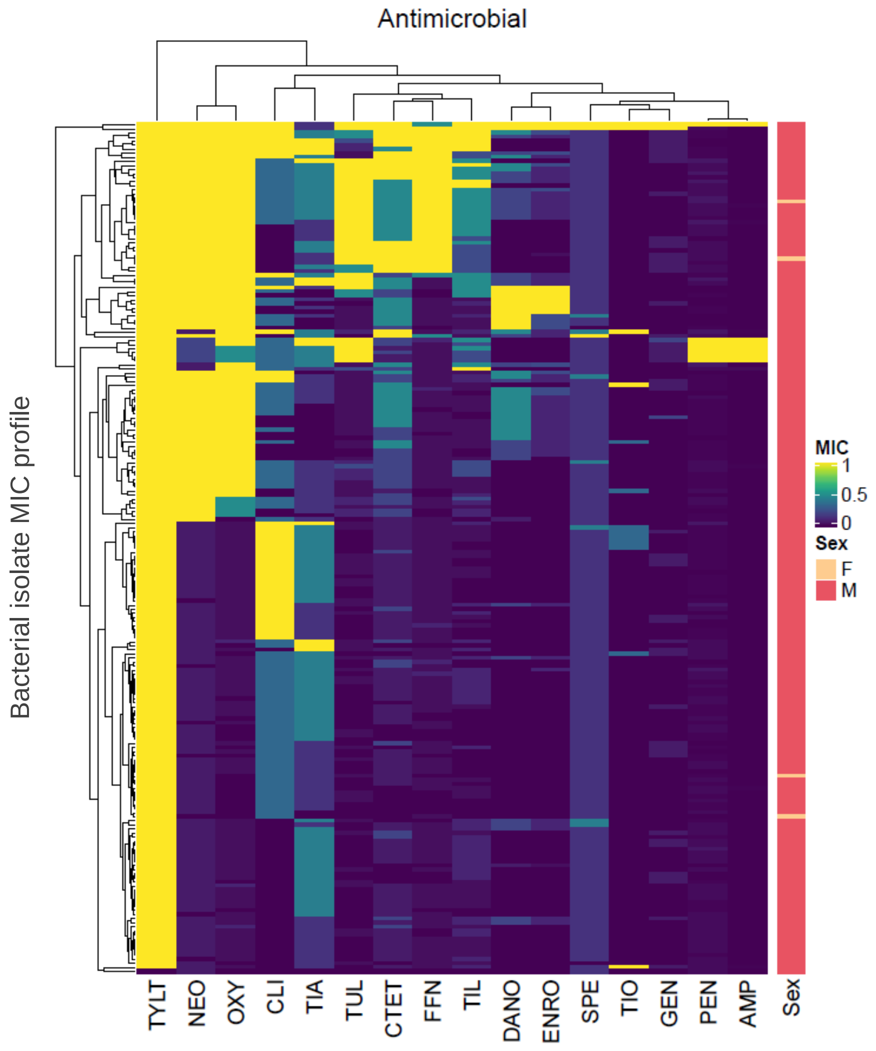

AMP, ampicillin; CLI, clindamycin; CTET, chlortetracycline; DANO, danofloxacin; ENRO, enrofloxacin; F, female; FFN, florfenicol; GEN, gentamycin; M, male; MIC, minimum inhibitory concentration; NEO, neomycin; OXY, oxytetracycline; PEN, penicillin; SPE, spectinomycin; TIA, tiamulin; TIL, tilmicosin; TIO, ceftiofur; TUL, tulathromycin; TYLT, tylosin tartrate.

Supplementary Figure 1.3.11 Unadjusted *Mannheimia haemolytica* normalized MIC distributions stratified by cattle type and clustered by age

A) Beef cattle

B) Dairy cattle

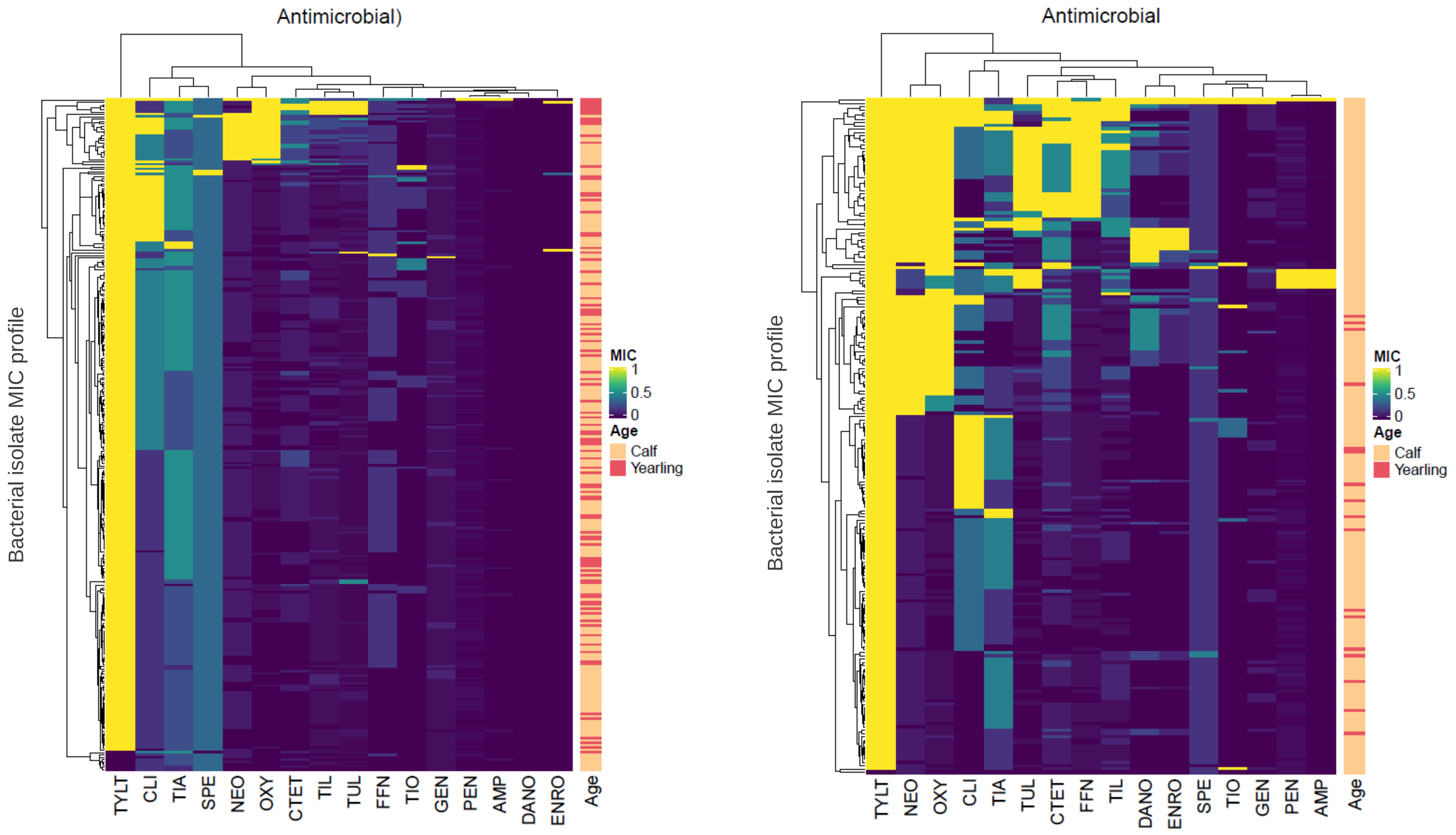

AMP, ampicillin; CLI, clindamycin; CTET, chlortetracycline; DANO, danofloxacin; ENRO, enrofloxacin; FFN, florfenicol; GEN, gentamycin; MIC, minimum inhibitory concentration; NEO, neomycin; OXY, oxytetracycline; PEN, penicillin; SPE, spectinomycin; TIA, tiamulin; TIL, tilmicosin; TIO, ceftiofur; TUL, tulathromycin; TYLT, tylosin tartrate.

Supplementary Figure 1.3.12 Unadjusted *Mannheimia haemolytica* normalized MIC distributions stratified by cattle type and clustered by temperature range

A) Beef cattle B) Dairy cattle

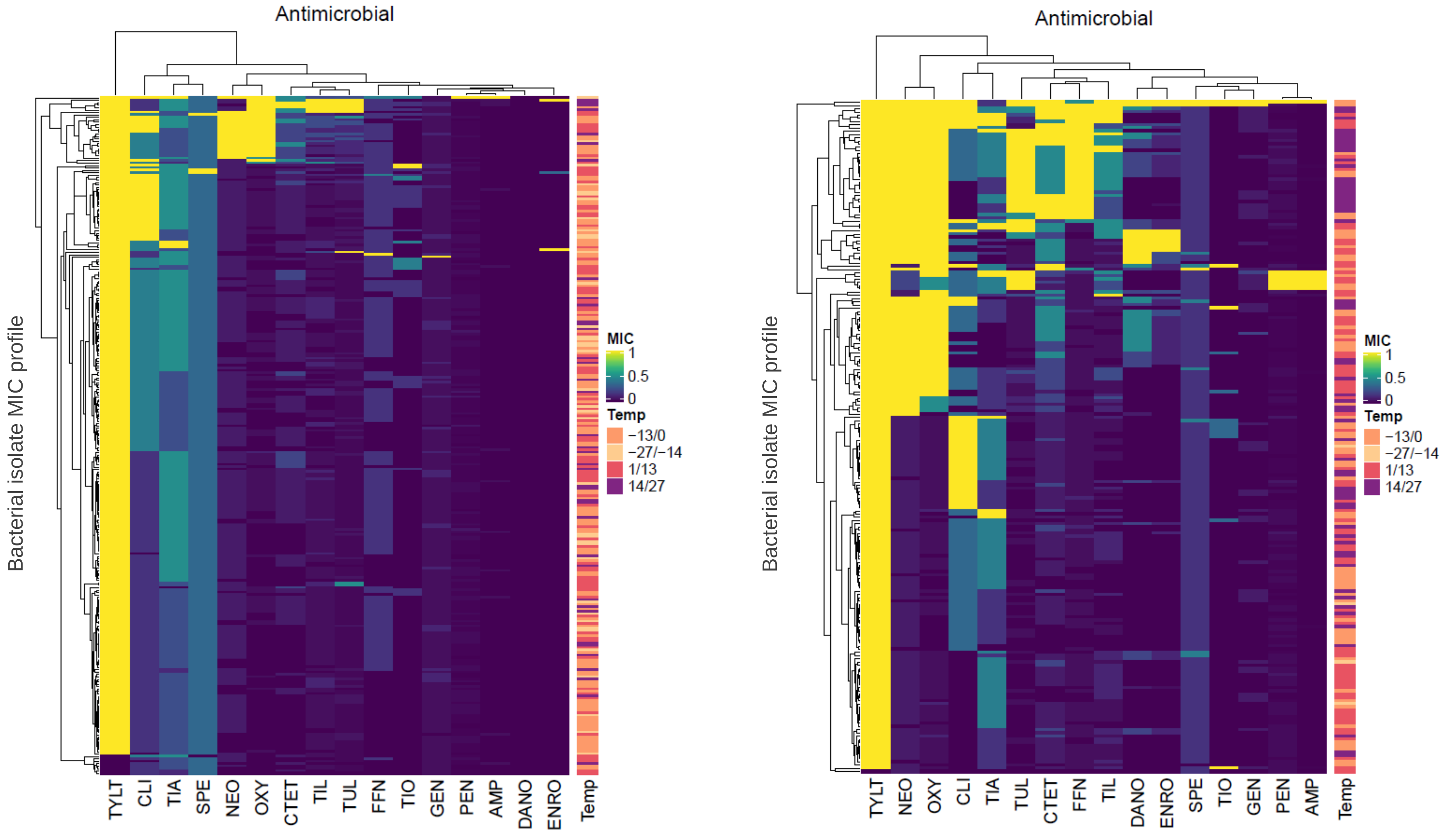

AMP, ampicillin; CLI, clindamycin; CTET, chlortetracycline; DANO, danofloxacin; ENRO, enrofloxacin; FFN, florfenicol; GEN, gentamycin; MIC, minimum inhibitory concentration; NEO, neomycin; OXY, oxytetracycline; PEN, penicillin; SPE, spectinomycin; Temp, temperature (°C); TIA, tiamulin; TIL, tilmicosin; TIO, ceftiofur; TUL, tulathromycin; TYLT, tylosin tartrate.

Supplementary Figure 1.3.13 Unadjusted *Mannheimia haemolytica* normalized MIC distributions stratified by cattle type and clustered by serotype

A) Beef cattle

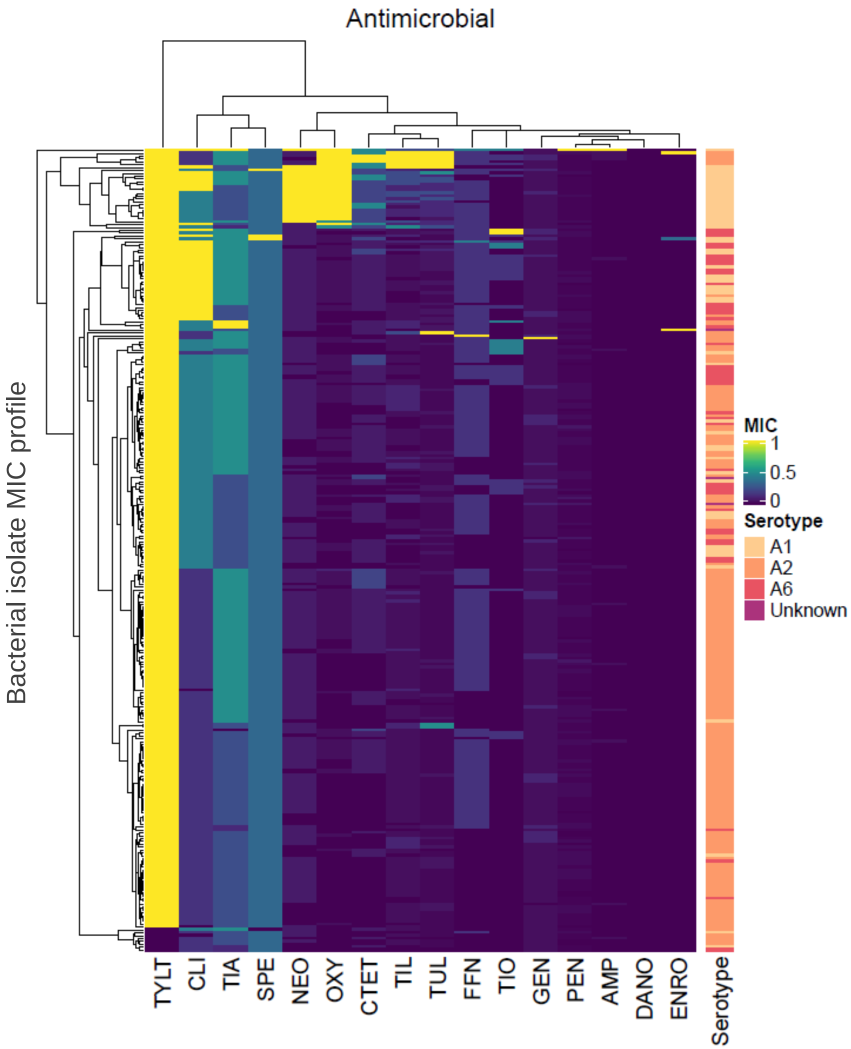

B) Dairy cattle

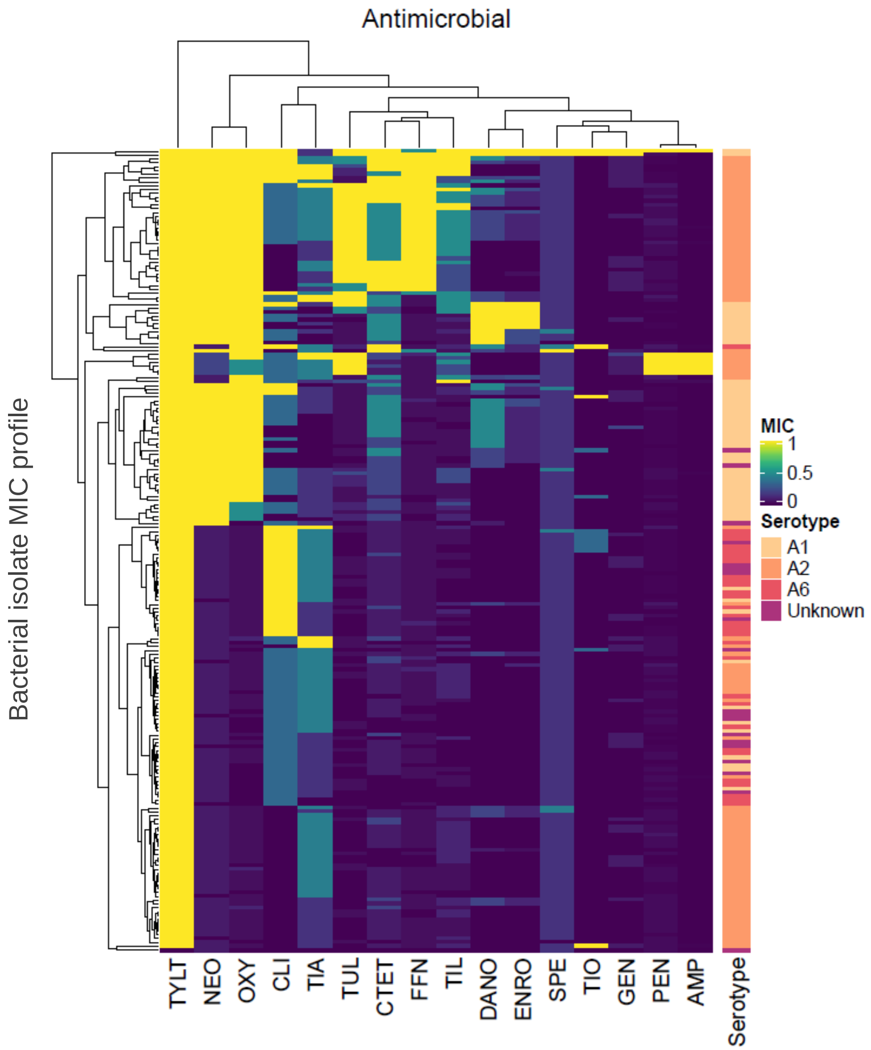

AMP, ampicillin; CLI, clindamycin; CTET, chlortetracycline; DANO, danofloxacin; ENRO, enrofloxacin; FFN, florfenicol; GEN, gentamycin; MIC, minimum inhibitory concentration; NEO, neomycin; OXY, oxytetracycline; PEN, penicillin; SPE, spectinomycin; TIA, tiamulin; TIL, tilmicosin; TIO, ceftiofur; TUL, tulathromycin; TYLT, tylosin tartrate.

Supplementary Figure 1.3.14 Unadjusted *Mannheimia haemolytica* normalized MIC distributions stratified by cattle type and clustered by BRD-related morbidity

A) Beef cattle

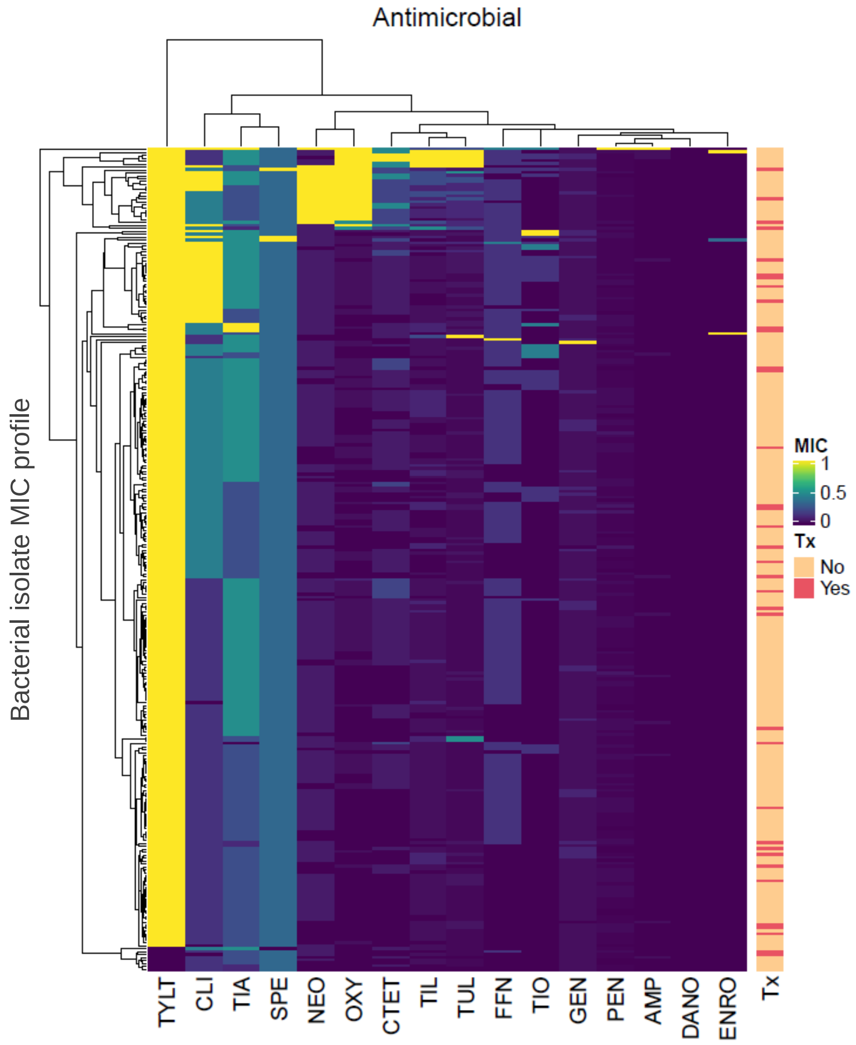

B) Dairy cattle

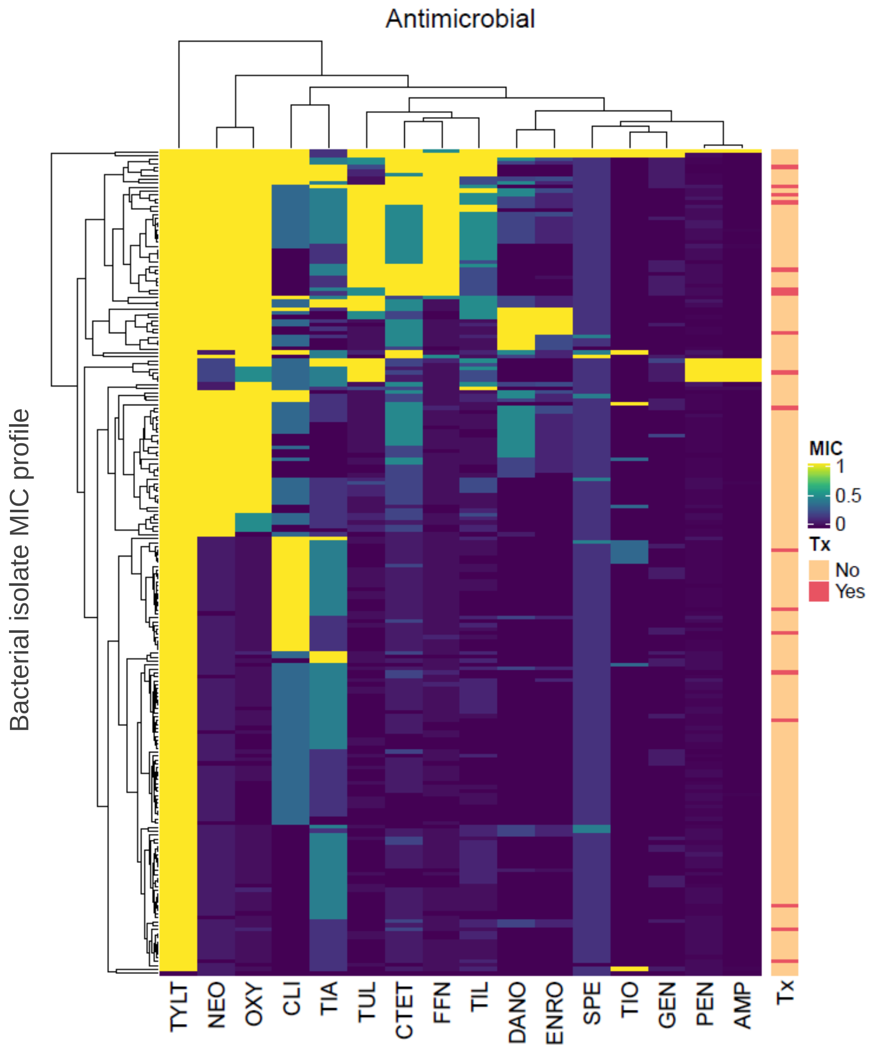

AMP, ampicillin; CLI, clindamycin; CTET, chlortetracycline; DANO, danofloxacin; ENRO, enrofloxacin; FFN, florfenicol; GEN, gentamycin; MIC, minimum inhibitory concentration; NEO, neomycin; OXY, oxytetracycline; PEN, penicillin; SPE, spectinomycin; TIA, tiamulin; TIL, tilmicosin; TIO, ceftiofur; TUL, tulathromycin; Tx, treatment; TYLT, tylosin tartrate.

Supplementary Figure 1.3.15 Unadjusted *Mannheimia haemolytica* normalized MIC distributions stratified by cattle type clustered by BRD-related mortality

A) Beef cattle

B) Dairy cattle

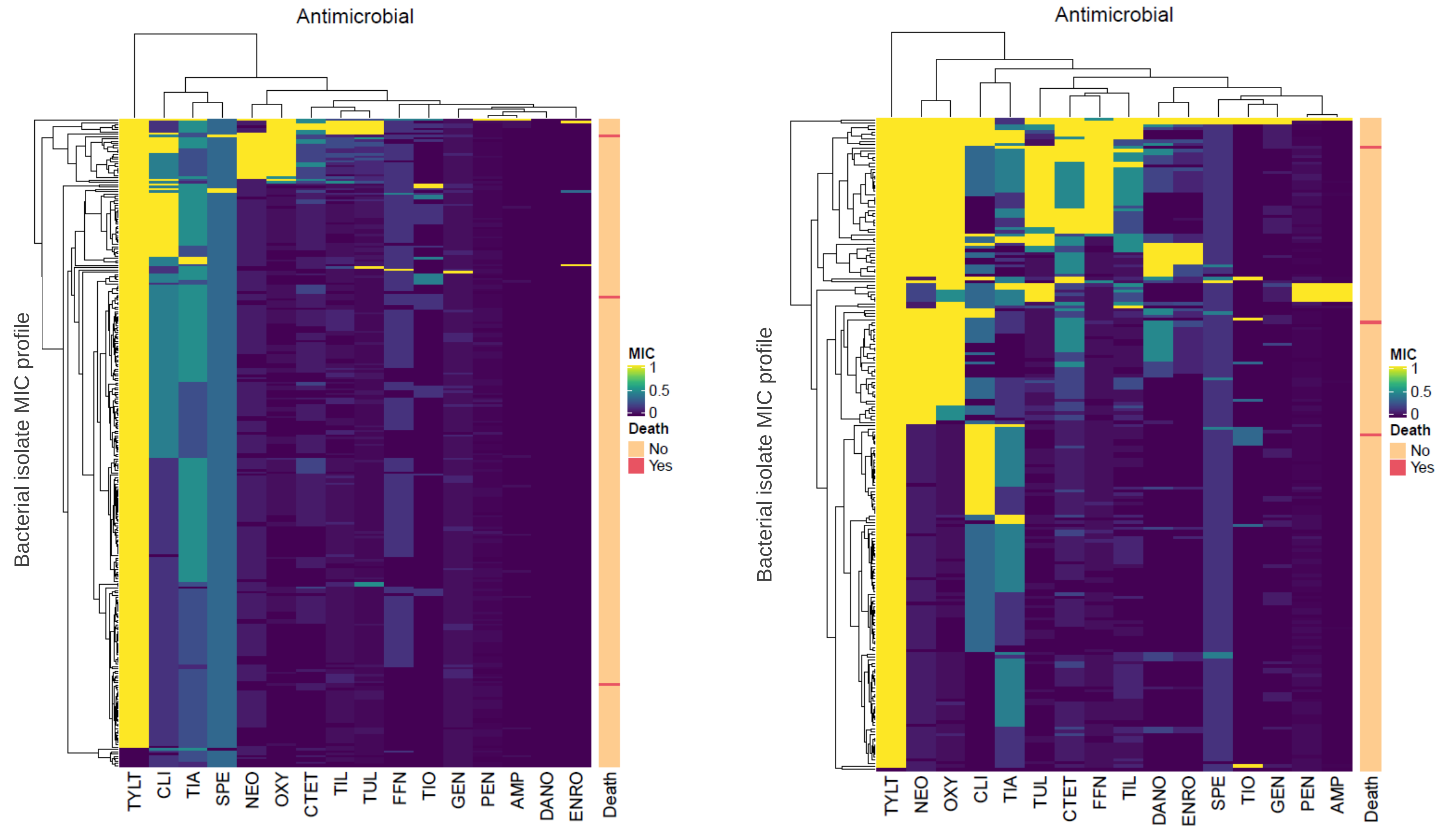

AMP, ampicillin; CLI, clindamycin; CTET, chlortetracycline; DANO, danofloxacin; ENRO, enrofloxacin; FFN, florfenicol; GEN, gentamycin; MIC, minimum inhibitory concentration; NEO, neomycin; OXY, oxytetracycline; PEN, penicillin; SPE, spectinomycin; TIA, tiamulin; TIL, tilmicosin; TIO, ceftiofur; TUL, tulathromycin; TYLT, tylosin tartrate.

Supplementary Figure 1.3.16 Unadjusted *Mannheimia haemolytica* normalized MIC distributions stratified by cattle type and clustered by feedlot

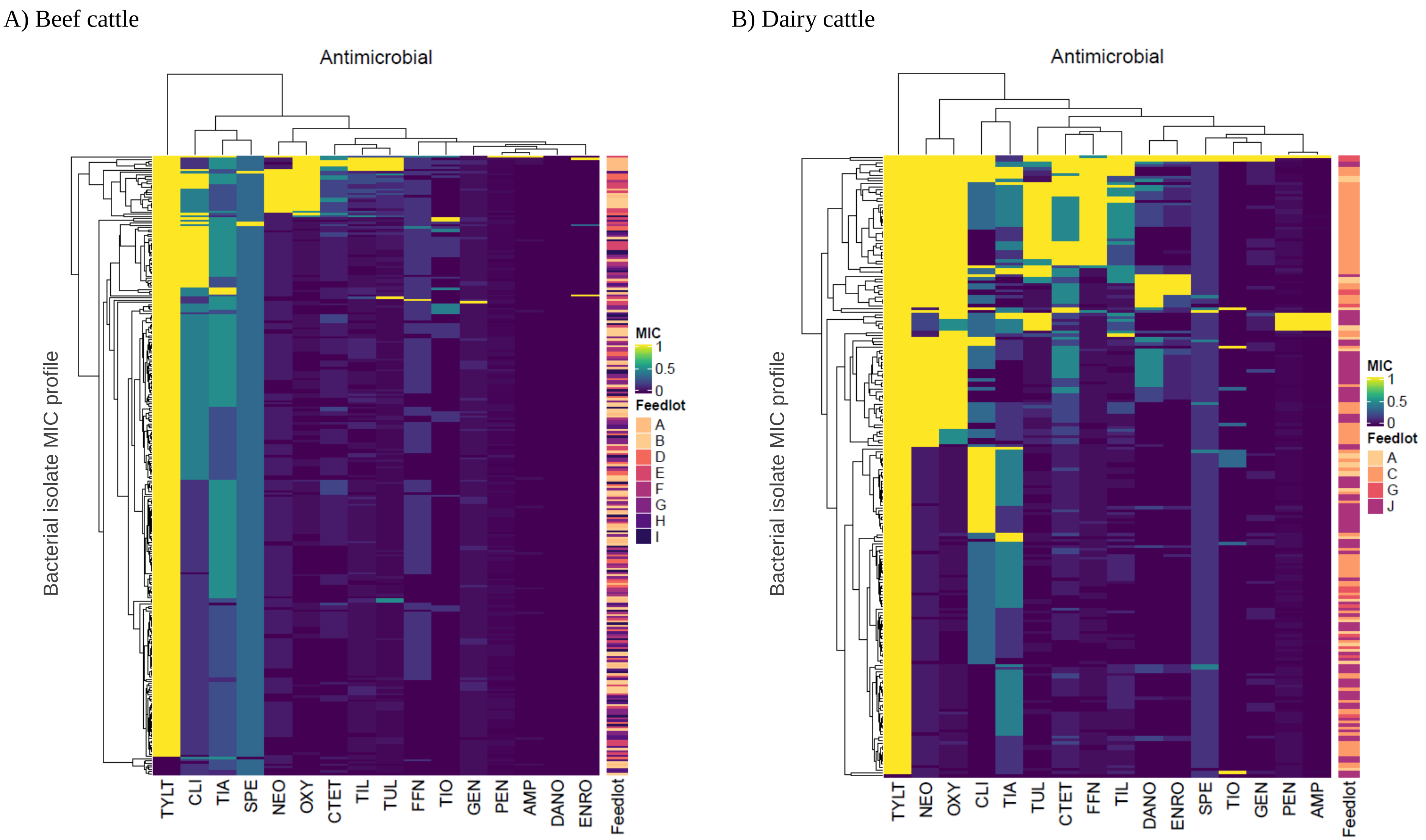

AMP, ampicillin; CLI, clindamycin; CTET, chlortetracycline; DANO, danofloxacin; ENRO, enrofloxacin; FFN, florfenicol; GEN, gentamycin; MIC, minimum inhibitory concentration; NEO, neomycin; OXY, oxytetracycline; PEN, penicillin; SPE, spectinomycin; TIA, tiamulin; TIL, tilmicosin; TIO, ceftiofur; TUL, tulathromycin; TYLT, tylosin tartrate.

Supplementary Figure 1.3.17 Unadjusted *Pasteurella multocida* normalized MIC distributions stratified by cattle type and clustered by country of origin

A) Beef cattle

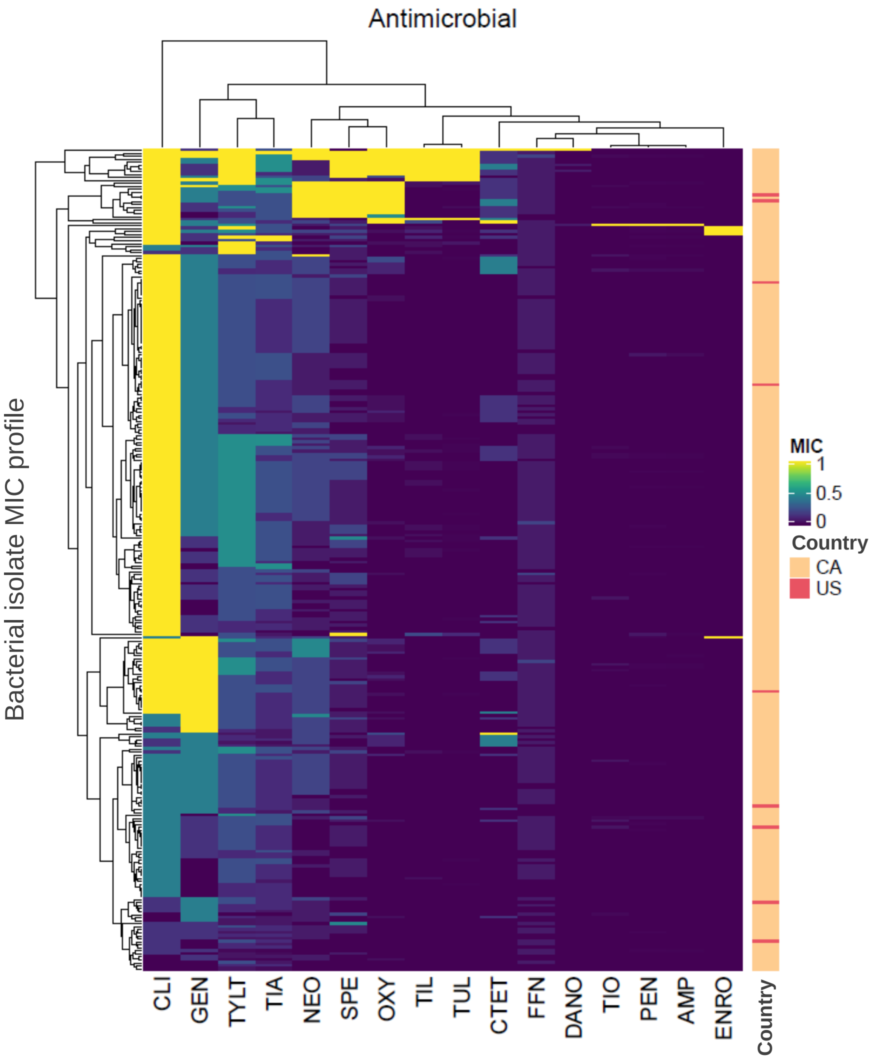

B) Dairy cattle

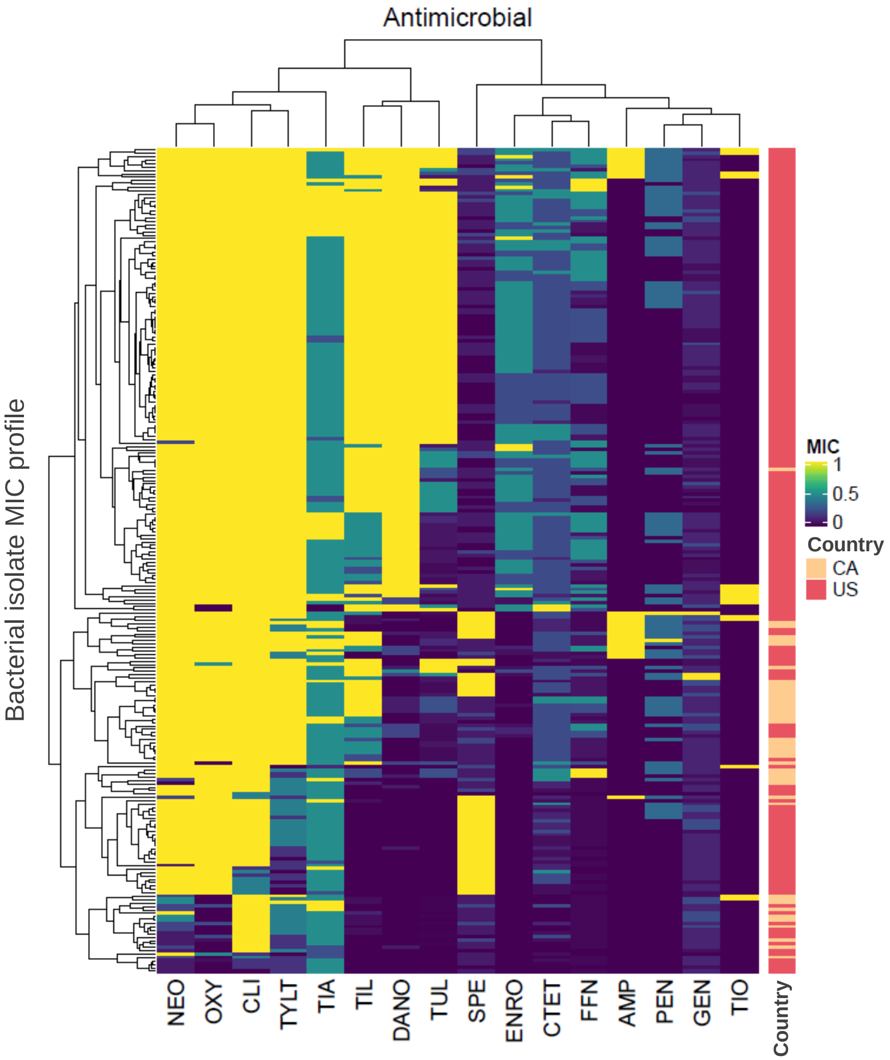

AMP, ampicillin; CA, Canada; CLI, clindamycin; CTET, chlortetracycline; DANO, danofloxacin; ENRO, enrofloxacin; FFN, florfenicol; GEN, gentamycin; MIC, minimum inhibitory concentration; NEO, neomycin; OXY, oxytetracycline; PEN, penicillin; SPE, spectinomycin; TIA, tiamulin; TIL, tilmicosin; TIO, ceftiofur; TUL, tulathromycin; TYLT, tylosin tartrate.

Supplementary Figure 1.3.18 Unadjusted *Pasteurella multocida* normalized MIC distributions stratified by cattle type and clustered by monthly interval

A) Beef cattle B) Dairy cattle

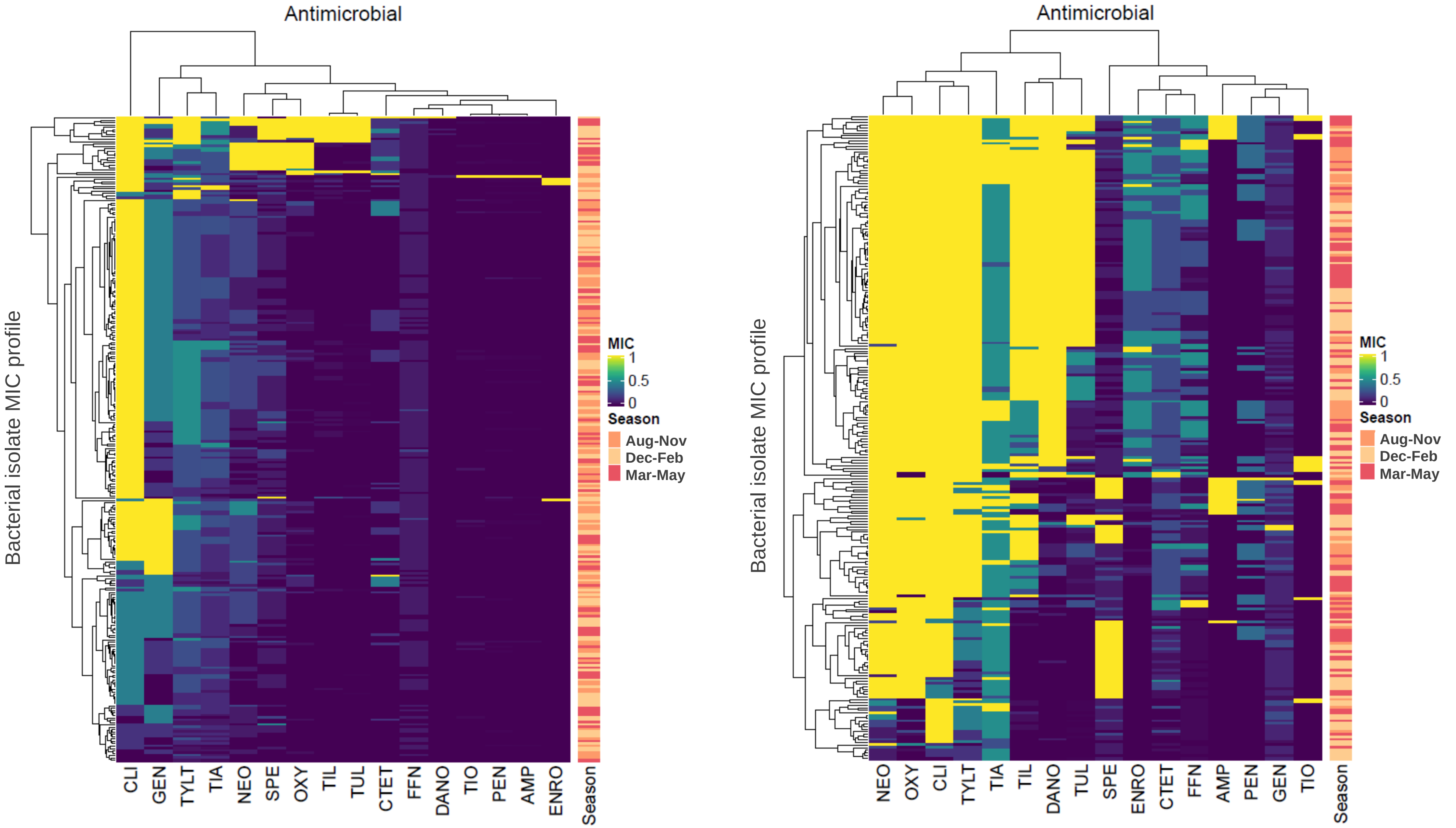

AMP, ampicillin; CLI, clindamycin; CTET, chlortetracycline; DANO, danofloxacin; ENRO, enrofloxacin; FFN, florfenicol; GEN, gentamycin; MIC, minimum inhibitory concentration; NEO, neomycin; OXY, oxytetracycline; PEN, penicillin; SPE, spectinomycin; TIA, tiamulin; TIL, tilmicosin; TIO, ceftiofur; TUL, tulathromycin; TYLT, tylosin tartrate.

Supplementary Figure 1.3.19 Unadjusted *Pasteurella multocida* normalized MIC distributions stratified by cattle type and clustered by source

A) Beef cattle B) Dairy cattle

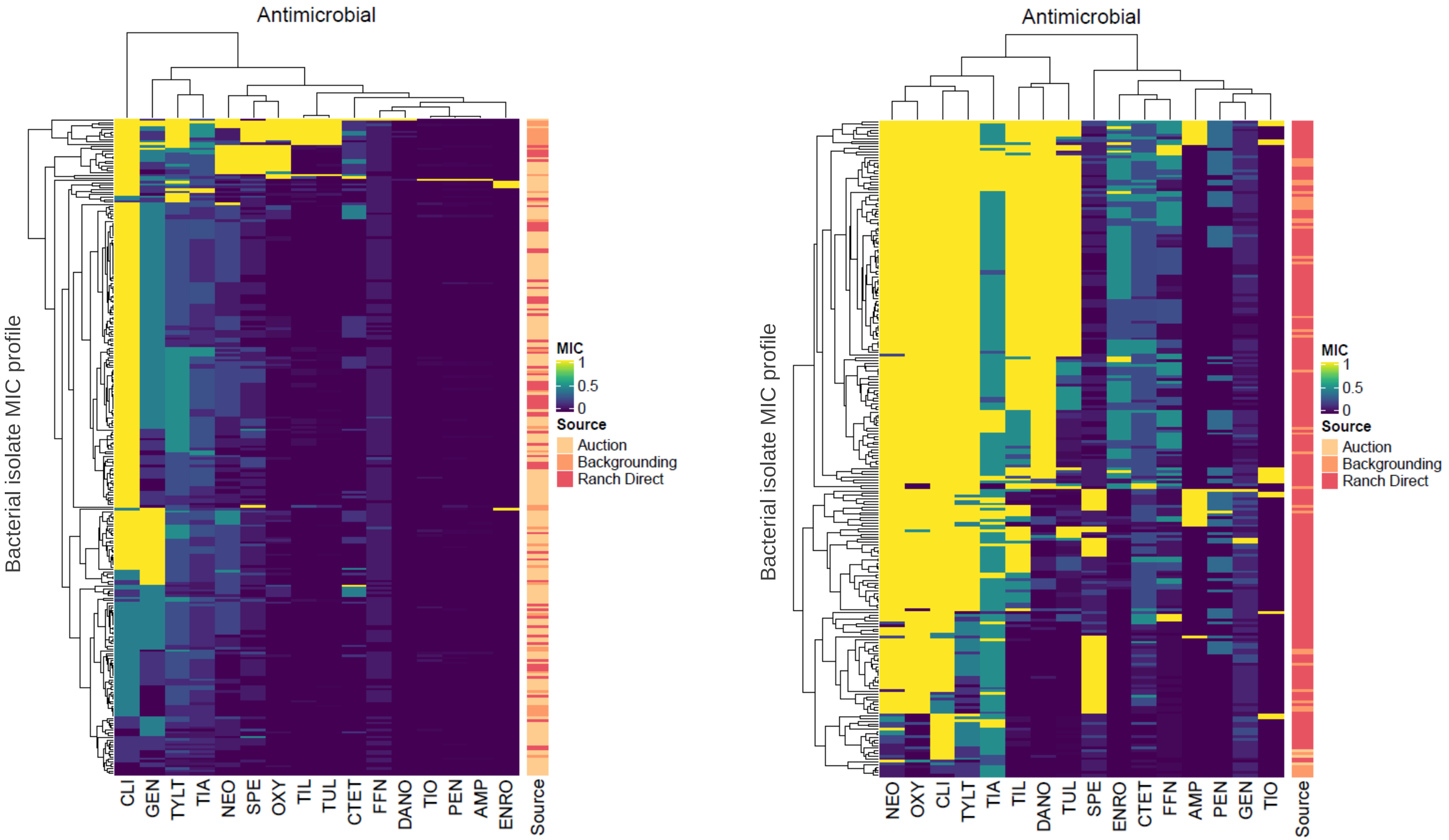

AMP, ampicillin; Backgrounding operations; CLI, clindamycin; CTET, chlortetracycline; DANO, danofloxacin; ENRO, enrofloxacin; FFN, florfenicol; GEN, gentamycin; MIC, minimum inhibitory concentration; NEO, neomycin; OXY, oxytetracycline; PEN, penicillin; SPE, spectinomycin; TIA, tiamulin; TIL, tilmicosin; TIO, ceftiofur; TUL, tulathromycin; TYLT, tylosin tartrate.

Supplementary Figure 1.3.20 Unadjusted *Pasteurella multocida* normalized MIC distributions stratified by cattle type and clustered by risk of suffering BRD

A) Beef cattle

B) Dairy cattle

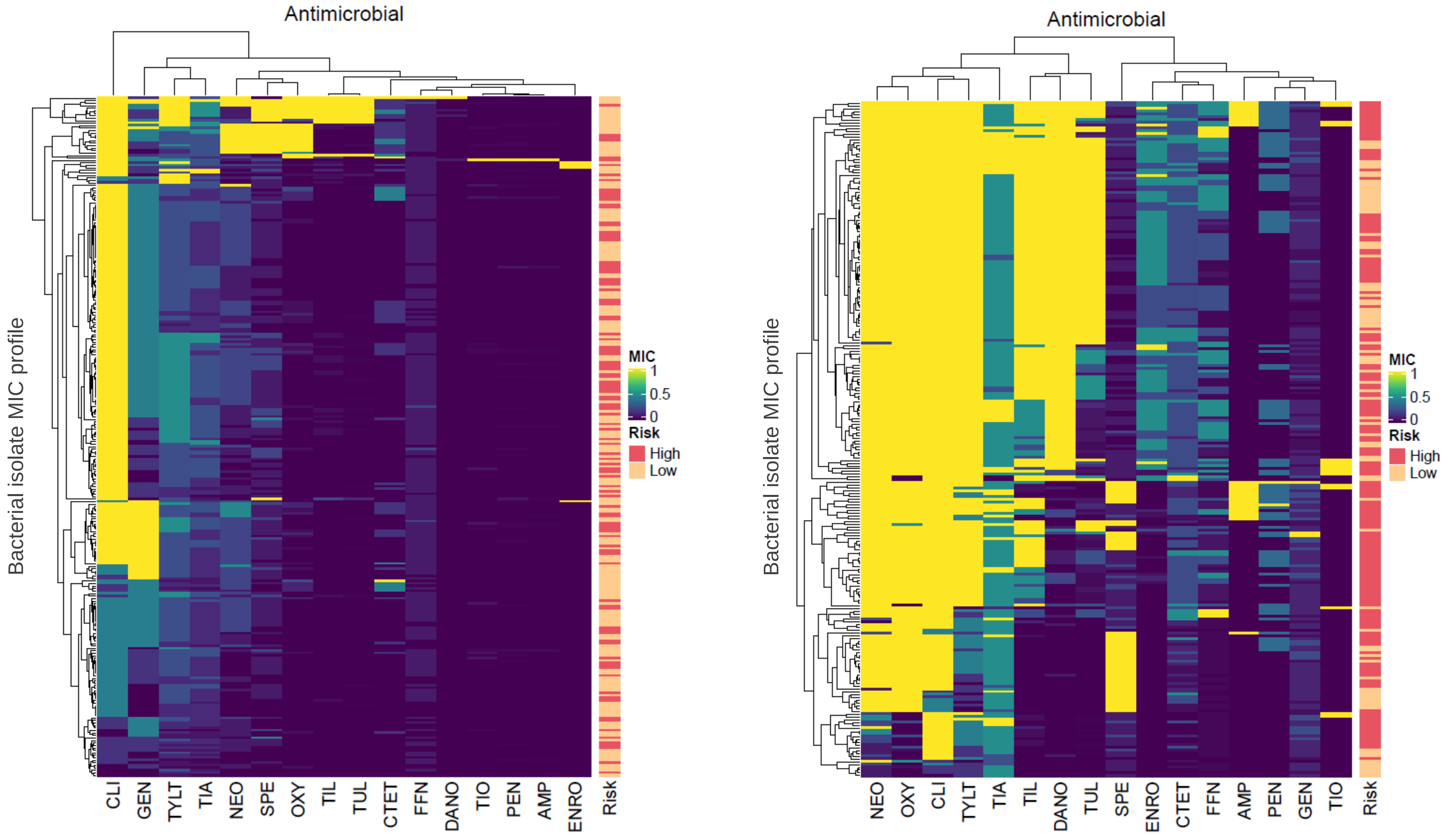

AMP, ampicillin; CLI, clindamycin; CTET, chlortetracycline; DANO, danofloxacin; ENRO, enrofloxacin; FFN, florfenicol; GEN, gentamycin; MIC, minimum inhibitory concentration; NEO, neomycin; OXY, oxytetracycline; PEN, penicillin; SPE, spectinomycin; TIA, tiamulin; TIL, tilmicosin; TIO, ceftiofur; TUL, tulathromycin; TYLT, tylosin tartrate.

Supplementary Figure 1.3.21 Unadjusted *Pasteurella multocida* normalized MIC distributions stratified by cattle type and clustered by weight range

A) Beef cattle

B) Dairy cattle

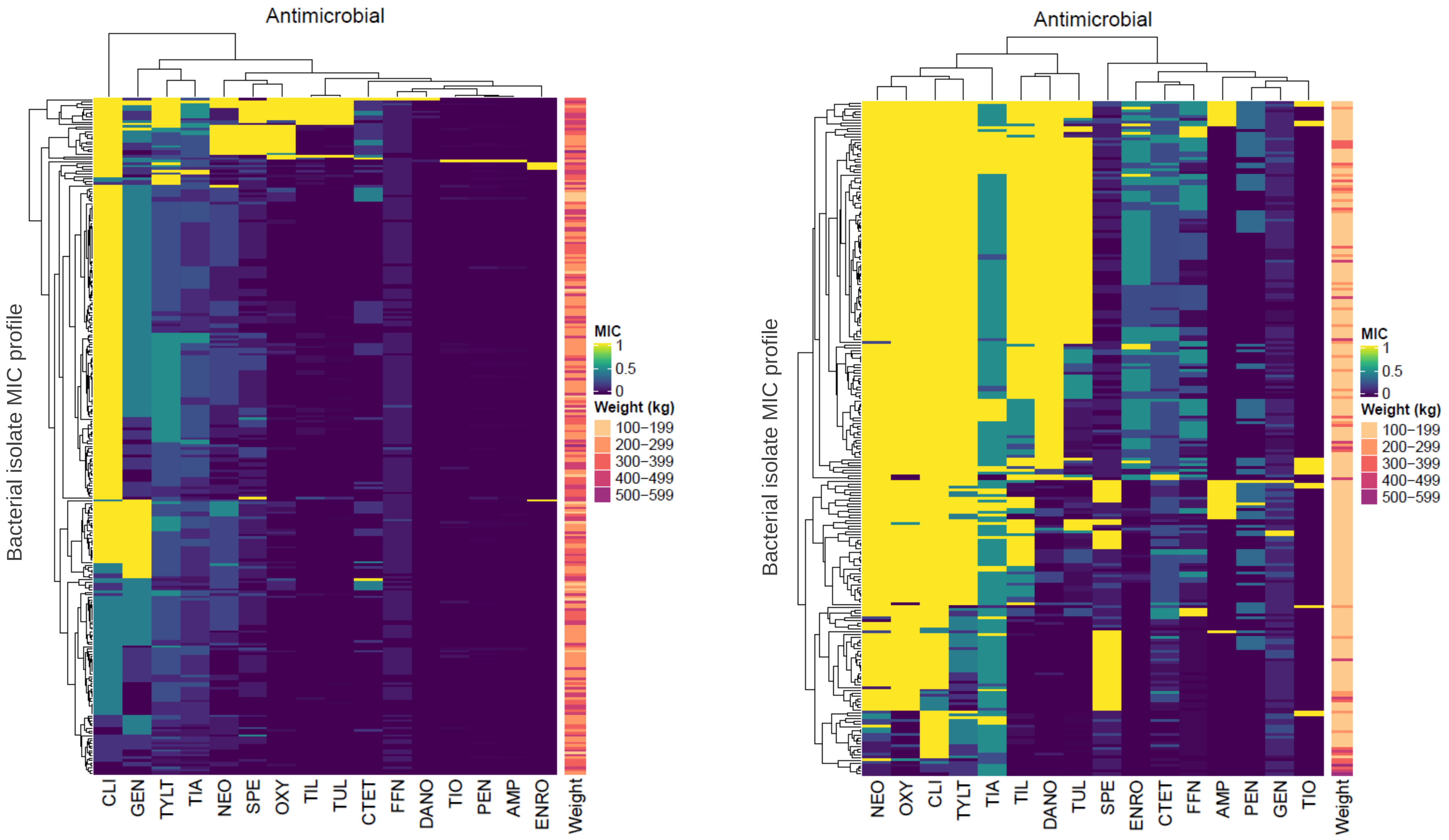

AMP, ampicillin; CLI, clindamycin; CTET, chlortetracycline; DANO, danofloxacin; ENRO, enrofloxacin; FFN, florfenicol; GEN, gentamycin; MIC, minimum inhibitory concentration; NEO, neomycin; OXY, oxytetracycline; PEN, penicillin; SPE, spectinomycin; TIA, tiamulin; TIL, tilmicosin; TIO, ceftiofur; TUL, tulathromycin; TYLT, tylosin tartrate.

Supplementary Figure 1.3.22 Unadjusted *Pasteurella multocida* normalized MIC distributions stratified by cattle type and clustered by sex

A) Beef cattle

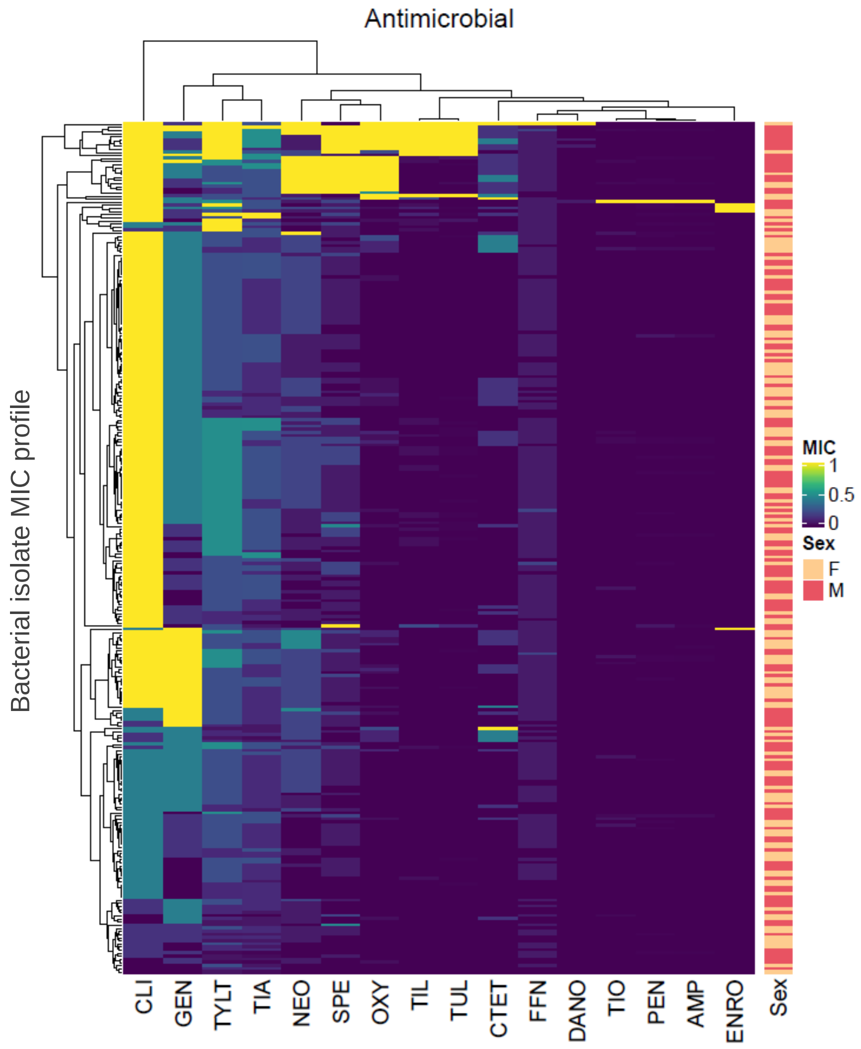

B) Dairy cattle

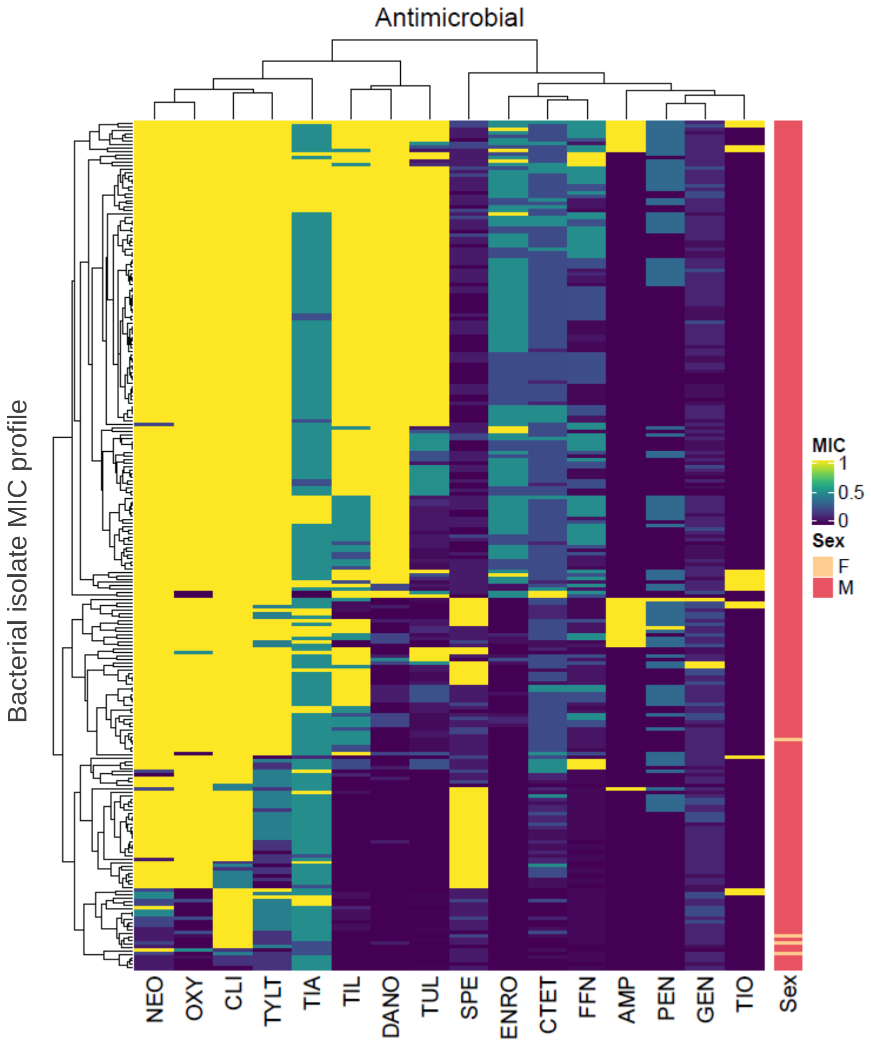

AMP, ampicillin; CLI, clindamycin; CTET, chlortetracycline; DANO, danofloxacin; ENRO, enrofloxacin; F, female; FFN, florfenicol; GEN, gentamycin; M, male; MIC, minimum inhibitory concentration; NEO, neomycin; OXY, oxytetracycline; PEN, penicillin; SPE, spectinomycin; TIA, tiamulin; TIL, tilmicosin; TIO, ceftiofur; TUL, tulathromycin; TYLT, tylosin tartrate.

Supplementary Figure 1.3.23 Unadjusted *Pasteurella multocida* normalized MIC distributions stratified by cattle type and clustered by age

A) Beef cattle

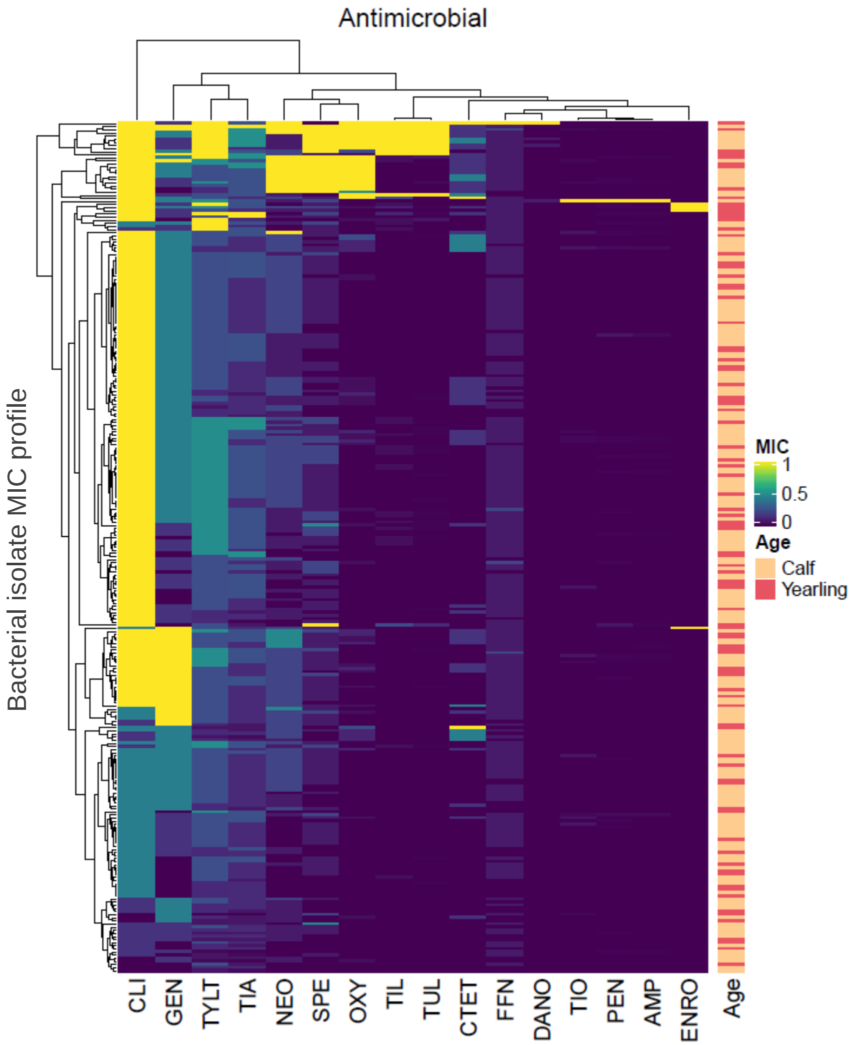

B) Dairy cattle

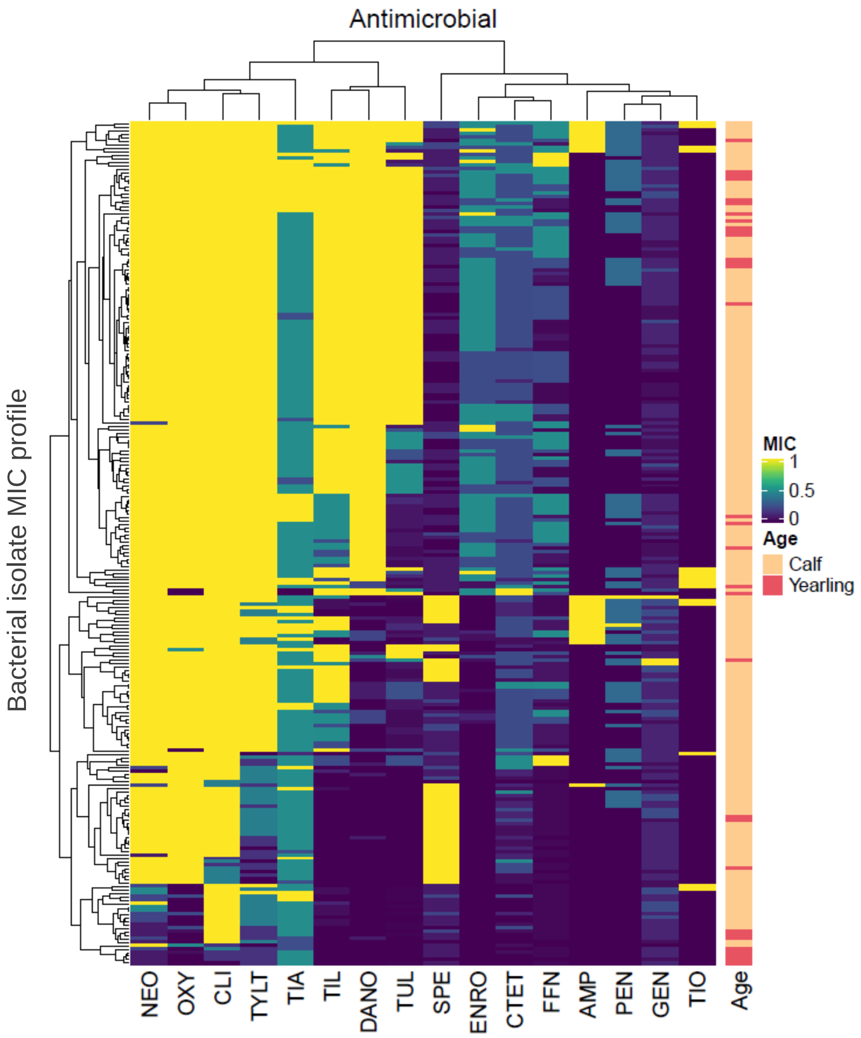

AMP, ampicillin; CLI, clindamycin; CTET, chlortetracycline; DANO, danofloxacin; ENRO, enrofloxacin; FFN, florfenicol; GEN, gentamycin; MIC, minimum inhibitory concentration; NEO, neomycin; OXY, oxytetracycline; PEN, penicillin; SPE, spectinomycin; TIA, tiamulin; TIL, tilmicosin; TIO, ceftiofur; TUL, tulathromycin; TYLT, tylosin tartrate.

Supplementary Figure 1.3.24 Unadjusted *Pasteurella multocida* normalized MIC distributions stratified by cattle type and clustered by temperature range

A) Beef cattle B) Dairy cattle

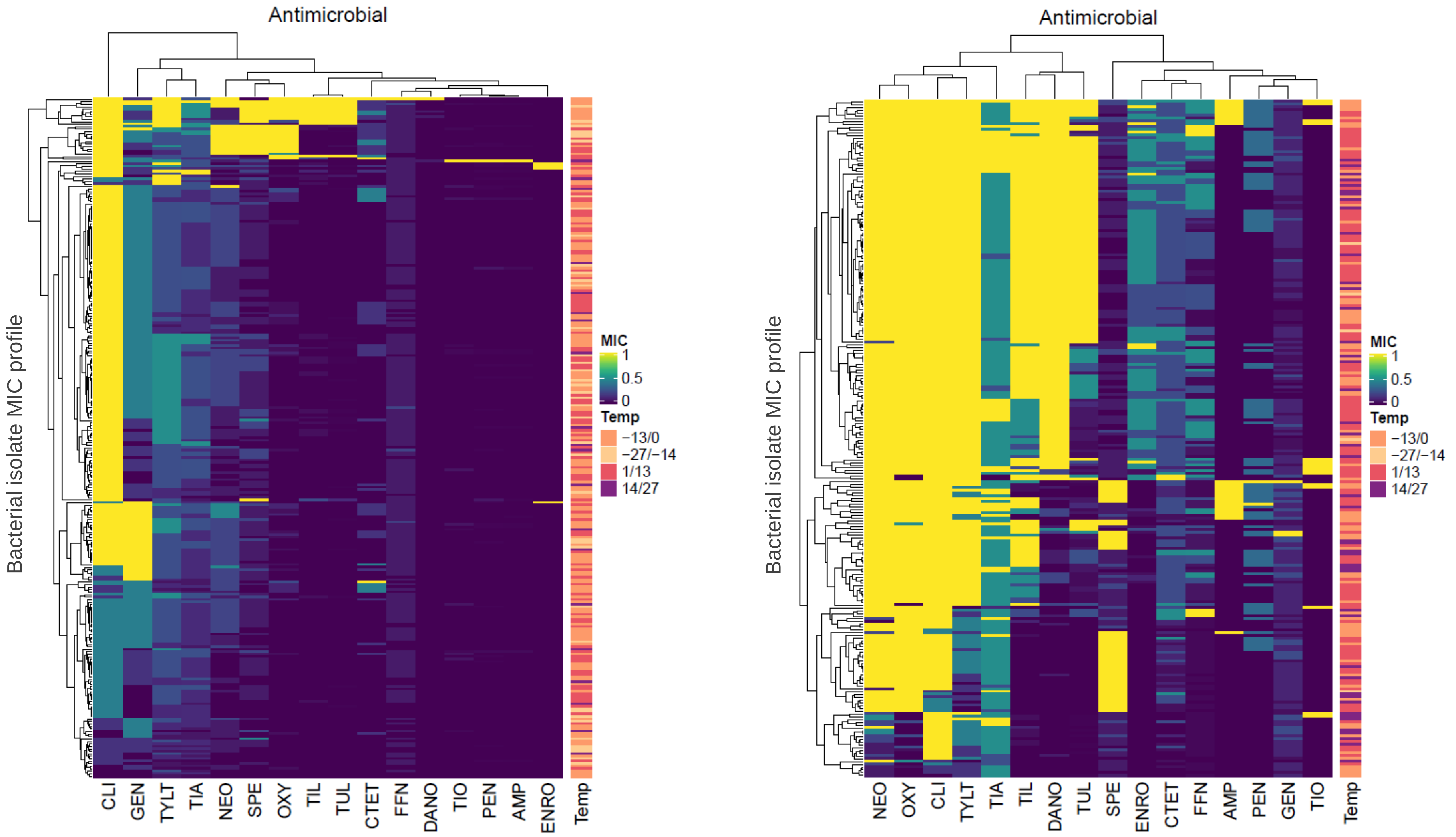

AMP, ampicillin; CLI, clindamycin; CTET, chlortetracycline; DANO, danofloxacin; ENRO, enrofloxacin; FFN, florfenicol; GEN, gentamycin; MIC, minimum inhibitory concentration; NEO, neomycin; OXY, oxytetracycline; PEN, penicillin; SPE, spectinomycin; Temp, temperature range (°C); TIA, tiamulin; TIL, tilmicosin; TIO, ceftiofur; TUL, tulathromycin; TYLT, tylosin tartrate.

Supplementary Figure 1.3.25 Unadjusted *Pasteurella multocida* normalized MIC distributions stratified by cattle type and clustered by BRD-related morbidity

A) Beef cattle

B) Dairy cattle

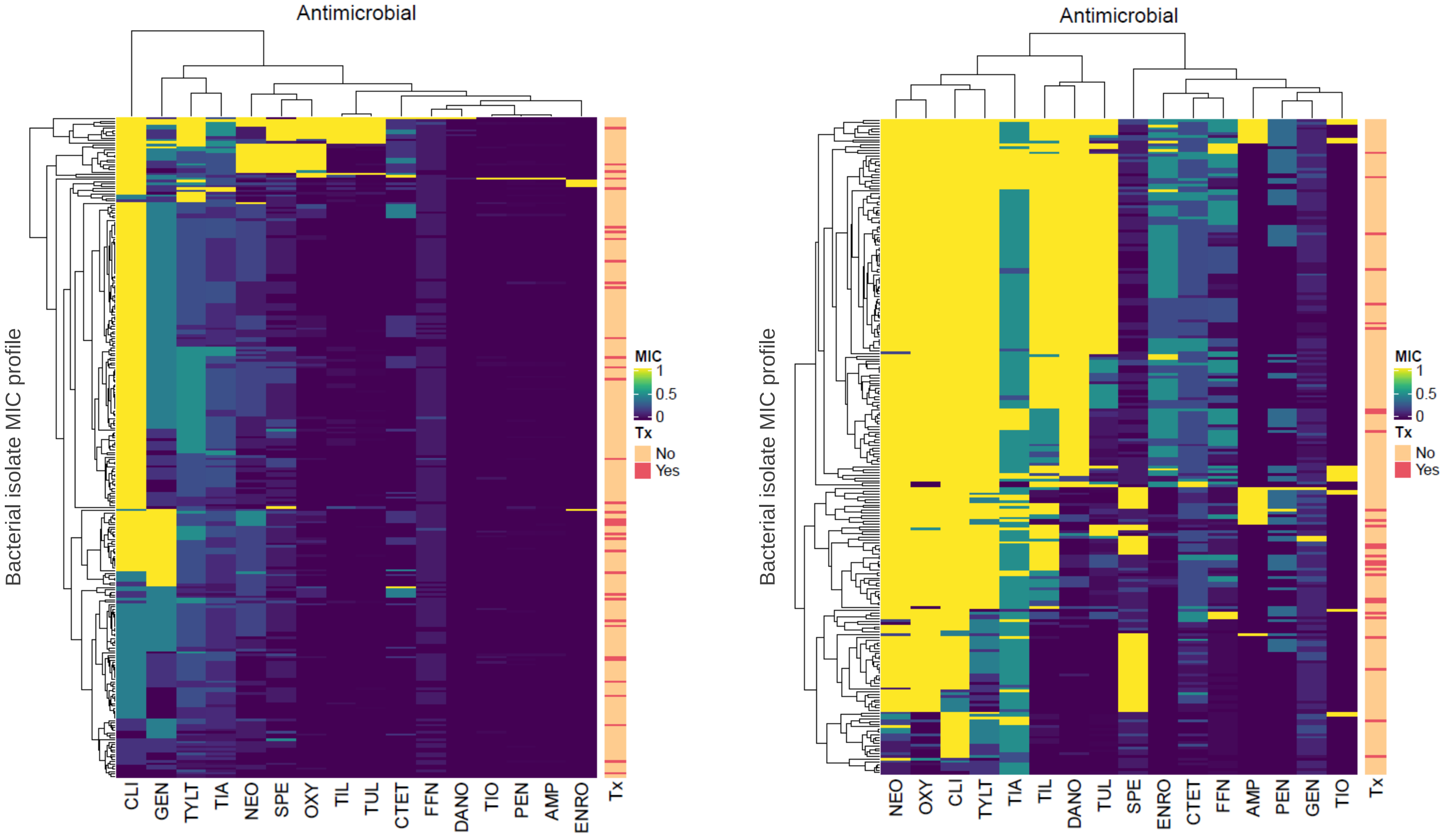

AMP, ampicillin; CLI, clindamycin; CTET, chlortetracycline; DANO, danofloxacin; ENRO, enrofloxacin; FFN, florfenicol; GEN, gentamycin; MIC, minimum inhibitory concentration; NEO, neomycin; OXY, oxytetracycline; PEN, penicillin; SPE, spectinomycin; TIA, tiamulin; TIL, tilmicosin; TIO, ceftiofur; TUL, tulathromycin; Tx, treatment; TYLT, tylosin tartrate.

Supplementary Figure 1.3.26 Unadjusted *Pasteurella multocida* normalized MIC distributions stratified by cattle type and clustered by BRD-related mortality

A) Beef cattle B) Dairy cattle

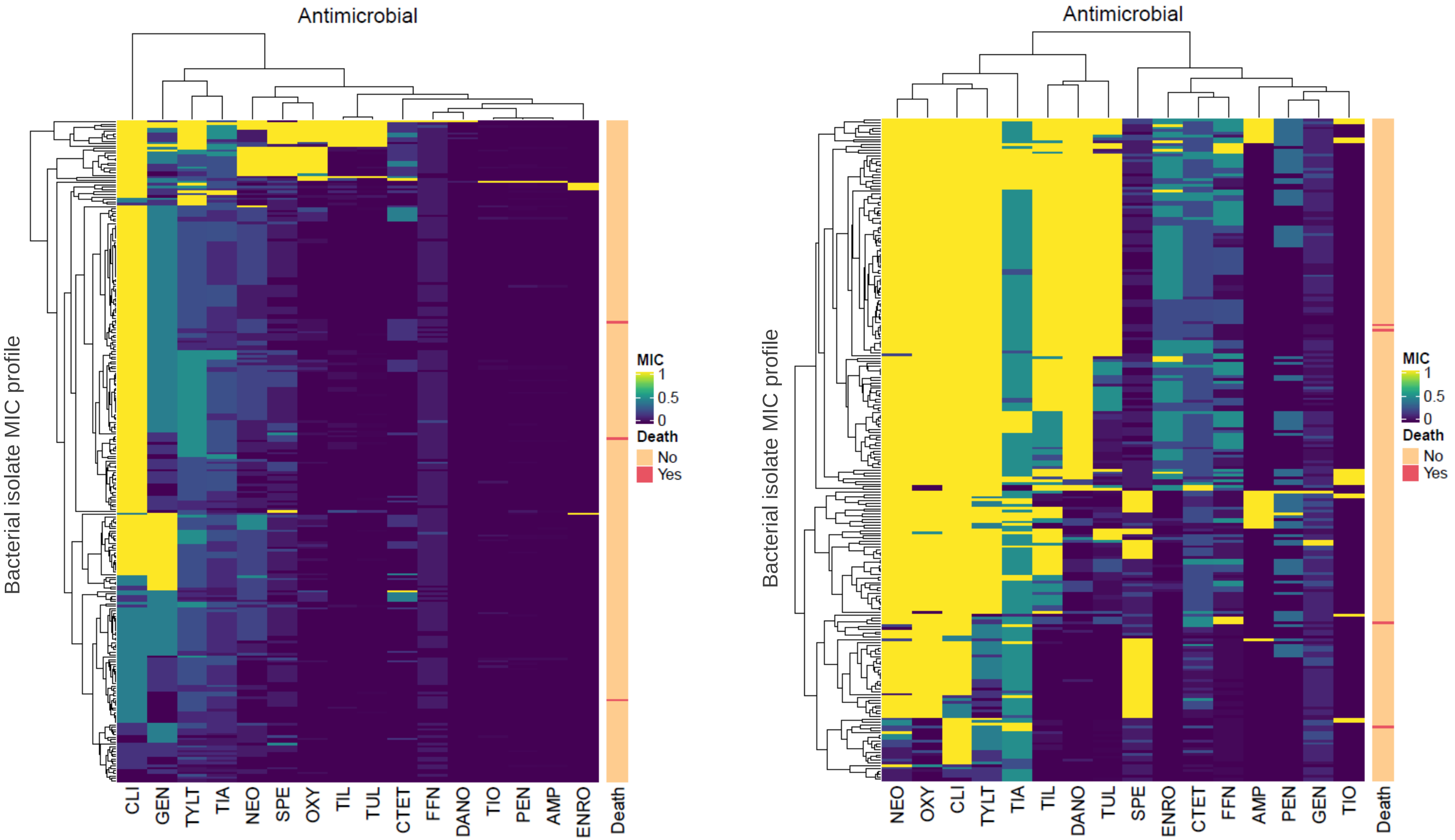

AMP, ampicillin; CLI, clindamycin; CTET, chlortetracycline; DANO, danofloxacin; ENRO, enrofloxacin; FFN, florfenicol; GEN, gentamycin; MIC, minimum inhibitory concentration; NEO, neomycin; OXY, oxytetracycline; PEN, penicillin; SPE, spectinomycin; TIA, tiamulin; TIL, tilmicosin; TIO, ceftiofur; TUL, tulathromycin; TYLT, tylosin tartrate.

Supplementary Figure 1.3.27 Unadjusted *Pasteurella multocida* normalized MIC distributions stratified by cattle type and clustered by feedlot

A) Beef cattle

B) Dairy cattle

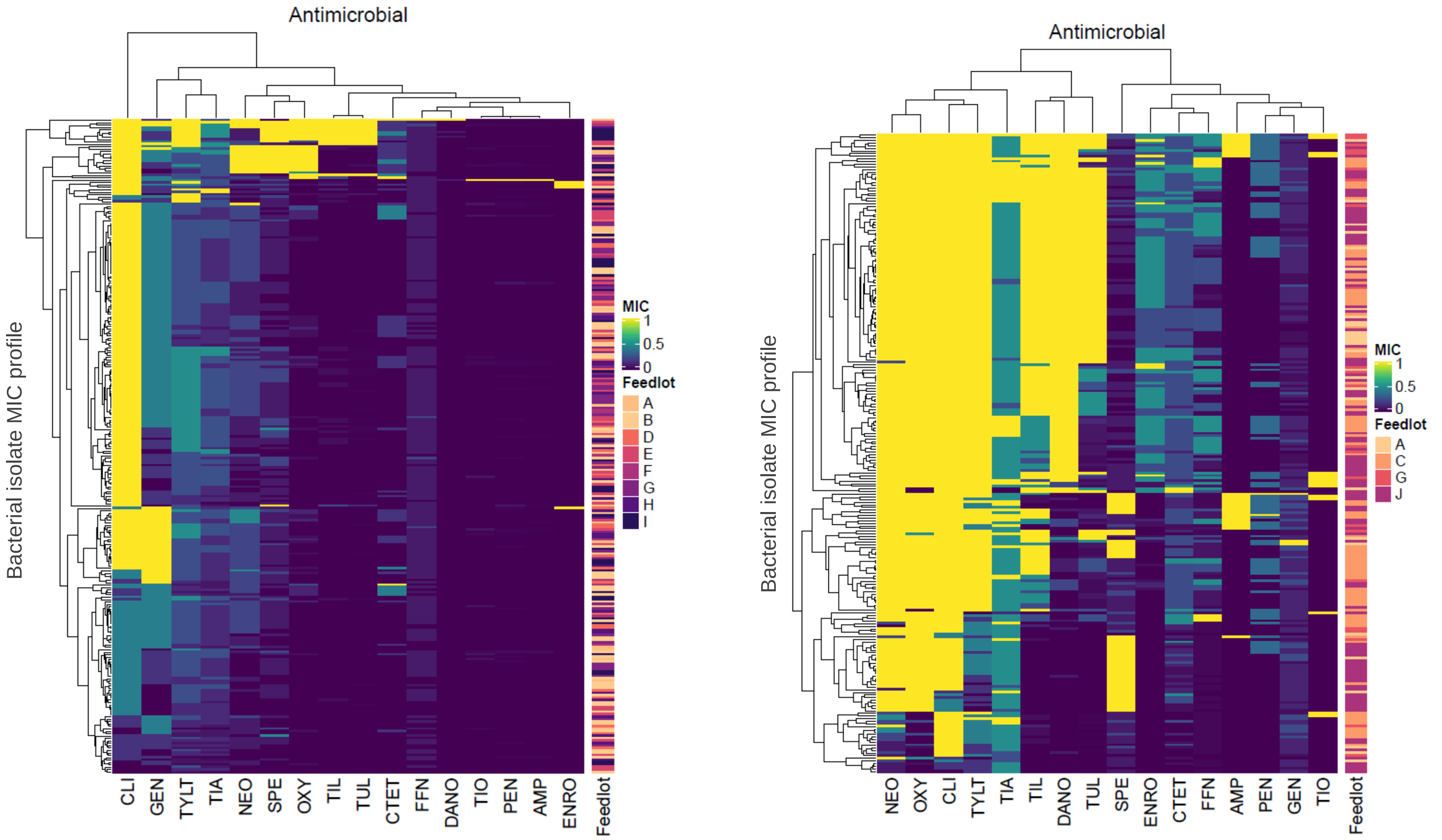

AMP, ampicillin; CLI, clindamycin; CTET, chlortetracycline; DANO, danofloxacin; ENRO, enrofloxacin; FFN, florfenicol; GEN, gentamycin; MIC, minimum inhibitory concentration; NEO, neomycin; OXY, oxytetracycline; PEN, penicillin; SPE, spectinomycin; TIA, tiamulin; TIL, tilmicosin; TIO, ceftiofur; TUL, tulathromycin; TYLT, tylosin tartrate.

Supplementary Figure 1.3.28 Unadjusted *Histophilus somni* normalized MIC distributions stratified by cattle type and clustered by country of origin

A) Beef cattle

B) Dairy cattle

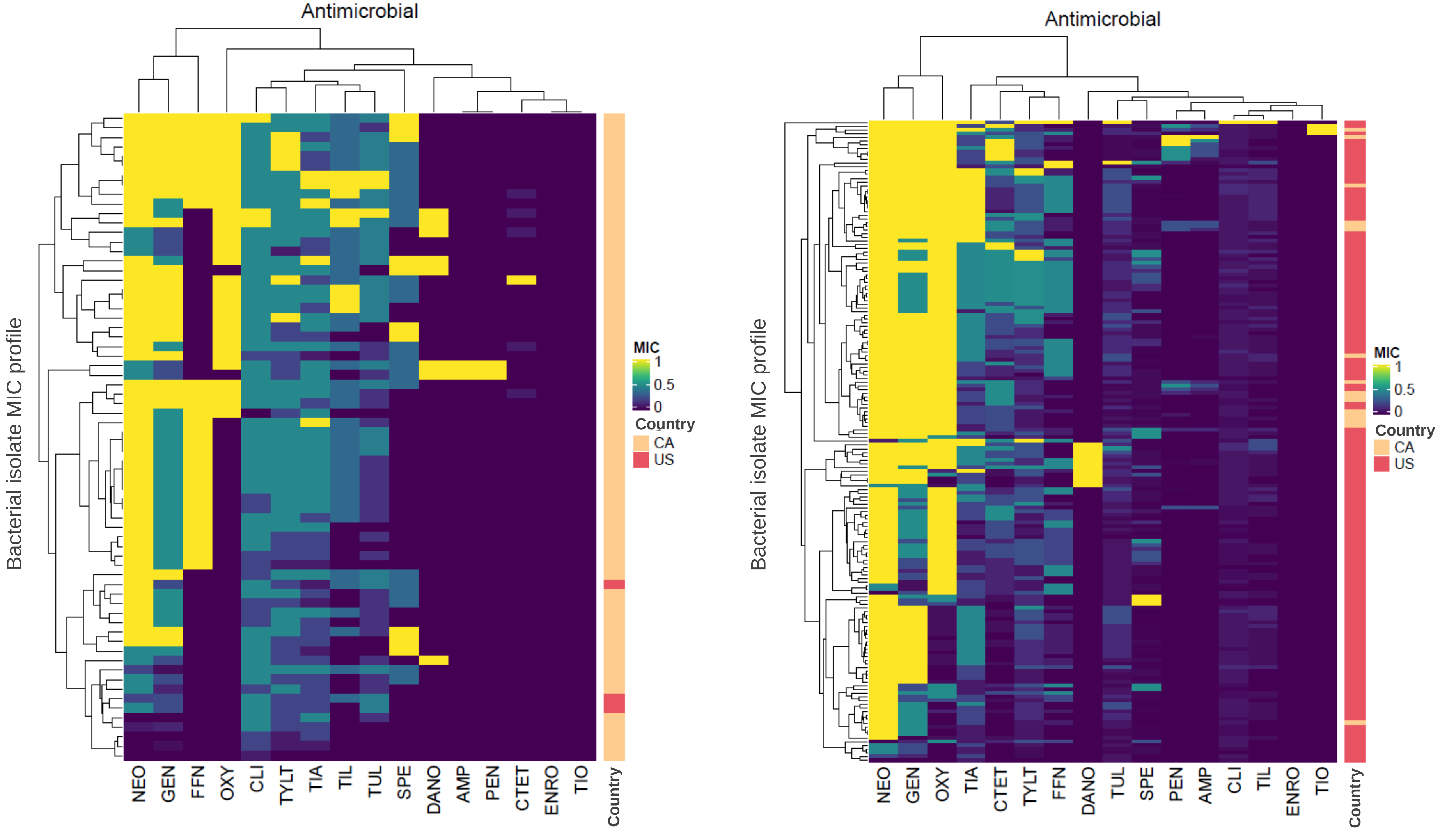

AMP, ampicillin; CA, Canada; CLI, clindamycin; CTET, chlortetracycline; DANO, danofloxacin; ENRO, enrofloxacin; FFN, florfenicol; GEN, gentamycin; MIC, minimum inhibitory concentration; NEO, neomycin; OXY, oxytetracycline; PEN, penicillin; SPE, spectinomycin; TIA, tiamulin; TIL, tilmicosin; TIO, ceftiofur; TUL, tulathromycin; TYLT, tylosin tartrate.

Supplementary Figure 1.3.29 Unadjusted *Histophilus somni* normalized MIC distributions stratified by cattle type and clustered by monthly interval

A) Beef cattle

B) Dairy cattle

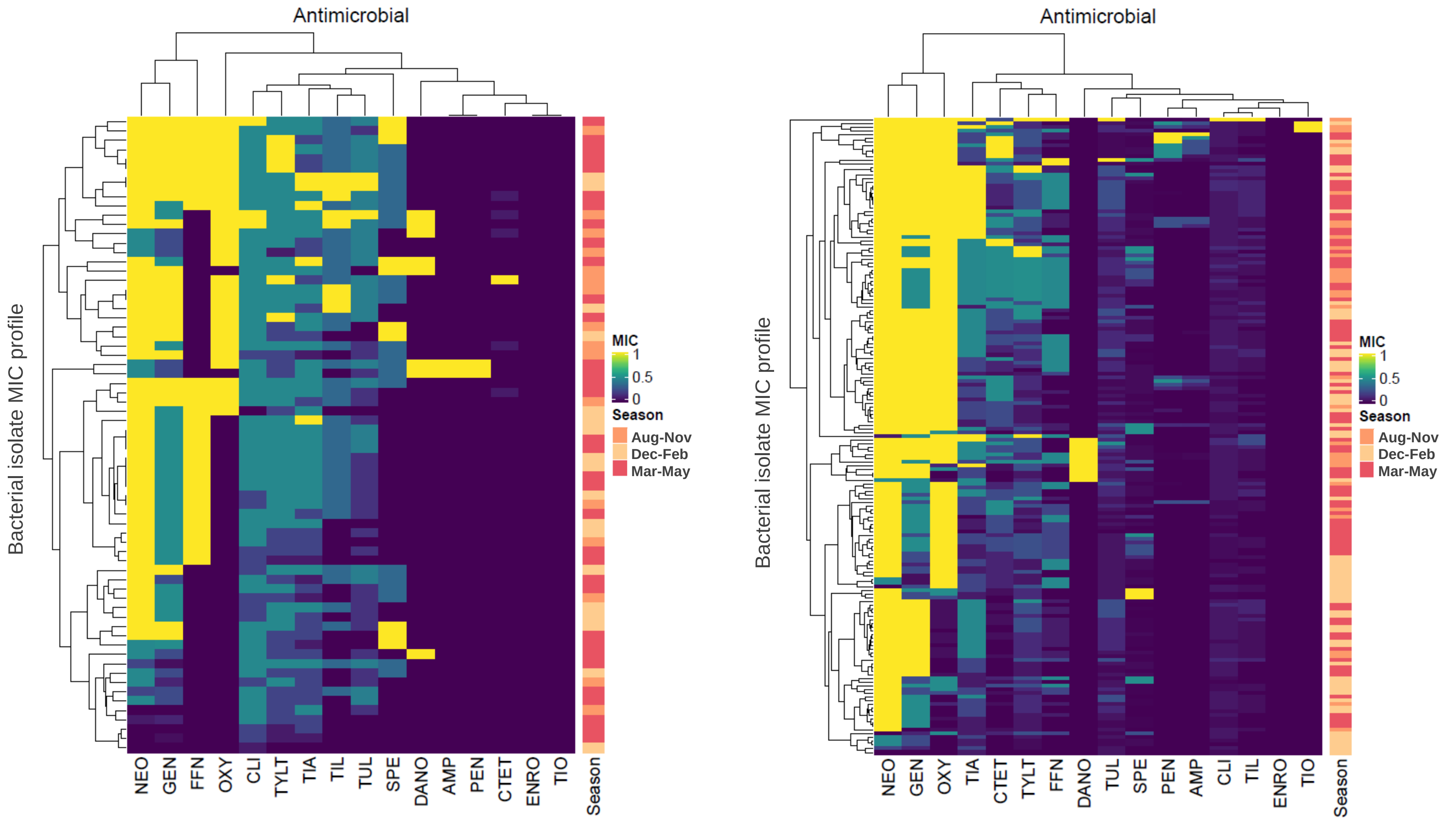

AMP, ampicillin; CLI, clindamycin; CTET, chlortetracycline; DANO, danofloxacin; ENRO, enrofloxacin; FFN, florfenicol; GEN, gentamycin; MIC, minimum inhibitory concentration; NEO, neomycin; OXY, oxytetracycline; PEN, penicillin; SPE, spectinomycin; TIA, tiamulin; TIL, tilmicosin; TIO, ceftiofur; TUL, tulathromycin; TYLT, tylosin tartrate.

Supplementary Figure 1.3.30 Unadjusted *Histophilus somni* normalized MIC distributions stratified by cattle type and clustered by source

A) Beef cattle

B) Dairy cattle

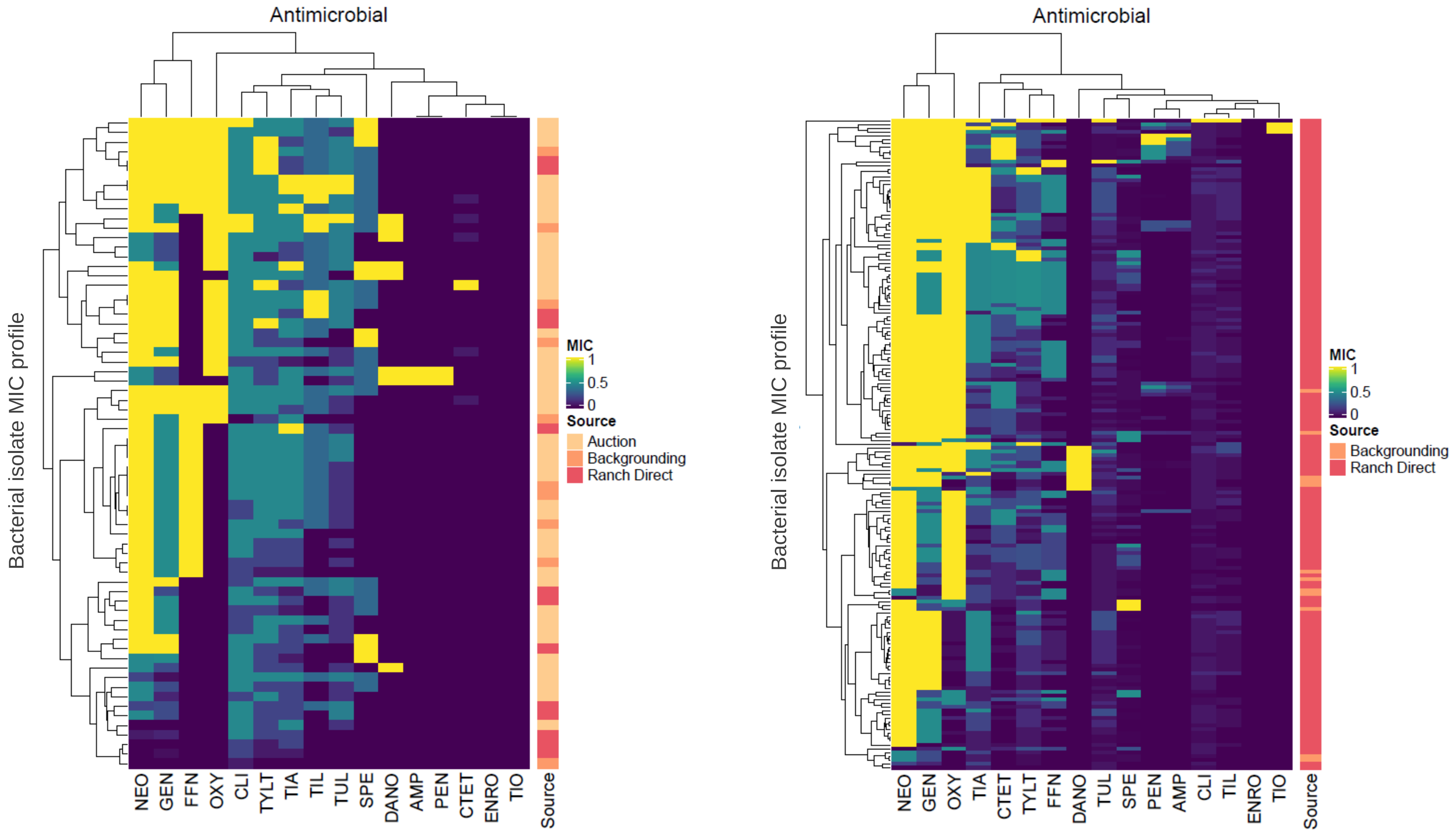

AMP, ampicillin; Backgrounding operations; CLI, clindamycin; CTET, chlortetracycline; DANO, danofloxacin; ENRO, enrofloxacin; FFN, florfenicol; GEN, gentamycin; MIC, minimum inhibitory concentration; NEO, neomycin; OXY, oxytetracycline; PEN, penicillin; SPE, spectinomycin; TIA, tiamulin; TIL, tilmicosin; TIO, ceftiofur; TUL, tulathromycin; TYLT, tylosin tartrate.

Supplementary Figure 1.3.31 Unadjusted *Histophilus somni* normalized MIC distributions stratified by cattle type and clustered by risk of suffering BRD

A) Beef cattle B) Dairy cattle

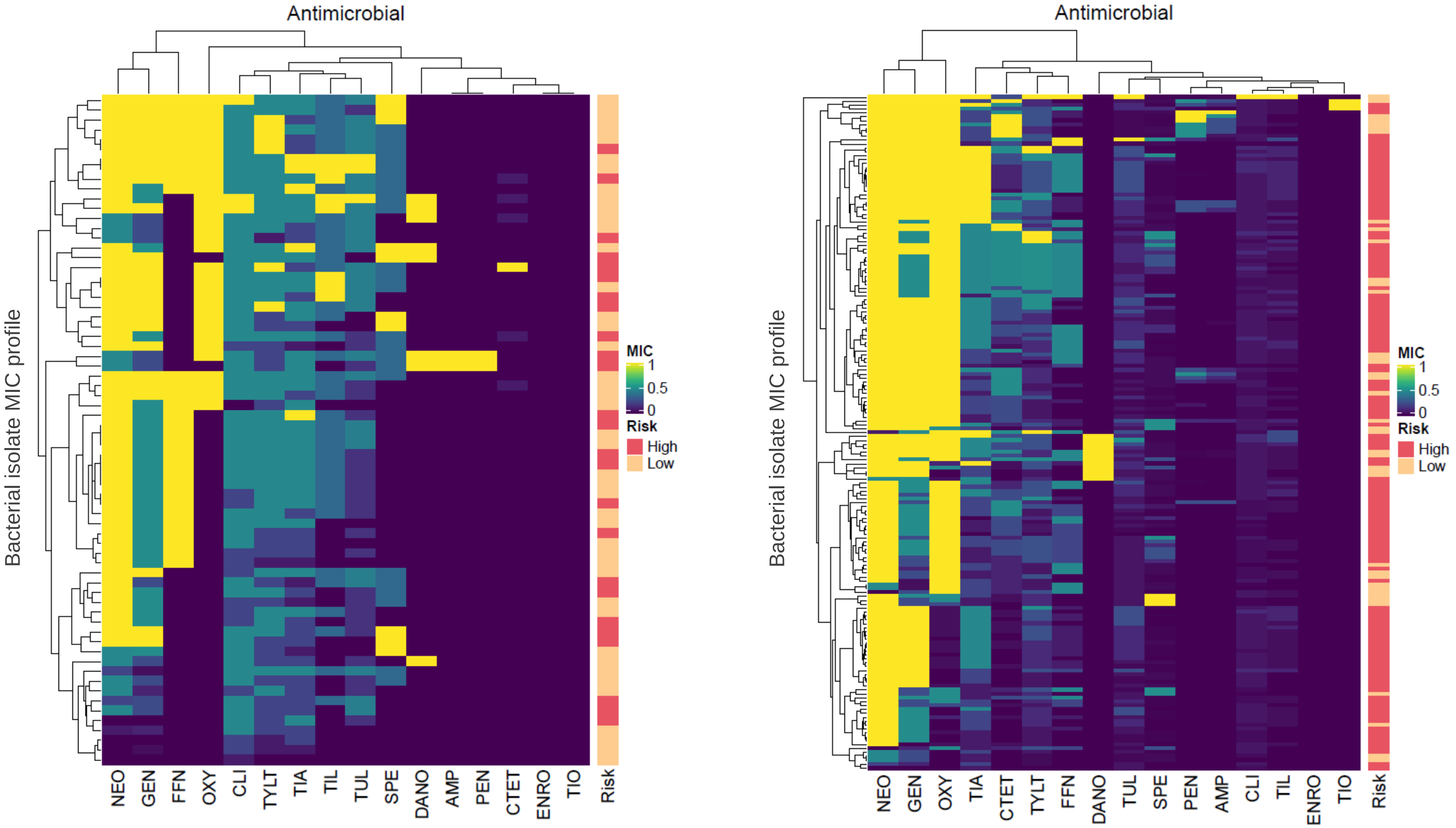

AMP, ampicillin; CLI, clindamycin; CTET, chlortetracycline; DANO, danofloxacin; ENRO, enrofloxacin; FFN, florfenicol; GEN, gentamycin; MIC, minimum inhibitory concentration; NEO, neomycin; OXY, oxytetracycline; PEN, penicillin; SPE, spectinomycin; TIA, tiamulin; TIL, tilmicosin; TIO, ceftiofur; TUL, tulathromycin; TYLT, tylosin tartrate.

Supplementary Figure 1.3.32 Unadjusted *Histophilus somni* normalized MIC distributions stratified by cattle type and clustered by weight range

A) Beef cattle

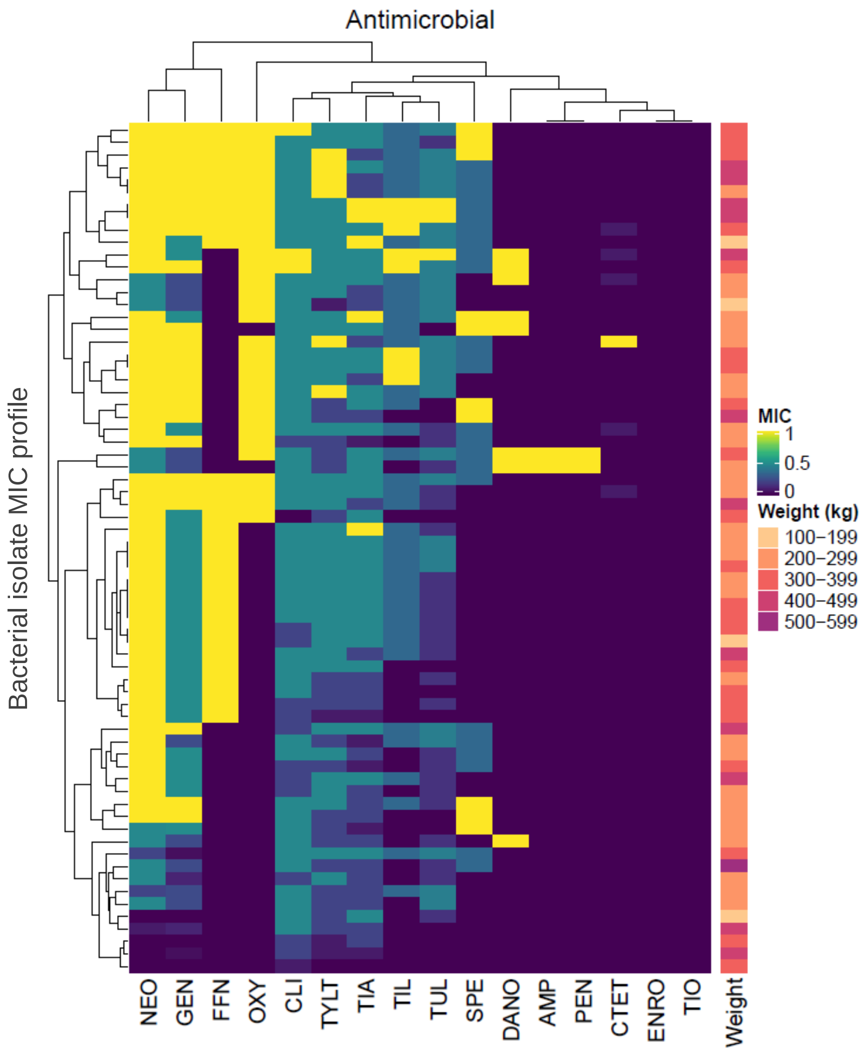

B) Dairy cattle

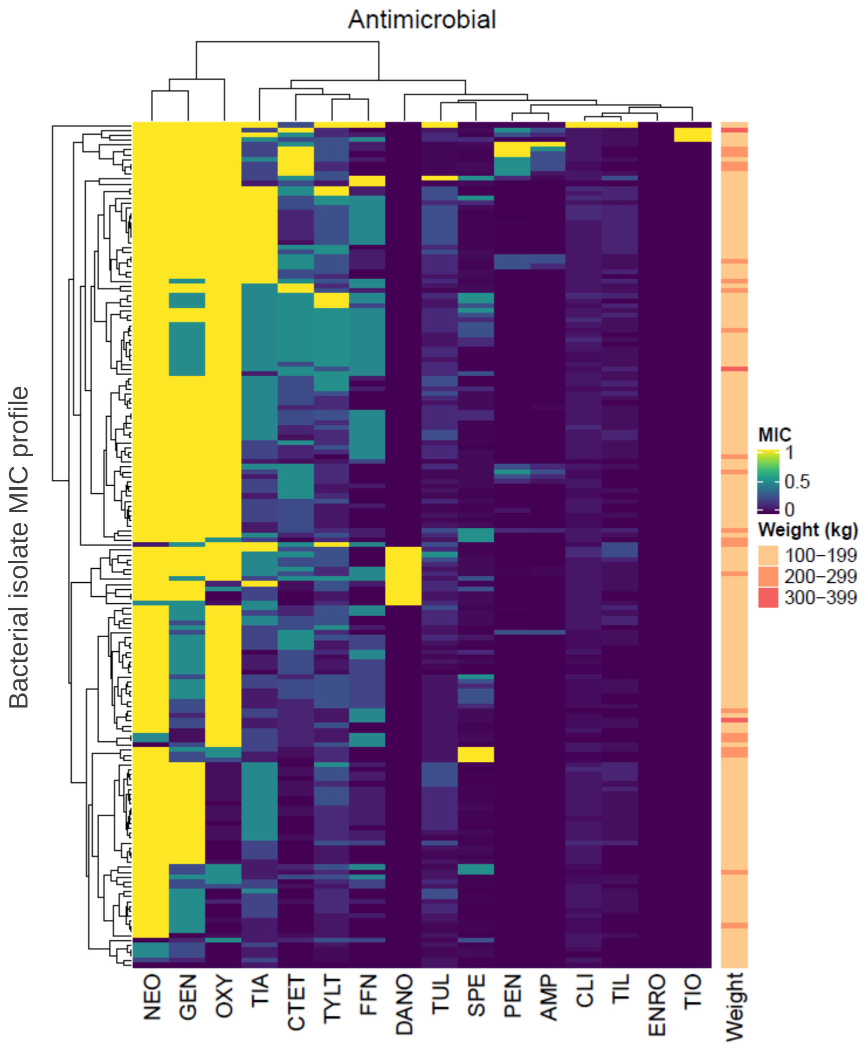

AMP, ampicillin; CLI, clindamycin; CTET, chlortetracycline; DANO, danofloxacin; ENRO, enrofloxacin; FFN, florfenicol; GEN, gentamycin; MIC, minimum inhibitory concentration; NEO, neomycin; OXY, oxytetracycline; PEN, penicillin; SPE, spectinomycin; TIA, tiamulin; TIL, tilmicosin; TIO, ceftiofur; TUL, tulathromycin; TYLT, tylosin tartrate.

Supplementary Figure 1.3.33 Unadjusted *Histophilus somni* normalized MIC distributions stratified by cattle type and clustered by sex

A) Beef cattle

B) Dairy cattle

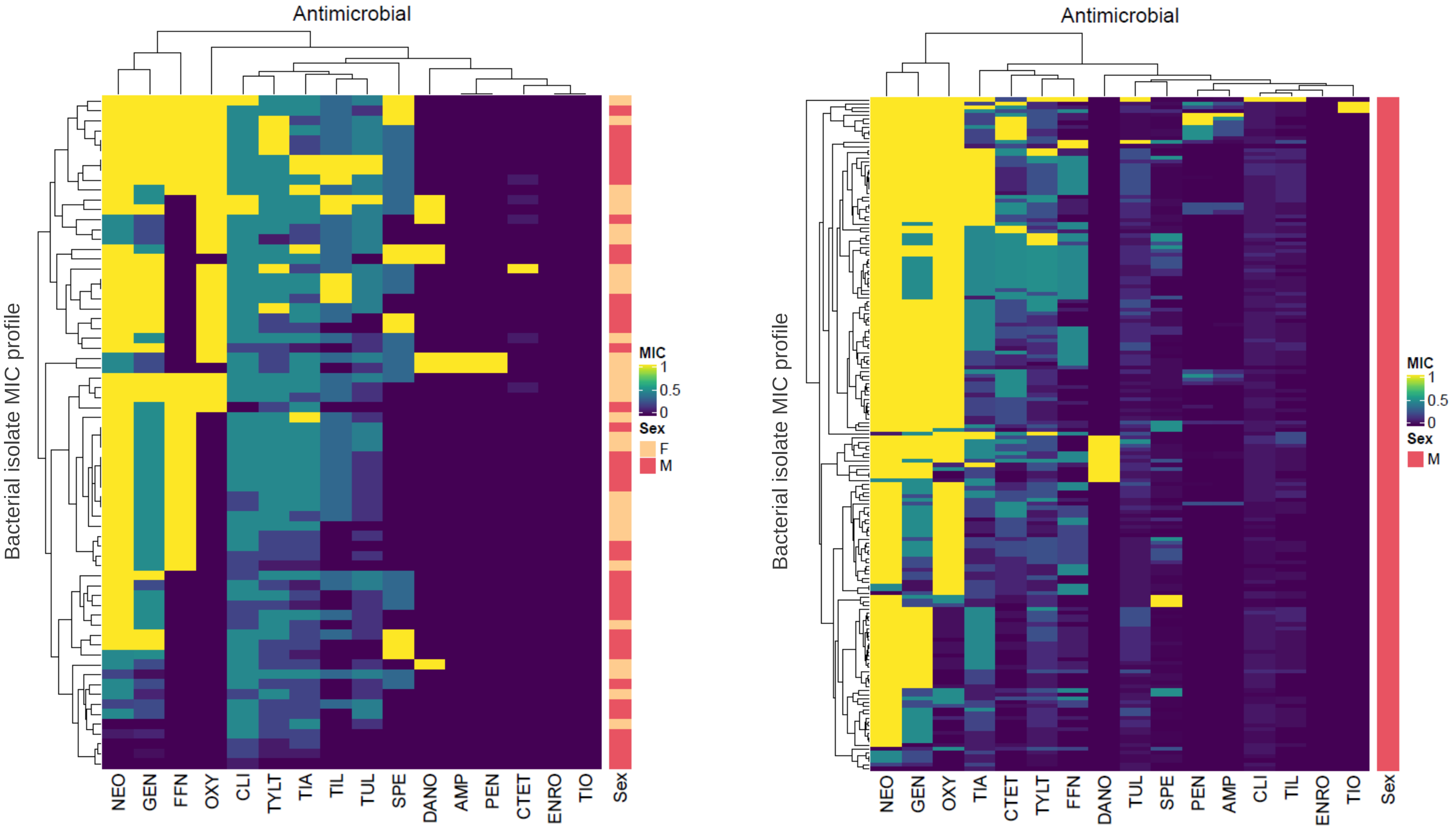

AMP, ampicillin; CLI, clindamycin; CTET, chlortetracycline; DANO, danofloxacin; ENRO, enrofloxacin; F, female; FFN, florfenicol; GEN, gentamycin; M, male; MIC, minimum inhibitory concentration; NEO, neomycin; OXY, oxytetracycline; PEN, penicillin; SPE, spectinomycin; TIA, tiamulin; TIL, tilmicosin; TIO, ceftiofur; TUL, tulathromycin; TYLT, tylosin tartrate.

Supplementary Figure 1.3.34 Unadjusted *Histophilus somni* normalized MIC distributions stratified by cattle type and clustered by age

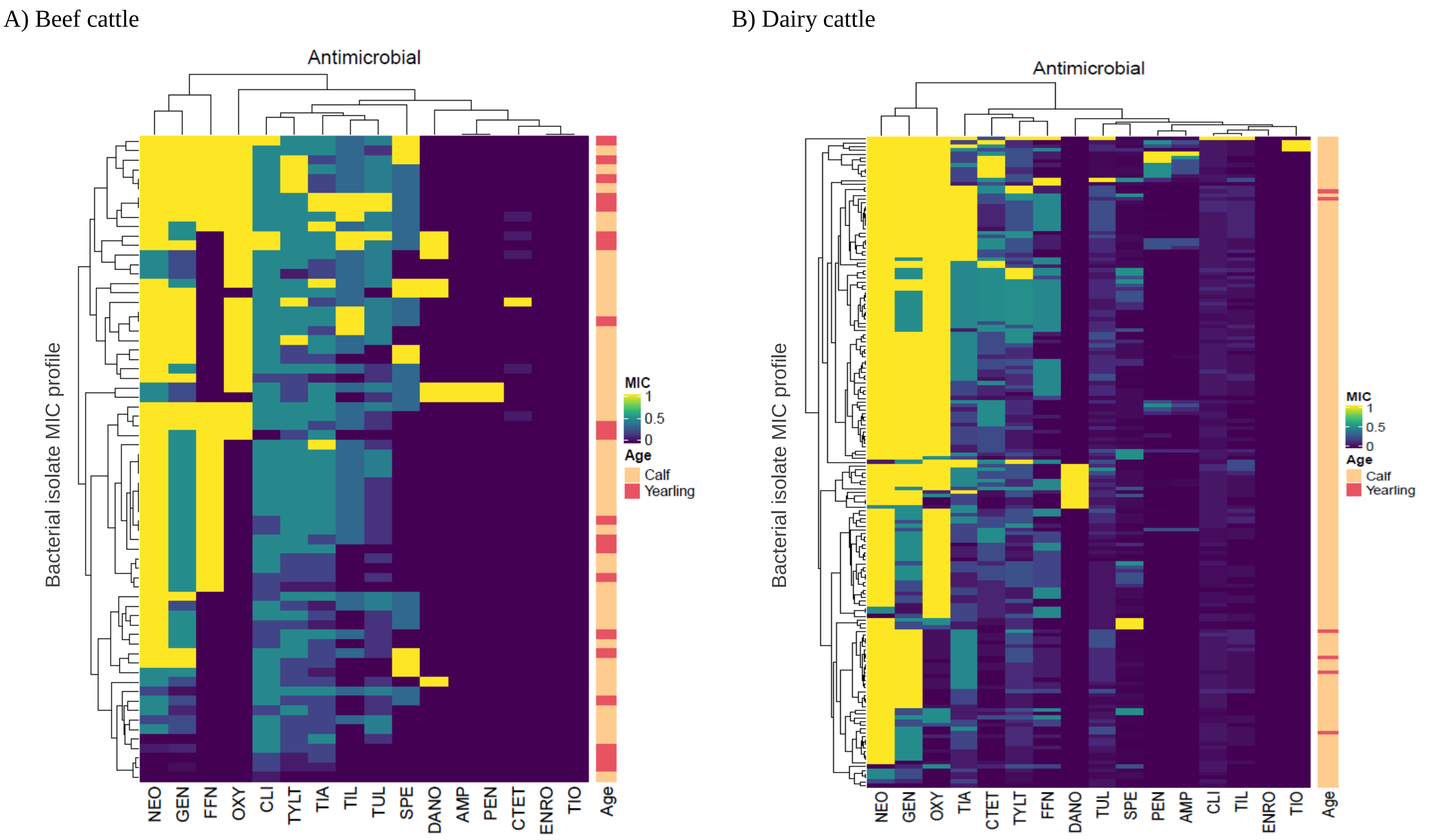

AMP, ampicillin; CLI, clindamycin; CTET, chlortetracycline; DANO, danofloxacin; ENRO, enrofloxacin; FFN, florfenicol; GEN, gentamycin; MIC, minimum inhibitory concentration; NEO, neomycin; OXY, oxytetracycline; PEN, penicillin; SPE, spectinomycin; TIA, tiamulin; TIL, tilmicosin; TIO, ceftiofur; TUL, tulathromycin; TYLT, tylosin tartrate.

Supplementary Figure 1.3.35 Unadjusted *Histophilus somni* normalized MIC distributions stratified by cattle type and clustered by temperature range

A) Beef cattle

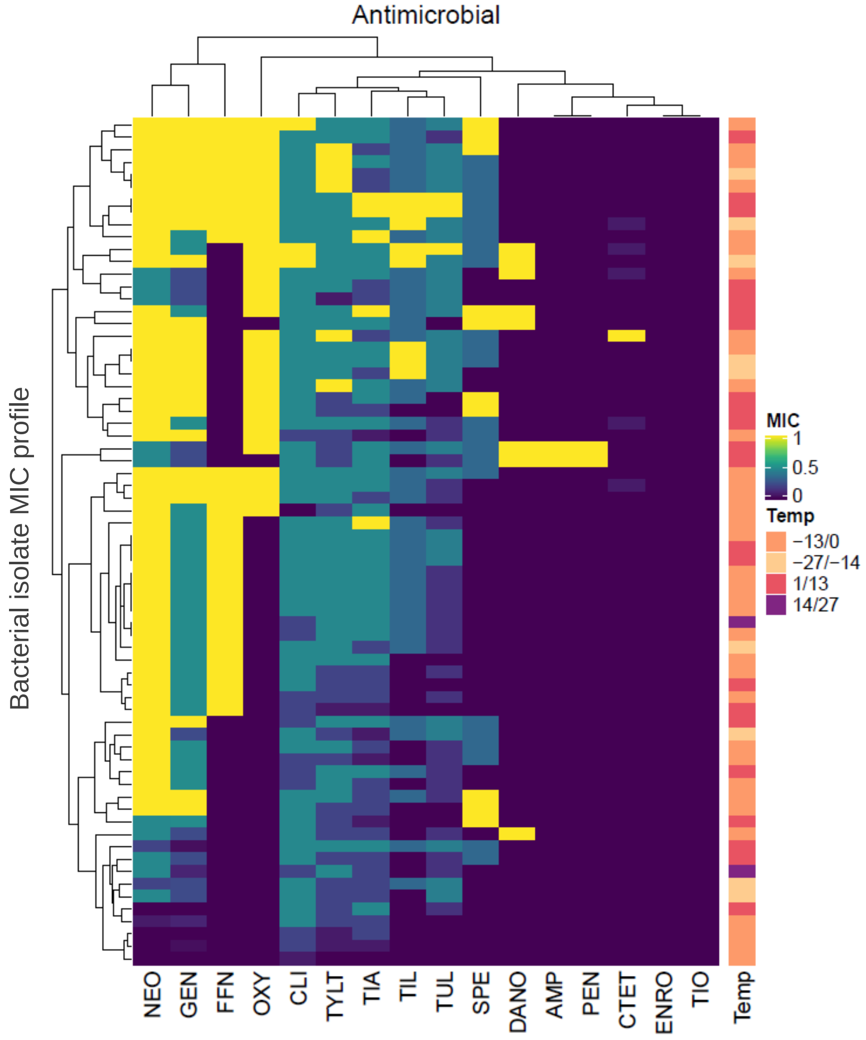

B) Dairy cattle

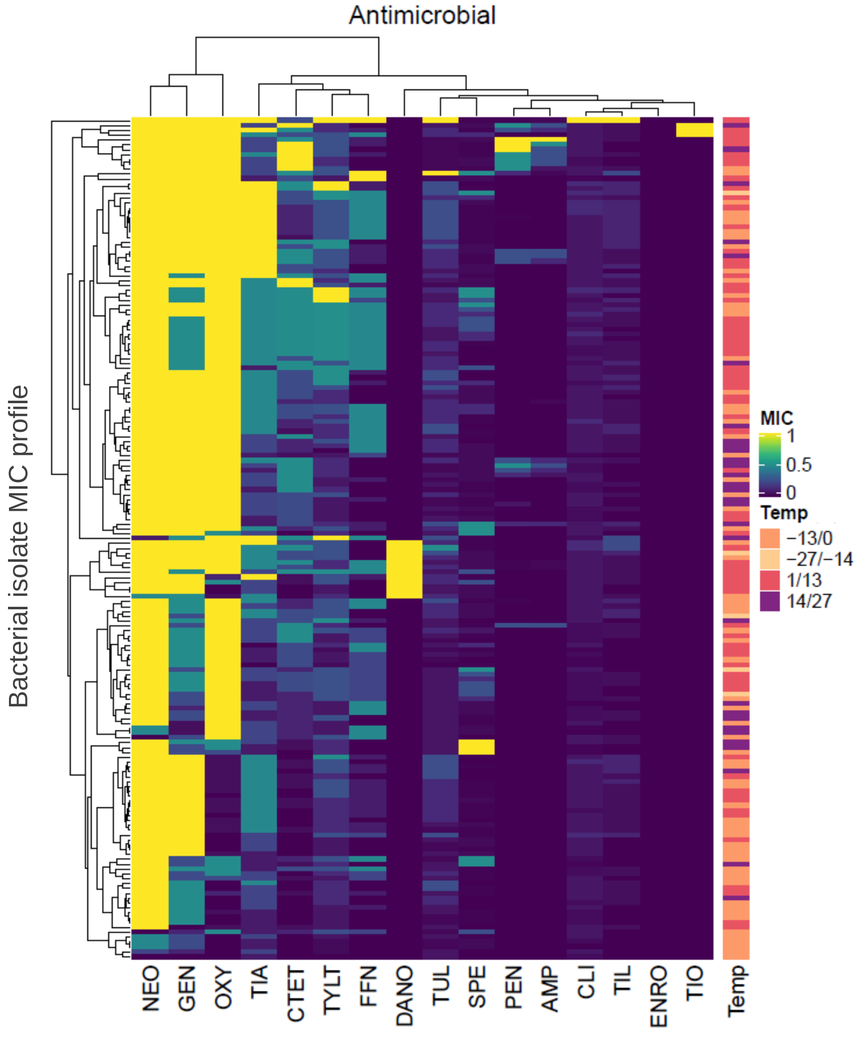

AMP, ampicillin; CLI, clindamycin; CTET, chlortetracycline; DANO, danofloxacin; ENRO, enrofloxacin; FFN, florfenicol; GEN, gentamycin; MIC, minimum inhibitory concentration; NEO, neomycin; OXY, oxytetracycline; PEN, penicillin; SPE, spectinomycin; Temp, temperature (°C); TIA, tiamulin; TIL, tilmicosin; TIO, ceftiofur; TUL, tulathromycin; TYLT, tylosin tartrate.

Supplementary Figure 1.3.36 Unadjusted *Histophilus somni* normalized MIC distributions stratified by cattle type and clustered by BRD-related morbidity

A) Beef cattle B) Dairy cattle

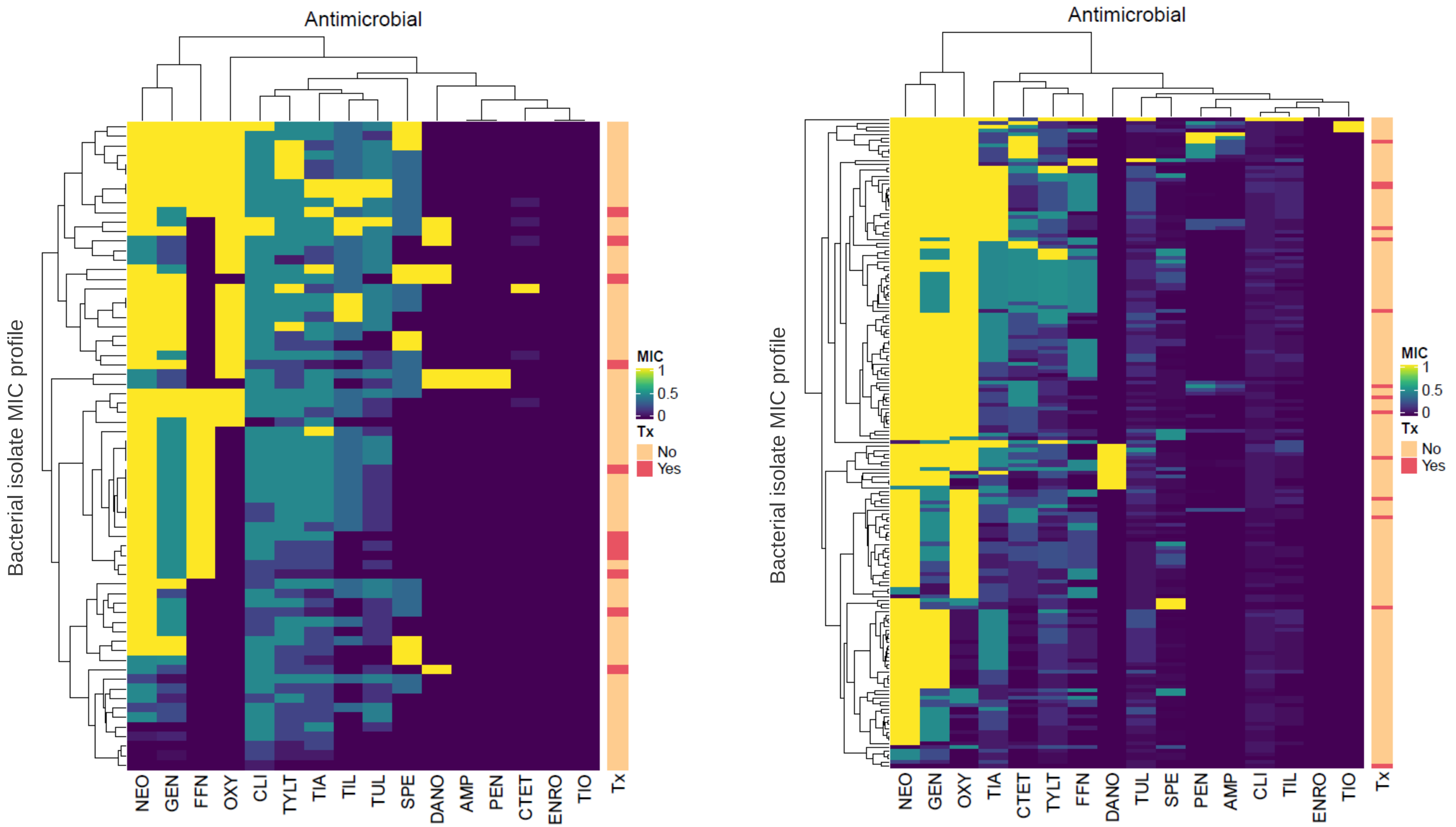

AMP, ampicillin; CLI, clindamycin; CTET, chlortetracycline; DANO, danofloxacin; ENRO, enrofloxacin; FFN, florfenicol; GEN, gentamycin; MIC, minimum inhibitory concentration; NEO, neomycin; OXY, oxytetracycline; PEN, penicillin; SPE, spectinomycin; TIA, tiamulin; TIL, tilmicosin; TIO, ceftiofur; TUL, tulathromycin; Tx, treatment; TYLT, tylosin tartrate.

Supplementary Figure 1.3.37 Unadjusted *Histophilus somni* normalized MIC distributions stratified by cattle type and clustered by BRD-related mortality

A) Beef cattle B) Dairy cattle

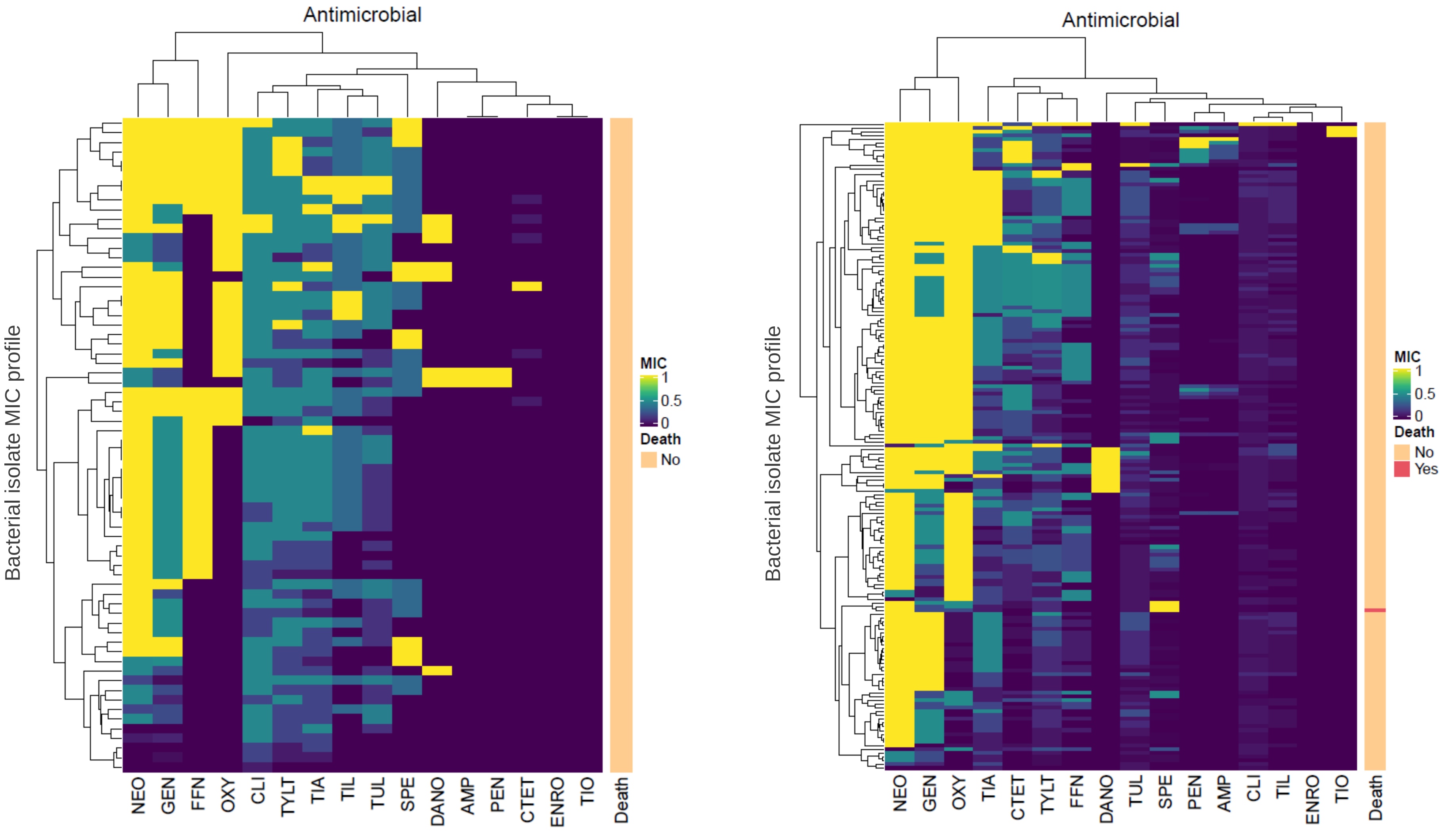

AMP, ampicillin; CLI, clindamycin; CTET, chlortetracycline; DANO, danofloxacin; ENRO, enrofloxacin; FFN, florfenicol; GEN, gentamycin; MIC, minimum inhibitory concentration; NEO, neomycin; OXY, oxytetracycline; PEN, penicillin; SPE, spectinomycin; TIA, tiamulin; TIL, tilmicosin; TIO, ceftiofur; TUL, tulathromycin; TYLT, tylosin tartrate.

Supplementary Figure 1.3.38 Unadjusted *Histophilus somni* normalized MIC distributions stratified by cattle type and clustered by feedlot

A) Beef cattle

B) Dairy cattle

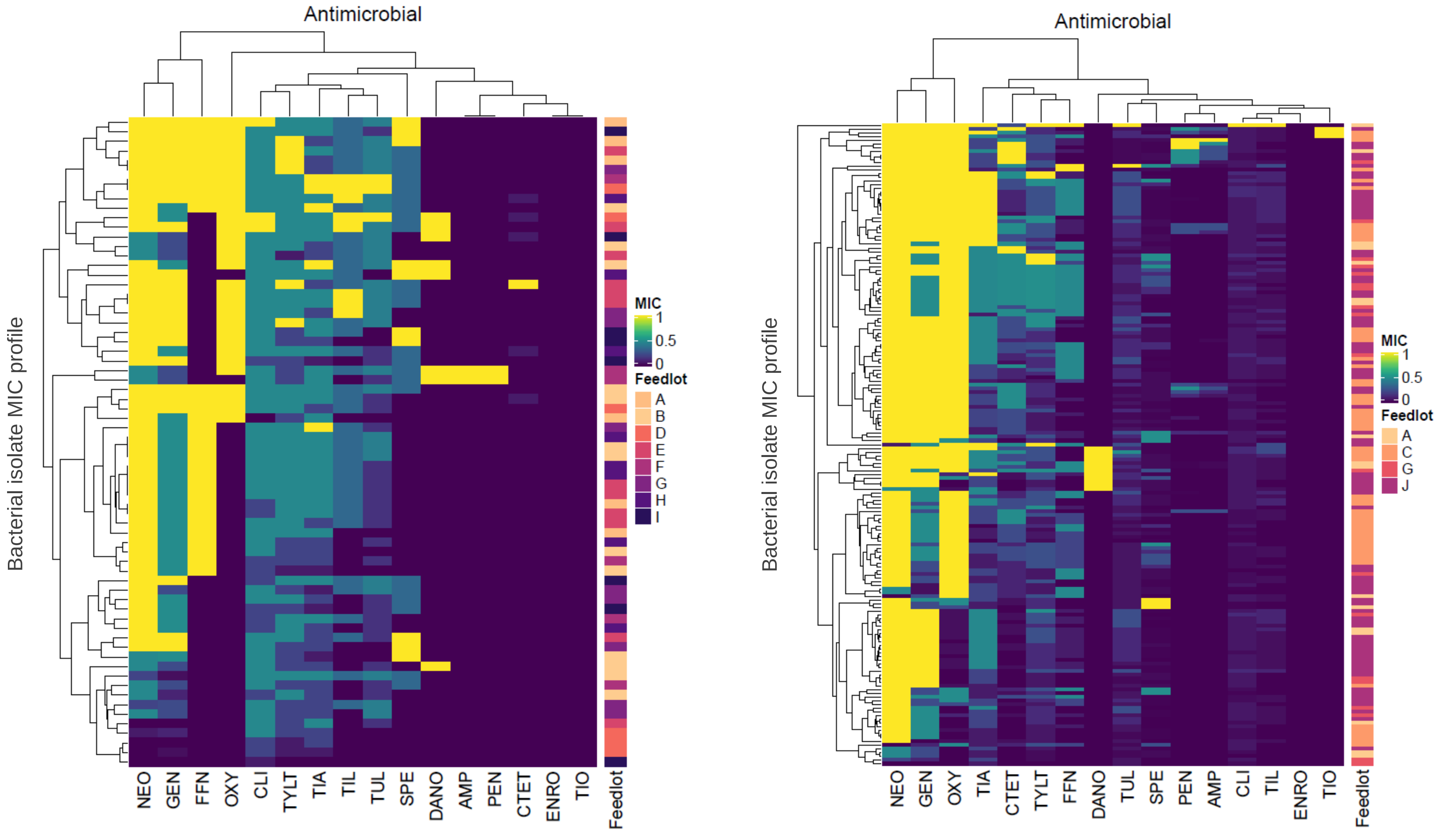

AMP, ampicillin; CLI, clindamycin; CTET, chlortetracycline; DANO, danofloxacin; ENRO, enrofloxacin; FFN, florfenicol; GEN, gentamycin; MIC, minimum inhibitory concentration; NEO, neomycin; OXY, oxytetracycline; PEN, penicillin; SPE, spectinomycin; TIA, tiamulin; TIL, tilmicosin; TIO, ceftiofur; TUL, tulathromycin; TYLT, tylosin tartrate.

Supplementary Figure 1.3.39 Unadjusted *Mycoplasma bovis* normalized MIC distributions stratified by cattle type and clustered by country of origin

A) Beef cattle

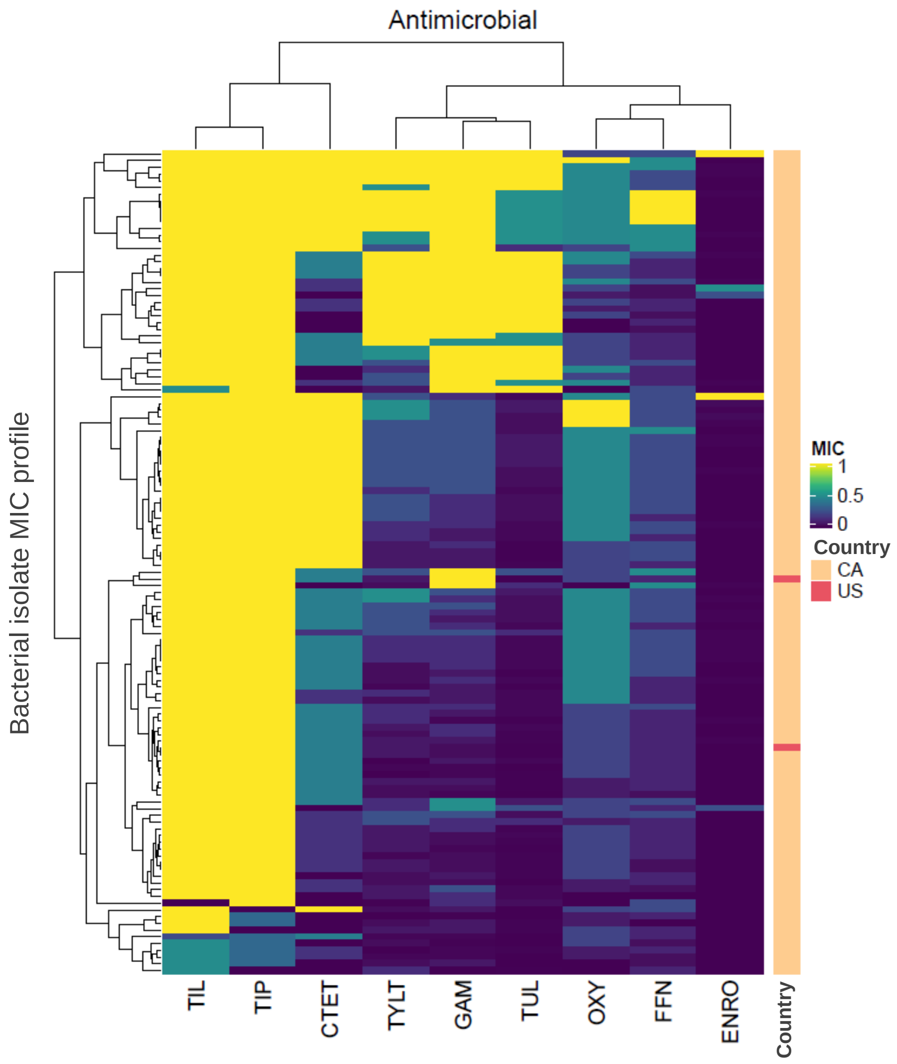

B) Dairy cattle

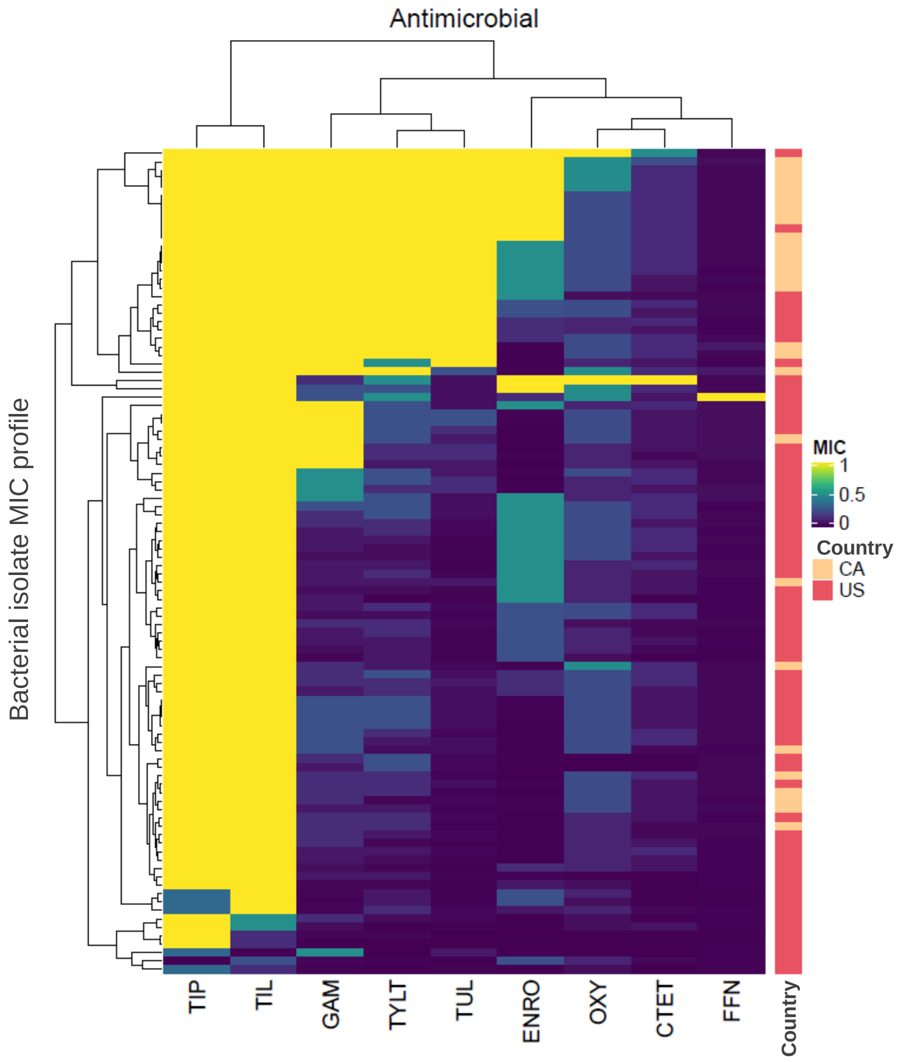

CA, Canada; CTET, chlortetracycline; ENRO, enrofloxacin; FFN, florfenicol; GAM, gamithromycin; MIC, minimum inhibitory concentration; OXY, oxytetracycline; TIL, tilmicosin; TIP, tildipirosin; TUL, tulathromycin; TYLT, tylosin tartrate.

Supplementary Figure 1.3.40 Unadjusted *Mycoplasma bovis* normalized MIC distributions stratified by cattle type and clustered by monthly interval

A) Beef cattle

B) Dairy cattle

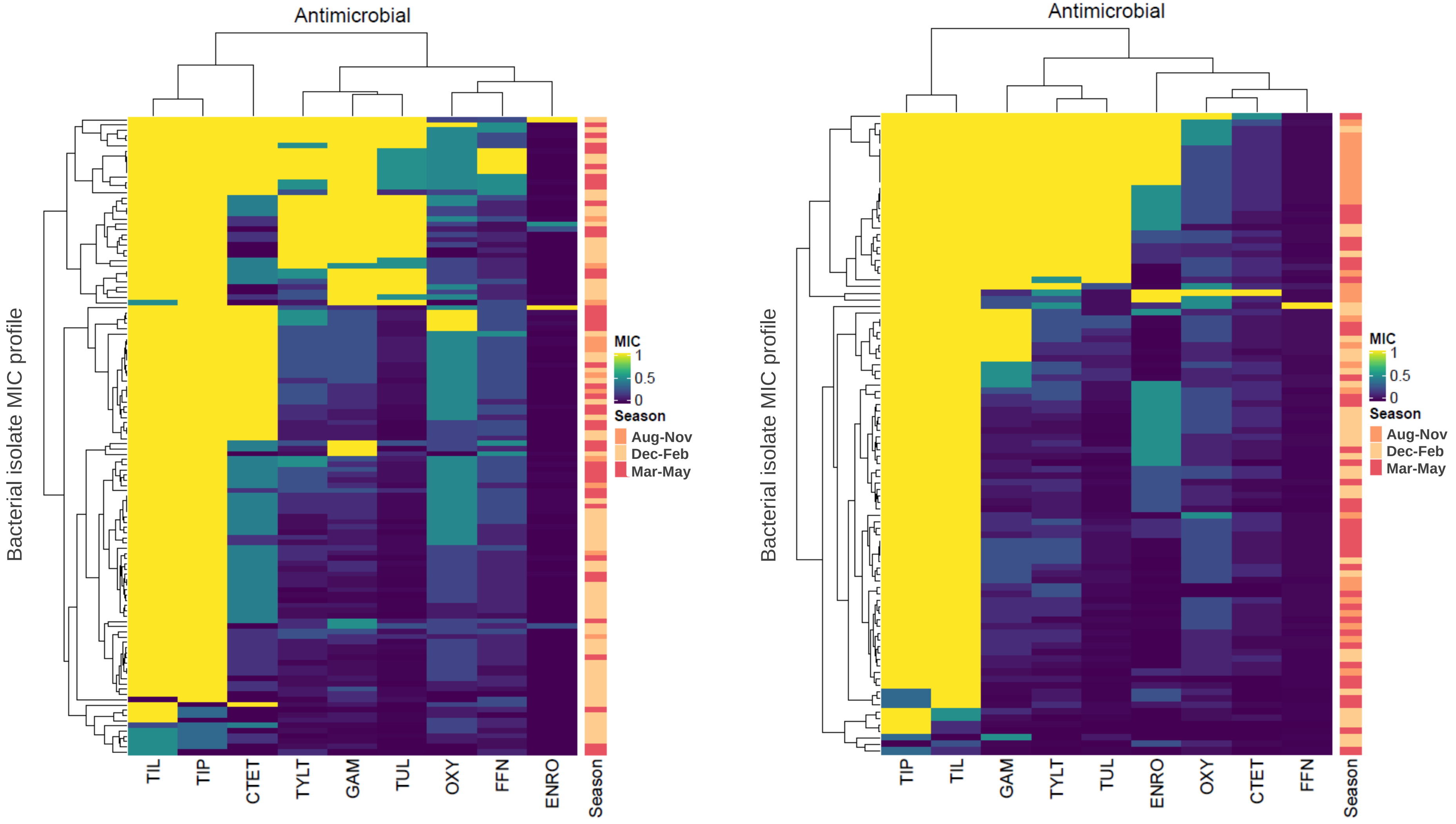

CTET, chlortetracycline; ENRO, enrofloxacin; FFN, florfenicol; GAM, gamithromycin; MIC, minimum inhibitory concentration; OXY, oxytetracycline; TIL, tilmicosin; TIP, tildipirosin; TUL, tulathromycin; TYLT, tylosin tartrate.

Supplementary Figure 1.3.41 Unadjusted *Mycoplasma bovis* normalized MIC distributions stratified by cattle type and clustered by source

A) Beef cattle

B) Dairy cattle

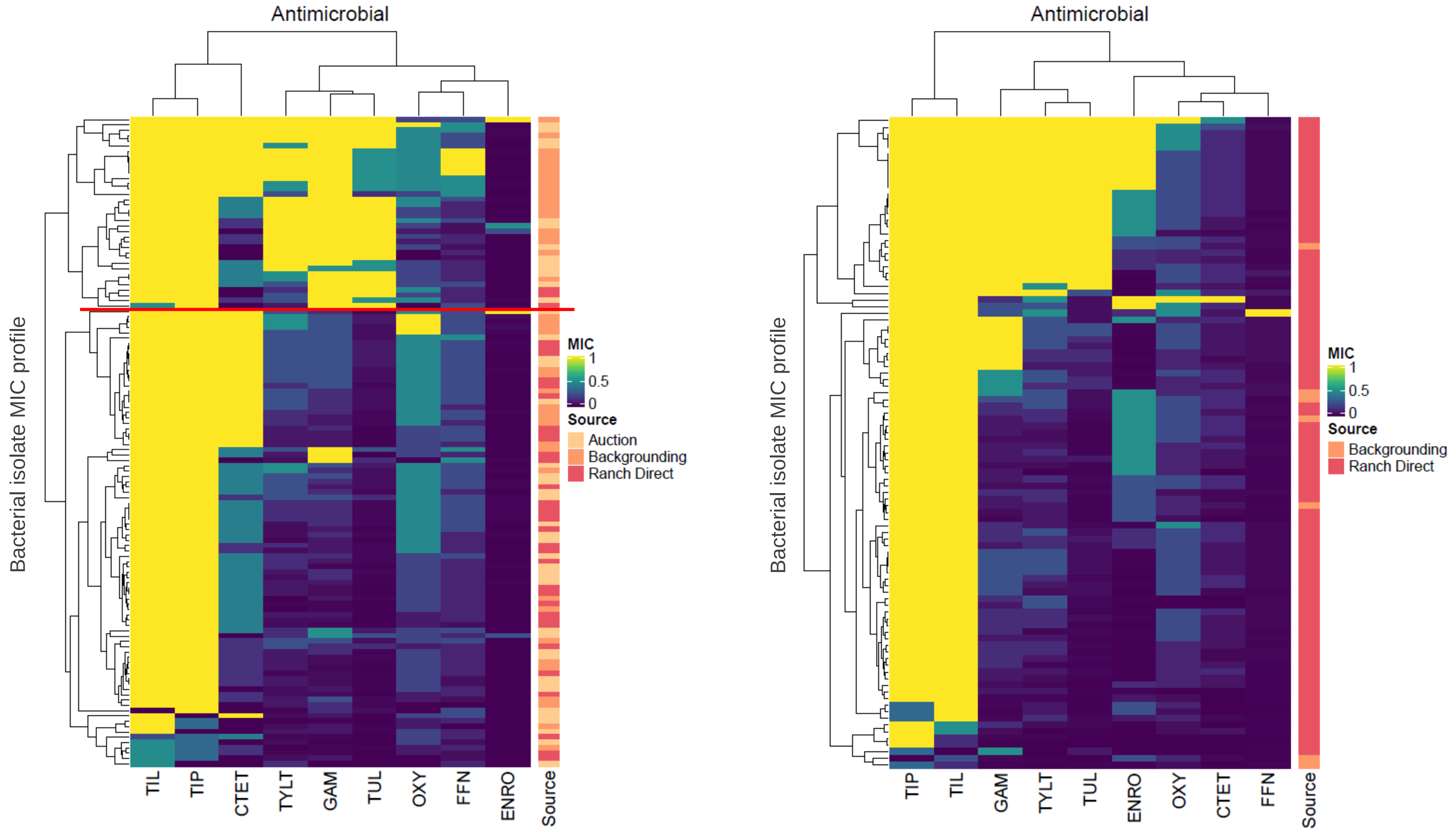

Backgrounding operations; CTET, chlortetracycline; ENRO, enrofloxacin; FFN, florfenicol; GAM, gamithromycin; MIC, minimum inhibitory concentration; OXY, oxytetracycline; TIL, tilmicosin; TIP, tildipirosin; TUL, tulathromycin; TYLT, tylosin tartrate. The red line defines two different bacterial populations grouped by antimicrobial susceptibility profiles.

Supplementary Figure 1.3.42 Unadjusted *Mycoplasma bovis* normalized MIC distributions stratified by cattle type and clustered by risk of suffering BRD

A) Beef cattle B) Dairy cattle

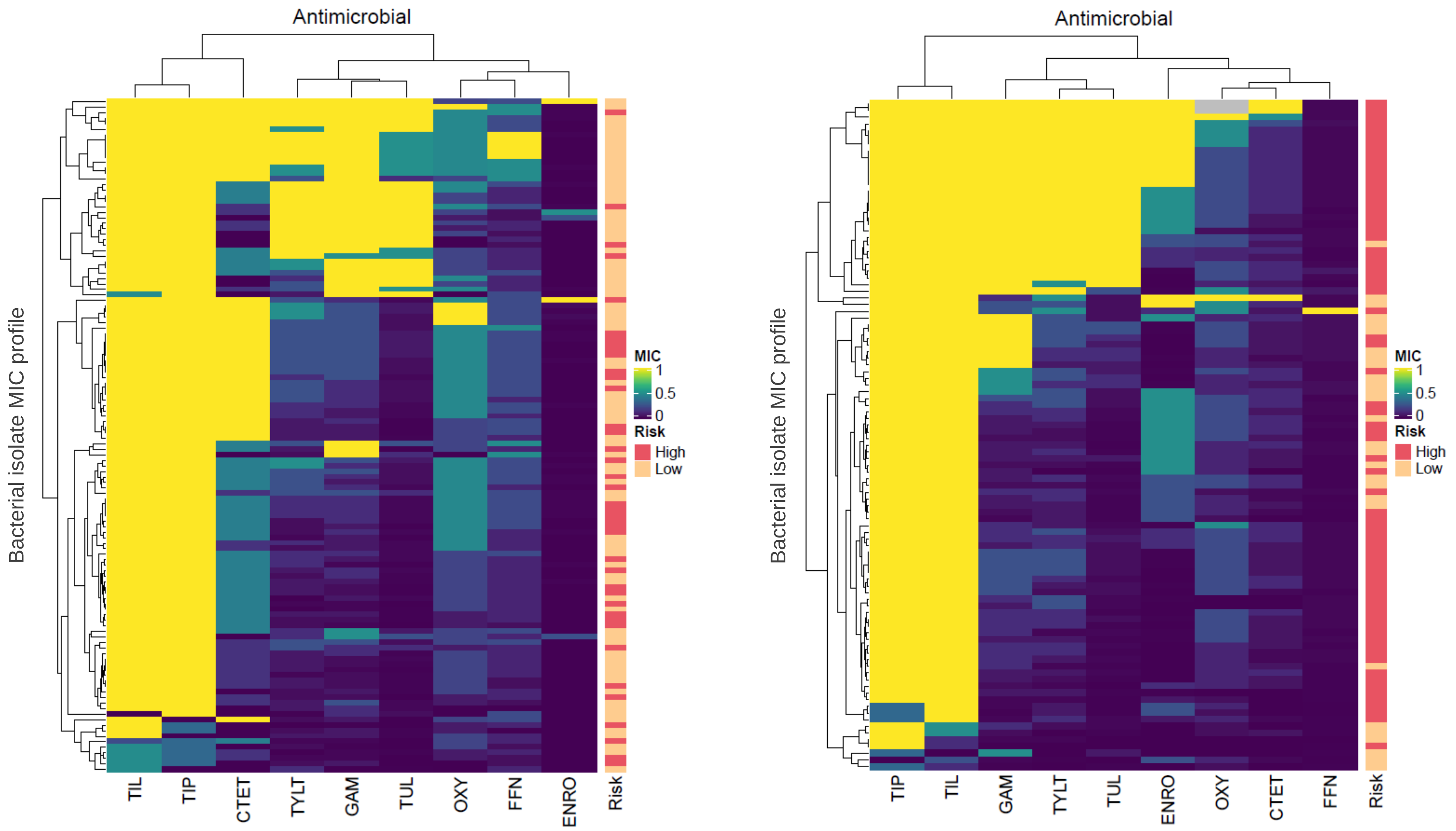

CTET, chlortetracycline; ENRO, enrofloxacin; FFN, florfenicol; GAM, gamithromycin; MIC, minimum inhibitory concentration; OXY, oxytetracycline; TIL, tilmicosin; TIP, tildipirosin; TUL, tulathromycin; TYLT, tylosin tartrate.

Supplementary Figure 1.3.43 Unadjusted *Mycoplasma bovis* normalized MIC distributions stratified by cattle type and clustered by weight range

A) Beef cattle

B) Dairy cattle

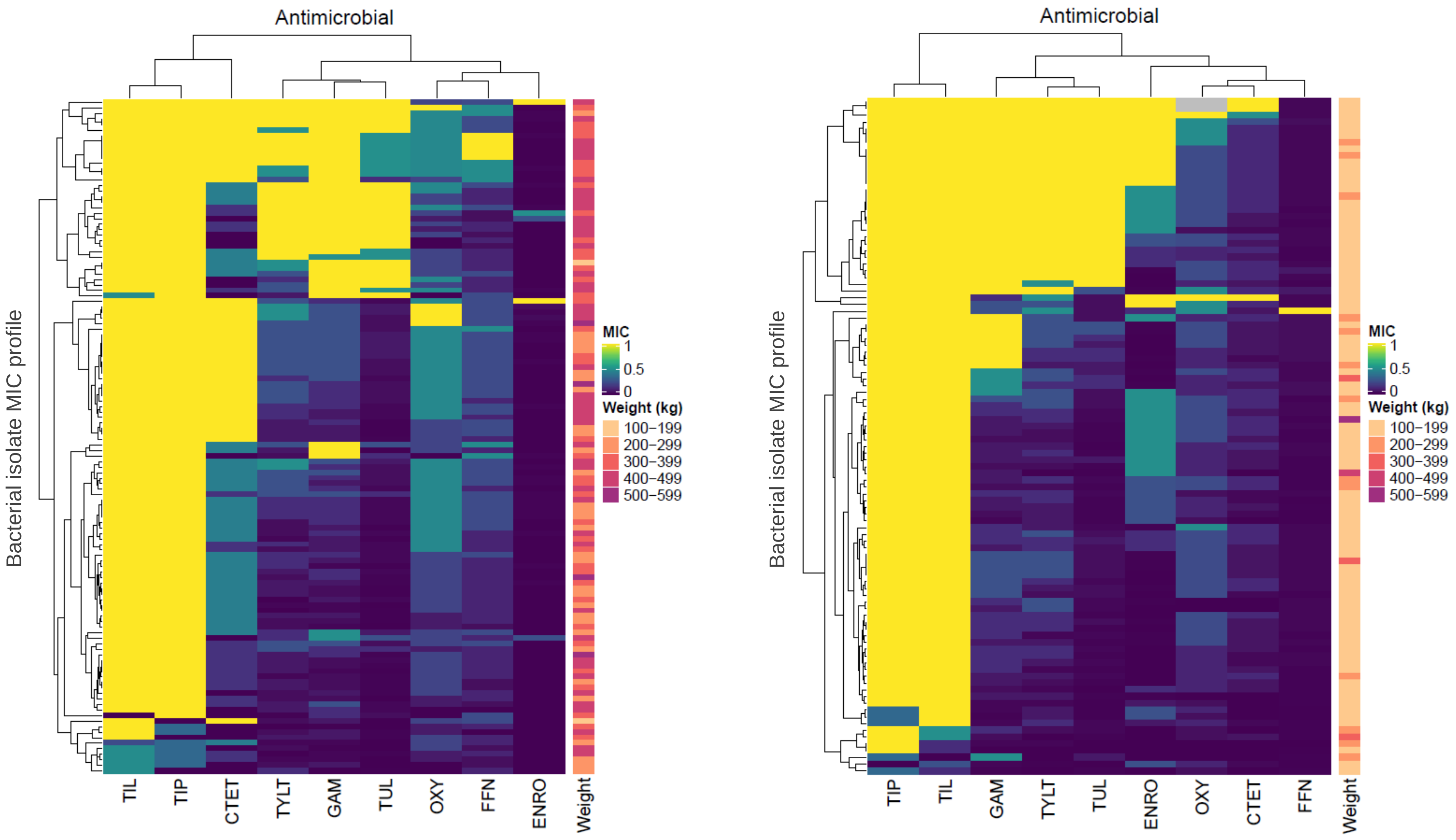

CTET, chlortetracycline; ENRO, enrofloxacin; FFN, florfenicol; GAM, gamithromycin; MIC, minimum inhibitory concentration; OXY, oxytetracycline; TIL, tilmicosin; TIP, tildipirosin; TUL, tulathromycin; TYLT, tylosin tartrate.

Supplementary Figure 1.3.44 Unadjusted *Mycoplasma bovis* normalized MIC distributions stratified by cattle type and clustered by sex

A) Beef cattle B) Dairy cattle

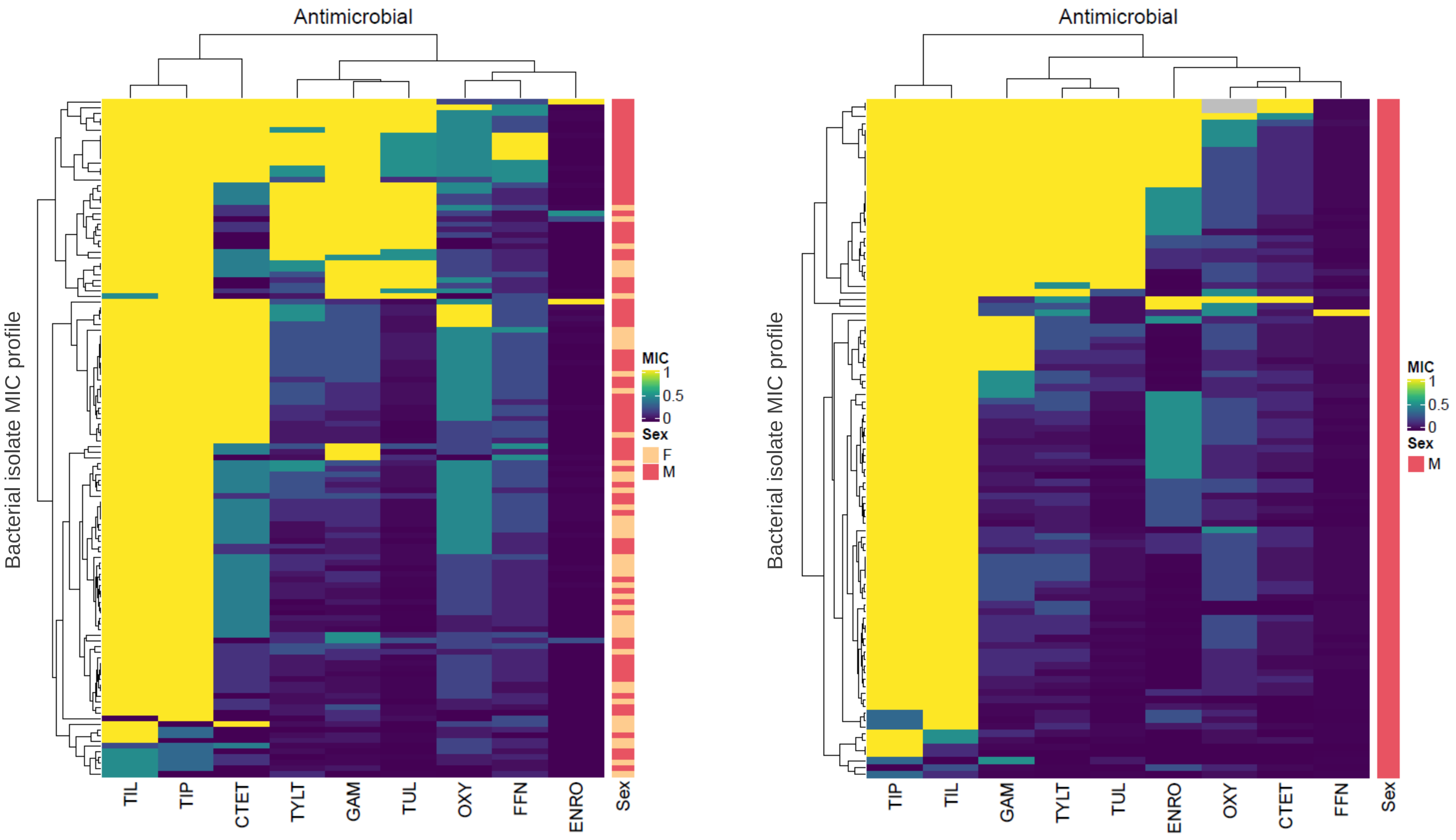

CTET, chlortetracycline; ENRO, enrofloxacin; F, female; FFN, florfenicol; GAM, gamithromycin; M, male; MIC, minimum inhibitory concentration; OXY, oxytetracycline; TIL, tilmicosin; TIP, tildipirosin; TUL, tulathromycin; TYLT, tylosin tartrate.

Supplementary Figure 1.3.45 Unadjusted *Mycoplasma bovis* normalized MIC distributions stratified by cattle type and clustered by age

A) Beef cattle

B) Dairy cattle

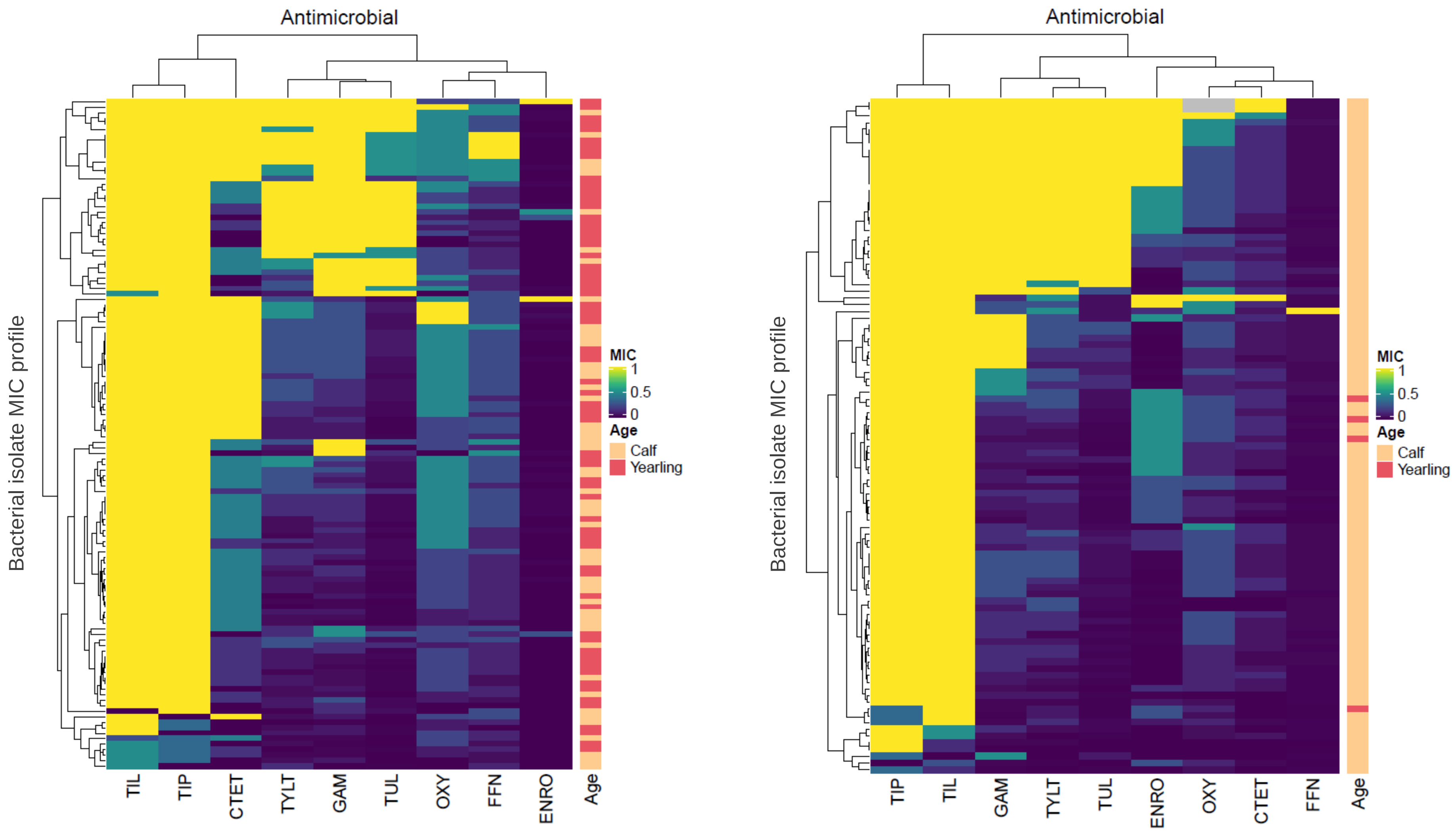

CTET, chlortetracycline; ENRO, enrofloxacin; FFN, florfenicol; GAM, gamithromycin; MIC, minimum inhibitory concentration; OXY, oxytetracycline; TIL, tilmicosin; TIP, tildipirosin; TUL, tulathromycin; TYLT, tylosin tartrate.

Supplementary Figure 1.3.46 Unadjusted *Mycoplasma bovis* normalized MIC distributions stratified by cattle type and clustered by temperature range

A) Beef cattle

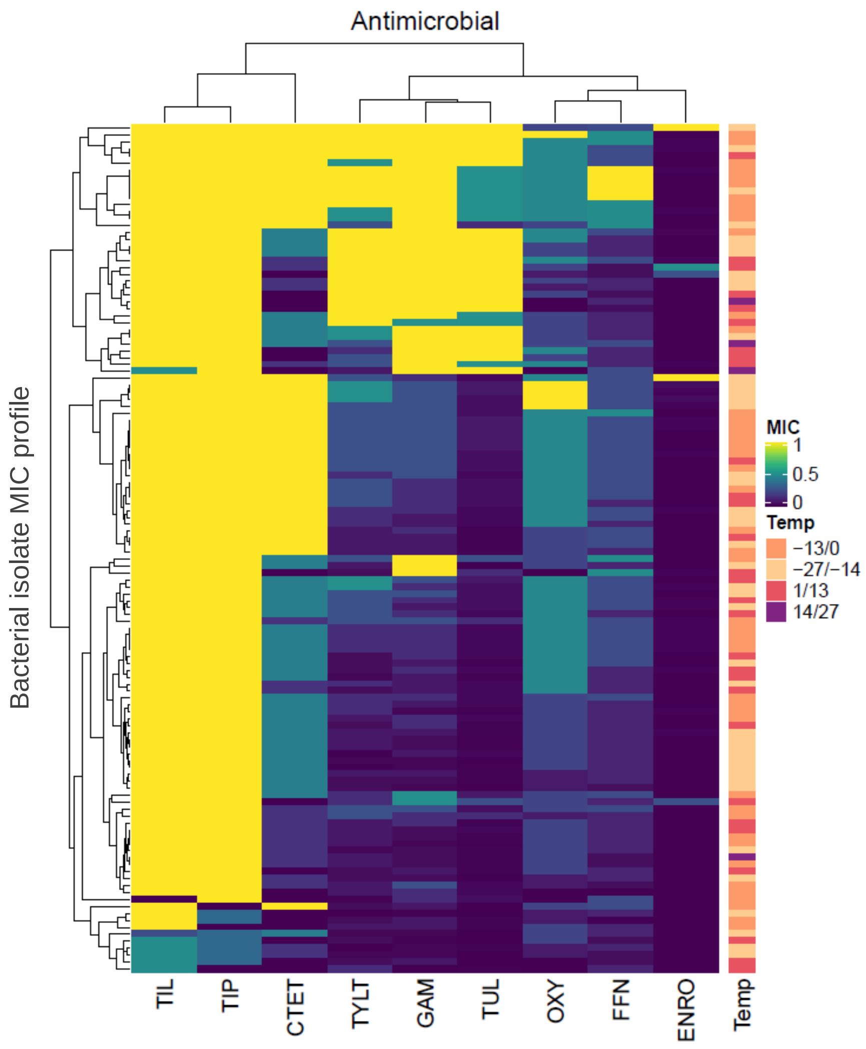

B) Dairy cattle

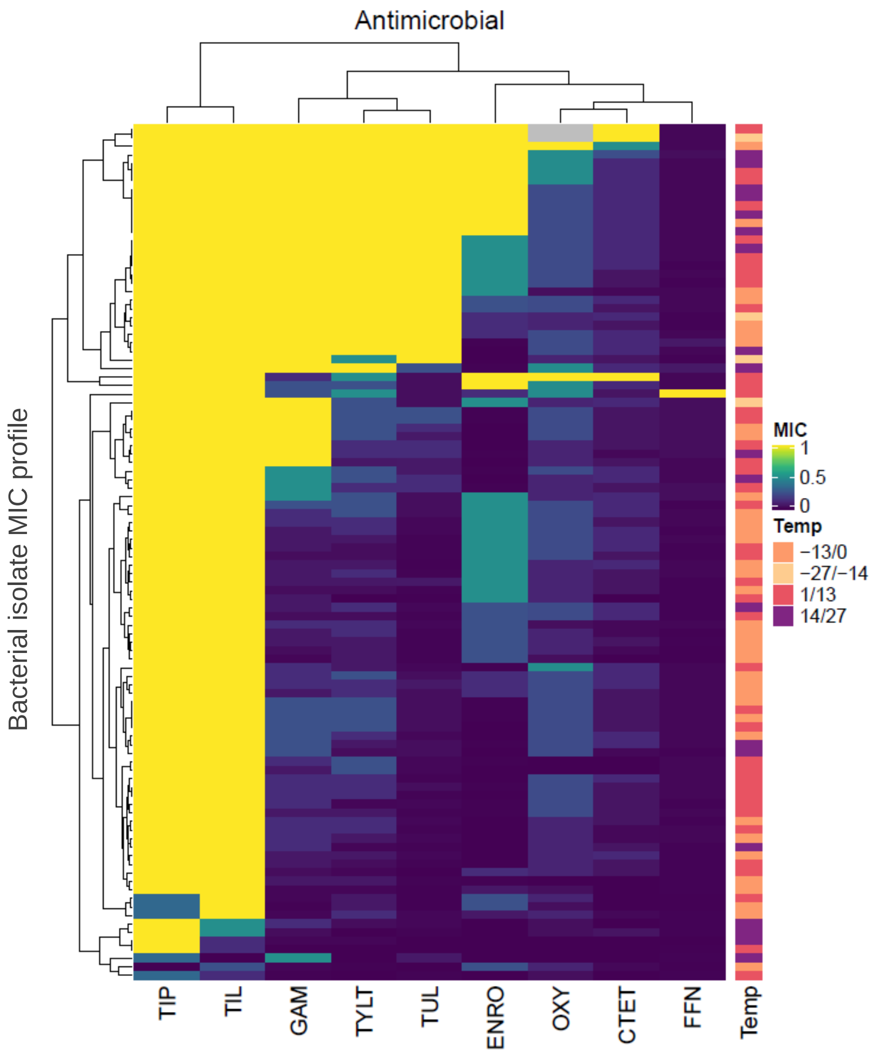

CTET, chlortetracycline; ENRO, enrofloxacin; FFN, florfenicol; GAM, gamithromycin; MIC, minimum inhibitory concentration; OXY, oxytetracycline; Temp, temperature (°C); TIL, tilmicosin; TIP, tildipirosin; TUL, tulathromycin; TYLT, tylosin tartrate.

Supplementary Figure 1.3.47 Unadjusted *Mycoplasma bovis* normalized MIC distributions stratified by cattle type and clustered by BRD-related morbidity

A) Beef cattle B) Dairy cattle

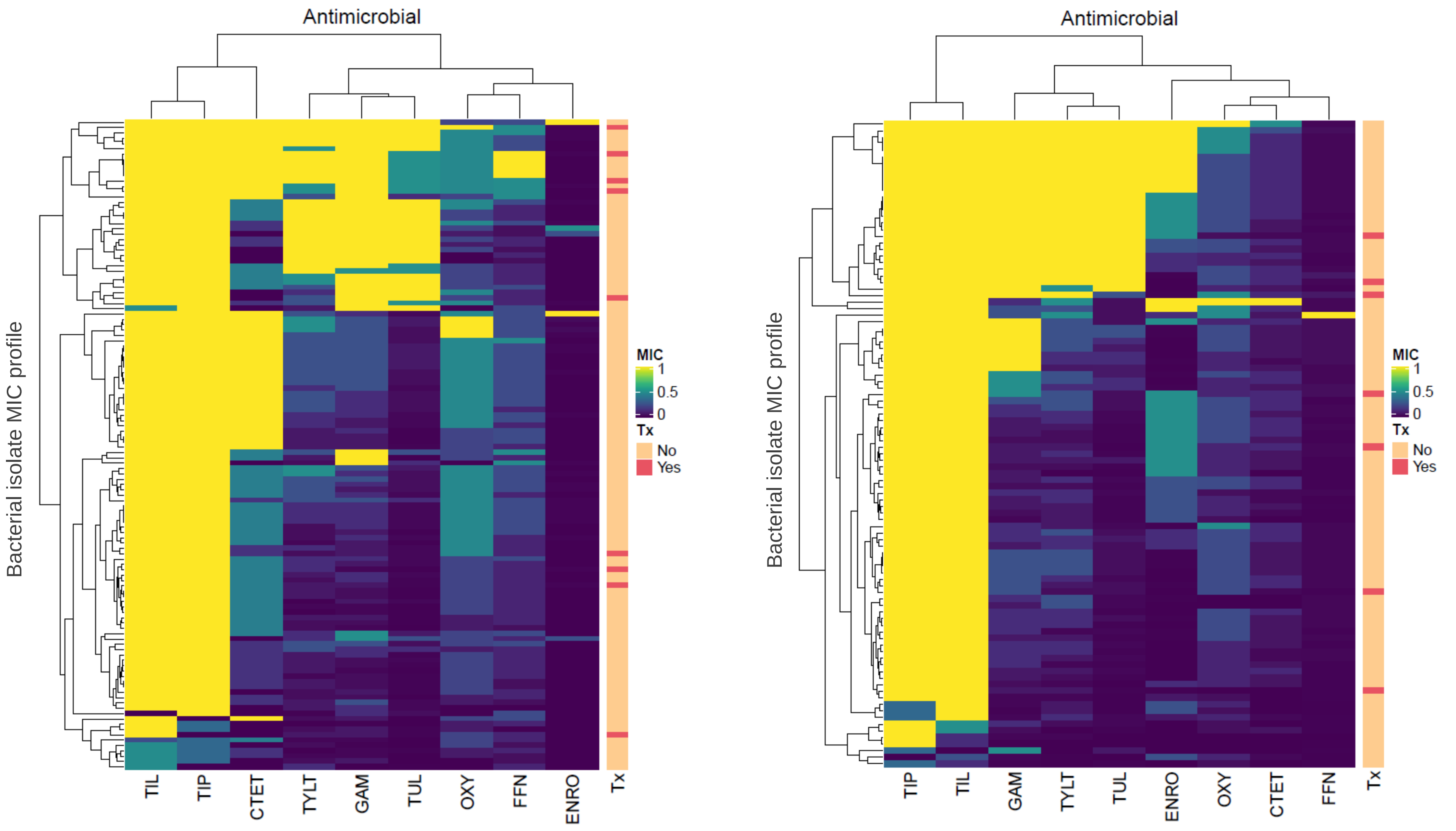

CTET, chlortetracycline; ENRO, enrofloxacin; FFN, florfenicol; GAM, gamithromycin; MIC, minimum inhibitory concentration; OXY, oxytetracycline; TIL, tilmicosin; TIP, tildipirosin; TUL, tulathromycin; Tx, treatment; TYLT, tylosin tartrate.

Supplementary Figure 1.3.48 Unadjusted *Mycoplasma bovis* normalized MIC distributions stratified by cattle type and clustered by BRD-related mortality

A) Beef cattle

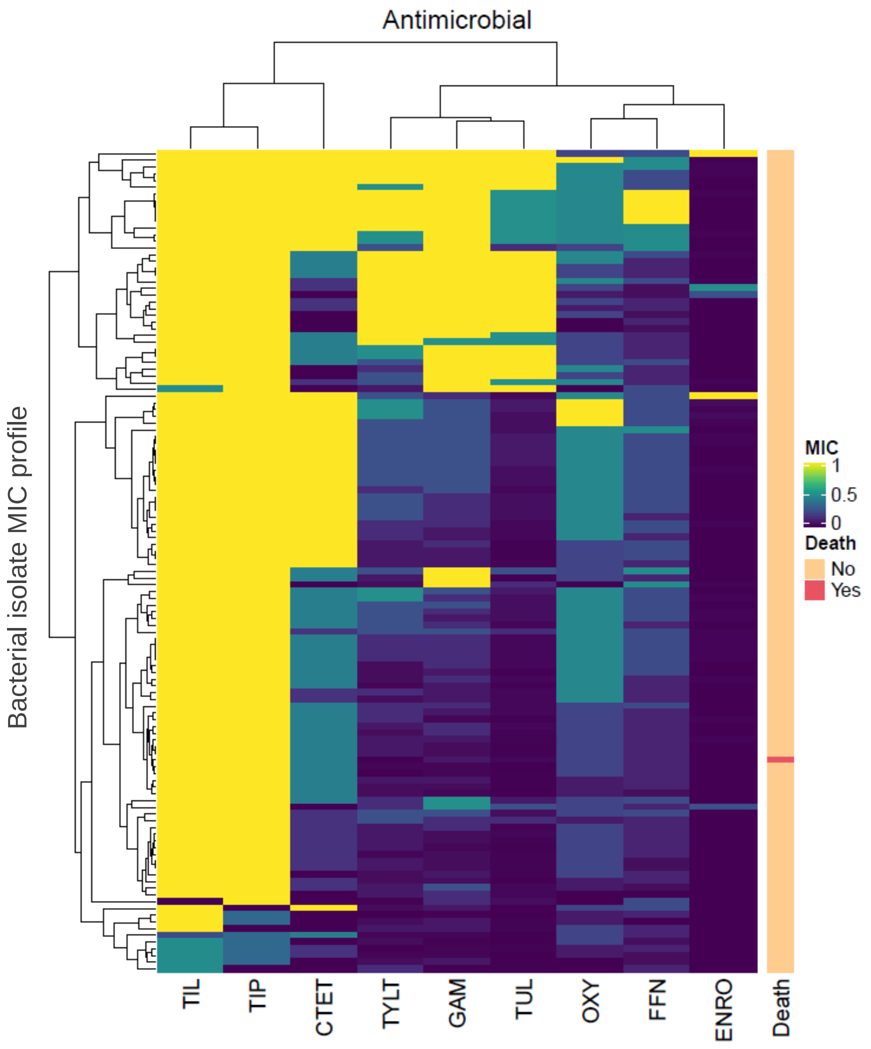

B) Dairy cattle

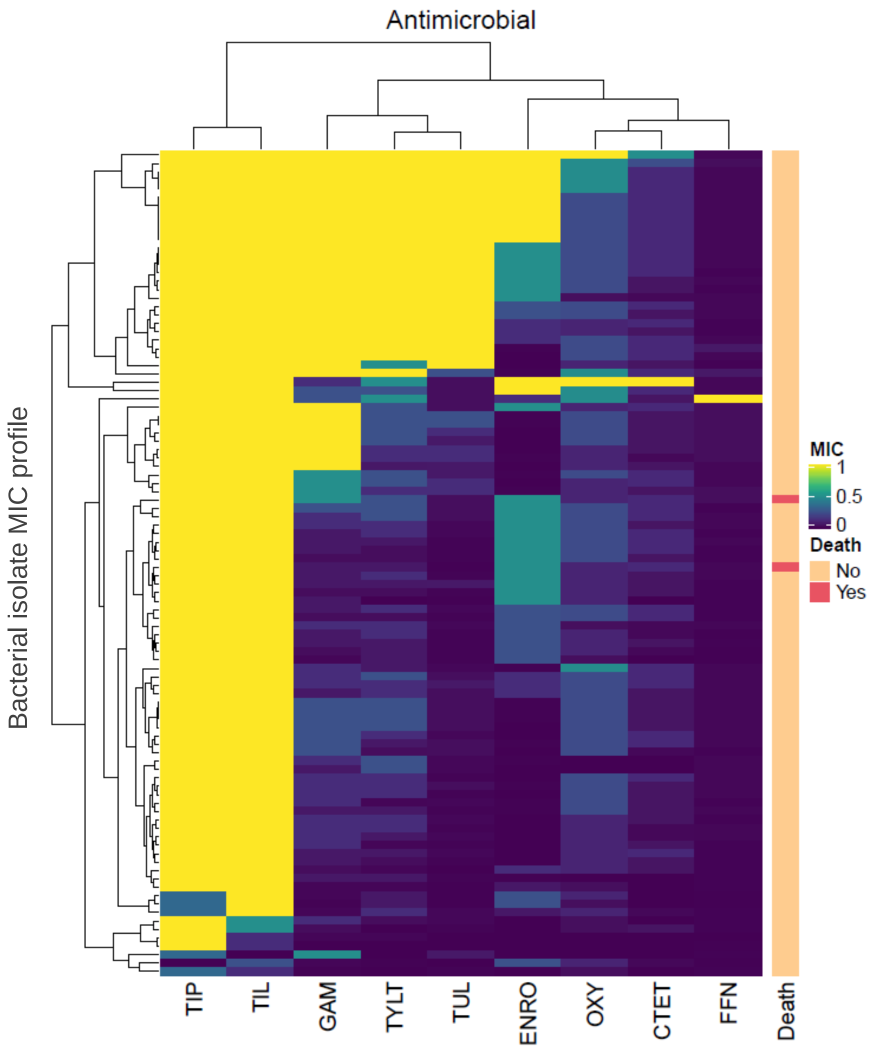

CTET, chlortetracycline; ENRO, enrofloxacin; FFN, florfenicol; GAM, gamithromycin; MIC, minimum inhibitory concentration; OXY, oxytetracycline; TIL, tilmicosin; TIP, tildipirosin; TUL, tulathromycin; TYLT, tylosin tartrate.

Supplementary Figure 1.3.49 Unadjusted *Mycoplasma bovis* normalized MIC distributions stratified by cattle type and clustered by feedlot

A) Beef cattle B) Dairy cattle

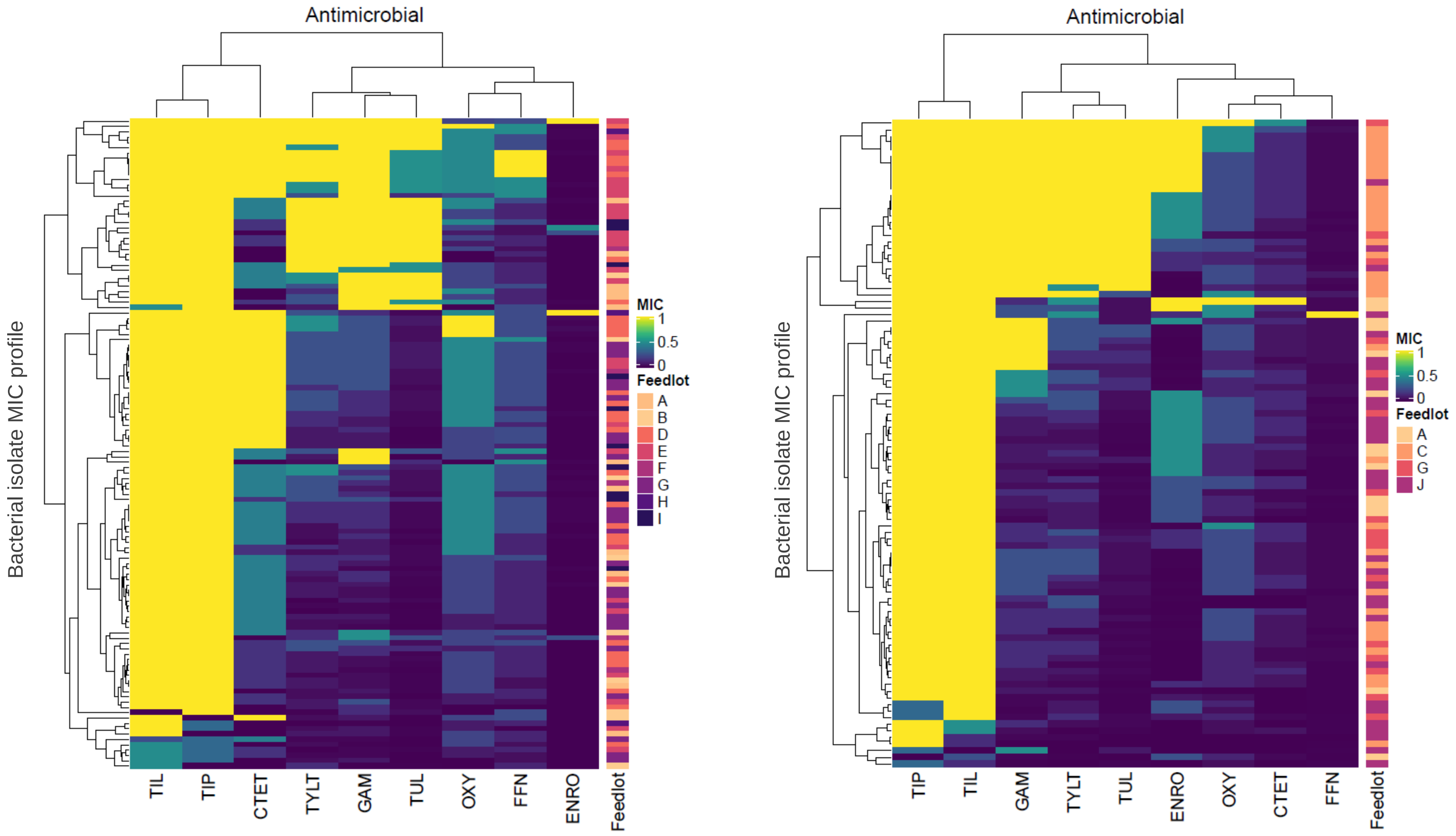

CTET, chlortetracycline; ENRO, enrofloxacin; FFN, florfenicol; GAM, gamithromycin; MIC, minimum inhibitory concentration; OXY, oxytetracycline; TIL, tilmicosin; TIP, tildipirosin; TUL, tulathromycin; TYLT, tylosin tartrate.

Supplementary Figure 1.3.50 Dairy *Mannheimia haemolytica* susceptibilities stratified by feedlot and clustered by source, arrived from, and truck load

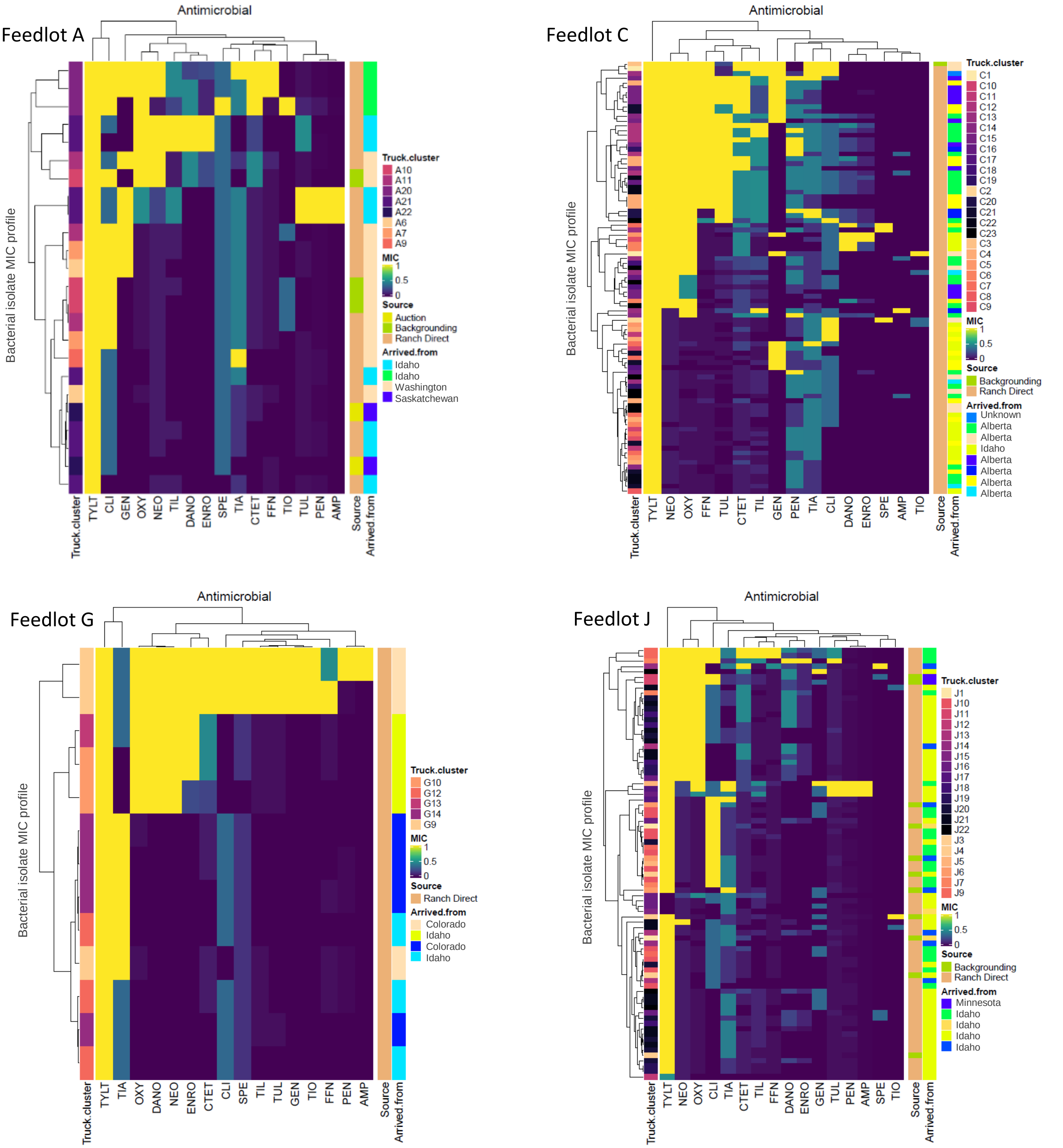

The specific names of the location of origin of cattle is not specified because of privacy matters. Province or state are provided instead. Backgrounding operations.

Supplementary Figure 1.3.51 Dairy *Mycoplasma bovis* susceptibilities stratified by feedlot and clustered by source, arrived from, and truck load

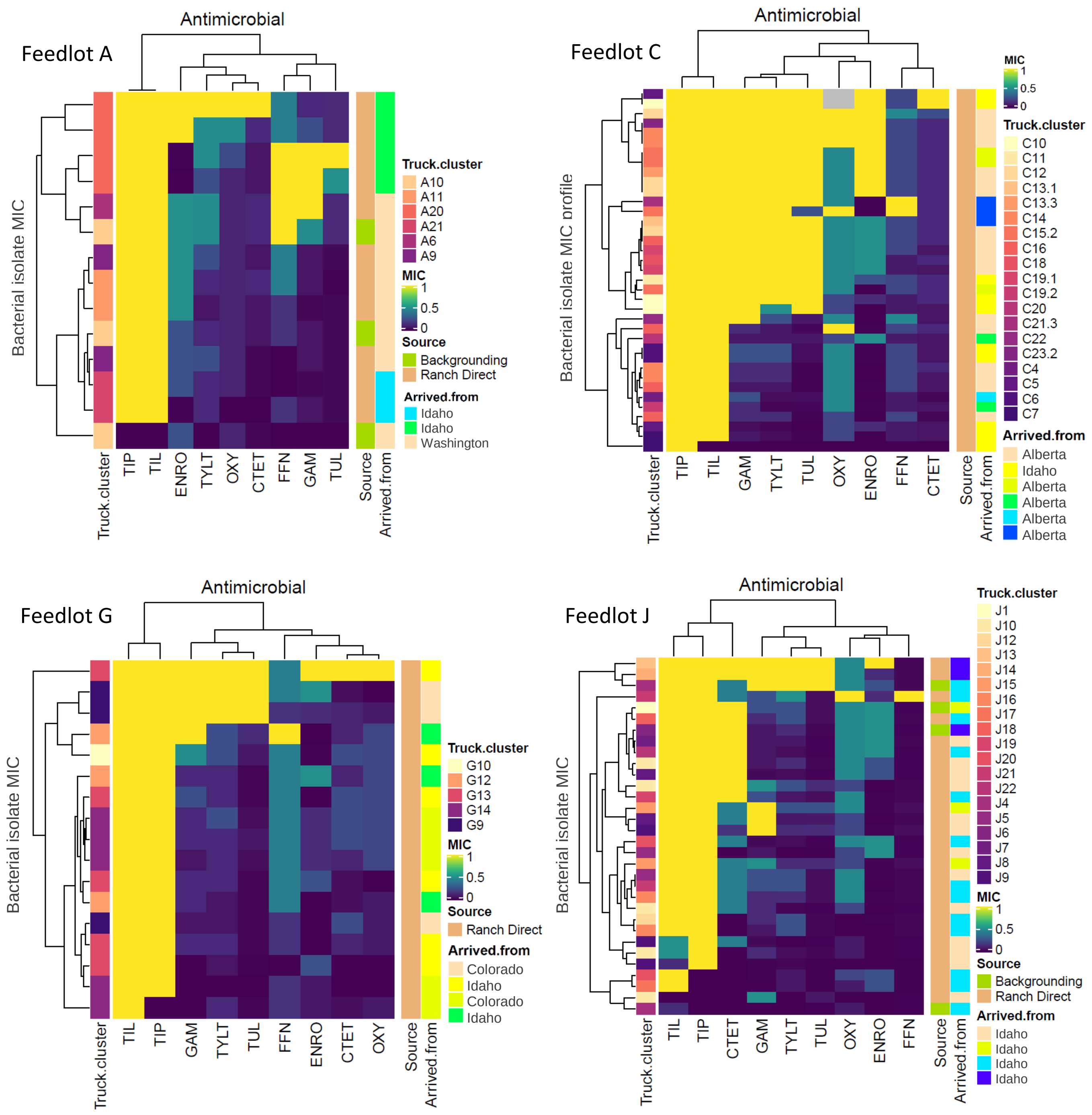

The specific names of the location of origin of cattle is not specified because of privacy matters. Province or state are provided instead. Backgrounding operations.
